# Incidence, Risk Factors, and Temporal Trends of Tongue Cancer: A Population‐Based Study

**DOI:** 10.1002/cam4.71435

**Published:** 2026-02-03

**Authors:** Junjie Huang, Wing Sze Pang, Claire Chenwen Zhong, Fung Yu Mak, Sze Chai Chan, Jinqiu Yuan, Lin Zhang, Wanghong Xu, Zhi‐Jie Zheng, Zigui Chen, Jason Y. K. Chan, Martin C. S. Wong

**Affiliations:** ^1^ The Jockey Club School of Public Health and Primary Care, Faculty of Medicine Chinese University of Hong Kong Hong Kong SAR China; ^2^ Centre for Health Education and Health Promotion, Faculty of Medicine The Chinese University of Hong Kong Hong Kong SAR China; ^3^ Clinical Research Center, Big Data Center, the Seventh Affiliated Hospital Sun Yat‐Sen University Shenzhen Guangdong China; ^4^ The School of Public Health and Preventive Medicine Monash University Monash Victoria Australia; ^5^ School of Public Health, the Chinese Academy of Medical Sciences and Peking Union Medical College Beijing China; ^6^ School of Public Health Fudan University Shanghai China; ^7^ Department of Global Health, School of Public Health Peking University Beijing China; ^8^ Department of Microbiology, Faculty of Medicine The Chinese University of Hong Kong Hong Kong SAR China; ^9^ Department of Otorhinolaryngology, Head and Neck Surgery, Faculty of Medicine The Chinese University of Hong Kong Hong Kong SAR China

**Keywords:** age‐standardized rate, average annual percentage change, incidence, temporal trend, tongue cancer

## Abstract

**Background:**

Tongue cancer is the most prevalent form of cancer in the intraoral region across many countries. This study aims to explore the global burden of the disease, its associated risk factors, and trends in incidence over time across different demographic groups.

**Methods:**

Data were extracted from the Global Cancer Observatory, Cancer Incidence in Five Continents Plus, Global Burden of Disease, the United Nations, and the World Bank. Linear regression analysis was applied to assess the relationship between tongue cancer incidence and various factors. Temporal trends in tongue cancer incidence across countries and regions were analyzed using the Average Annual Percentage Change (AAPC). The accuracy of these trend estimates was reported with 95% confidence intervals (CI).

**Results:**

A total of 151,338 cases of tongue cancer were identified globally, with an age‐standardized rate (ASR) of 1.7 per 100,000 population. The highest ASRs were observed in South‐Central Asia (3.4), Northern America (2.3), and Northern Europe (2.1). Males were found to have a higher ASR (2.6) compared to females (0.86). Tongue cancer incidence was significantly linked to a higher prevalence of smoking (*β* = 0.038, CI: 0.016–0.059, *p* = 0.001), alcohol consumption (*β* = 0.049, CI: 0.027–0.072, *p* < 0.001), and dietary factors (*β* = 0.013, CI: 0.002–0.024, *p* = 0.025). An increasing trend was presented globally based on pre‐2013 data, except for the Philippines, which showed the only significant drop.

**Conclusion:**

Geographical variation was observed in tongue cancer, with South‐Central Asia having the highest disease burden. The higher incidence of tongue cancer in males may be attributed to smoking and alcohol, highlighting the need for intensive lifestyle modifications.

**Abbreviations**
*β*
: beta coefficients
AAPC: average annual percentage change
ASR: age‐standardized rates
CI: confidence intervals
CI5 Plus: Five Continents Plus
GBD: global burden of disease
GDP: gross domestic product
GLOBOCAN: global cancer observatory
HDI: Human Development Index
HPV: human papillomavirus
ICD‐10: international classification of diseases, tenth revision
LMICs: low‐middle‐income countries
SD: standard deviation
SEER: surveillance, epidemiology, and end results
STROCSS: strengthening the reporting of cohort, cross‐sectional and case–control studies in surgery
WHO: World Health Organization

## 1 Background

Oral cavity malignancies are the most prevalent form of head and neck cancers, with tongue cancer accounting for a substantial proportion [1]. Globally, oral cancer is 2 to 3 times more prevalent in males than in females [2]. Data from the Japanese oral cancer registry indicate that the most commonly affected sites are the tongue (40.2%), gingiva (32.7%), buccal mucosa (10.1%), and floor of the mouth (9%) [3, 4]. In many countries, tongue cancer is the leading form of intraoral cancer, resulting in various health complications. This malignancy arises in the cells of the tongue and can be classified into two types: oral tongue cancer, which affects the visible portion, and base of tongue cancer, located at the back near the throat [5].

Surgery is a key treatment modality for tongue cancer, primarily aimed at removing the tumor and nearby lymph nodes to prevent the spread of cancer cells. Based on the clinical, pathological findings and the imaging techniques, the surgical planning on the portion of the tongue to be surgically removed [6]. From type I to type IV glossectomy, the classification clearly highlighted the structures of the involved part to facilitate the surgery [7]. Additionally, reconstructive surgery may be employed to restore the form and function of the tongue and surrounding areas posttreatment. The effectiveness of surgery in tongue cancer treatment is often enhanced when combined with other modalities such as radiation therapy or chemotherapy.

The local tumor control and survival rates for tongue cancer are relatively low. The 2‐year survival rate is 77% for patients diagnosed at an early stage (stage I‐II), but it decreases significantly to 52% for those with advanced‐stage disease (stage III‐IV) [8]. While the global incidence of oral cancer is slightly declining, the incidence of tongue cancer is on the rise [9]. It was highlighted that the rising trend was more significant in young female adults [10]. However, the driving mechanism behind these changes is yet to be determined. In the previous literature, a significant association was observed between tongue cancer and cigarette smoking and alcohol [11, 12, 13]. Research indicates that human papillomavirus (HPV) is a significant risk factor for oropharyngeal cancer and is frequently associated with base of tongue tumors (40%) compared to mobile tongue tumors (2.3%) [14, 15]. However, the rising trend of oral tongue cancer in the young population is unlikely to be significantly attributed to HPV [16].

The previous study used data sets limited to certain countries or specific groups of the population [10, 17], resulting in a lack of studies that include global populations. This study aims to provide a comprehensive analysis of the global disease burden, associated risk factors, and temporal incidence patterns of tongue cancer across demographic subgroups. By utilizing data from international cancer registries and risk factor databases, the findings will contribute to the development of primary preventive policies and strategies for early diagnosis.

## 2 Methods

### 2.1 Data Sources

The current study analyzed malignant neoplasms of all parts of the tongue. Data were sourced from the Global Cancer Observatory (GLOBOCAN), a global online database that compiles incidence and mortality rates for 26 cancer types. This database was utilized to estimate tongue cancer incidence across 185 countries in 2020 [18]. GLOBOCAN, developed by the International Association of Cancer Registries in collaboration with population‐based cancer registries, the World Health Organization (WHO), and freely available online, provides comprehensive data on cancer. The database incorporates cancer incidence and mortality data, the incidence‐to‐mortality ratio, trend predictions, and estimates for neighboring countries using global and national cancer registry data. Additionally, the Cancer Incidence in Five Continents Plus (CI5 Plus) database, which includes 10 years of cancer incidence data (2003–2012) from 108 countries, was used to analyze tongue cancer (C01 and C02) proportions by site, as classified by the International Classification of Diseases, Tenth Revision (ICD‐10) [19].

The most up‐to‐date and historical incidence dataset from the CI5 Plus database is categorized by year, population subgroup, and geographical region. This dataset is commonly used to study time trends and to interpret differences in cancer incidence over time. The database of Global Burden of Diseases (GBD) version 2019 was used to analyze the risk factors of particular nations, including the prevalence of smoking, alcohol drinking, physical inactivity, unhealthy diet, obesity, hypertension, diabetes, and lipid disorders [20]. Alcohol drinking was referred to as the prevalence of relative risk of alcohol drinking [21]. Unhealthy diet referred to the intake of any processed meat [22], red meat [23], high sodium intake [24], high sugar‐sweetened beverages intake [25], diet high in trans fatty acids [26], diet low in calcium [27], fiber [28], low fruit intake [29], diet low in legumes [30], milk [31], nuts and seeds [32], omega‐6 polyunsaturated fatty acids [33], seafood omega‐3 fatty acids [34], vegetables [35], and low whole grain intake [36]. The database offers a thorough overview of mortality and disability across countries, periods, age groups, and sex. It aims to enhance healthcare systems and strengthen health equity by computing health loss brought by particular illnesses, accidents, and risk factors. More than 7000 researchers from 156 countries and regions congregated and determined the data on the early mortality and disability of over 350 traumas and diseases. The United Nations (UN) and the World Bank provided the gross domestic product (GDP) per capita and human development index (HDI) for each countries [37, 38]. Furthermore, this study adheres to the Strengthening the Reporting of Cohort, Cross‐Sectional and Case–Control Studies in Surgery (STROCSS) criteria. The corresponding checklist has been satisfactorily filled out and can be provided upon request [39].

To facilitate meaningful comparisons between countries, we employed several methodological approaches. First, all incidence estimates were age‐standardized using the World Standard Population to account for demographic differences. Second, comparisons were conducted exclusively within the same database, ensuring consistency in study periods and methodologies. For example, the assessment of disease burden across different countries utilized data from GLOBOCAN, while risk factors were analyzed using the GBD database, and trends were examined through CI5 Plus. This study utilized country‐level data extracted from GLOBOCAN and the GBD database to investigate the ecological associations between factors and the incidence of tongue cancer; the inference of individual‐level risk relationships or establishing causality inference is not available due to the data source constraints. This rigorous approach enhances the reliability and validity of our comparisons.

### 2.2 Calculation

#### 2.2.1 Statistical Analysis

For the ecological analysis, linear regression was employed to examine the relationship between tongue cancer incidence and various risk factors at the country level, stratified by sex and age. The analyzed risk factors included HDI, GDP per capita, and the prevalence of smoking, alcohol consumption, physical inactivity, unhealthy diets, obesity, hypertension, diabetes, and lipid disorders. We employed various strategies to control for key confounding variables, namely age and sex, in our analysis. First, the outcome variable of incidence was age‐standardized using the WHO standard population, ensuring comparability across different countries. Secondly, separate analyses were conducted for both sexes (male and female) and different age categories (young population: 15–49 years, and older population: 50–74 years). From the regression analysis, Beta coefficients (*β*) and 95% confidence intervals (CI) were calculated. The Beta coefficient (*β* estimate) represents the amount of change in the outcome variable (age‐standardized incidence rate, ASR) for each unit increase in a predictor variable (risk factor). A *p* value of less than 0.05 was considered statistically significant, and all CIs are shown for the 95% value.

Joinpoint regression was chosen for trend analysis because it can objectively identify changes in trends over time [40]. This approach allows estimating changes in the rate of change, providing more nuanced insights than simpler linear models. The flexibility of joinpoint regression is advantageous compared to methods requiring pre‐specified trend changes, which could introduce bias. Joinpoint regression analysis program was used to carry out the trend analysis (Data S1). The software was created by the Surveillance, Epidemiology, and End Results (SEER) Program of the National Cancer Institute of the United States. AAPC was used to determine the temporal trend of tongue cancer incidence by country and region [41]. As is customary in cancer epidemiology studies, the most recent 10 years of data were used for the analysis. The incidence data were transformed logarithmically, and standard errors were calculated. The Average Annual Percent Change (AAPC) and 95% confidence intervals (CI) were then analyzed across various populations and categories. Temporal trends in tongue cancer were represented using the AAPC, where a positive AAPC indicated an increasing trend, and a negative AAPC indicated a decreasing trend. The 95% confidence intervals were used to assess the accuracy of the trend estimations. For instance, a range that overlaps with 0 indicates a continuous trend without an obvious increase or decrease. Furthermore, the incidence rate of tongue cancer was determined by age (all population: 0–85 or above, young population: 15–49, old population: 50–74), sex (male and female), and geographic region (America, Europe, Asia, Oceania, and Africa). A maximum of 1 join point was allowed. The model selection is weighted BIC. Joinpoint version 5.3.0.0 was used for the trend analysis.

## 3 Results

### 3.1 Tongue Cancer Incidence in 2020

**FIGURE 1:**
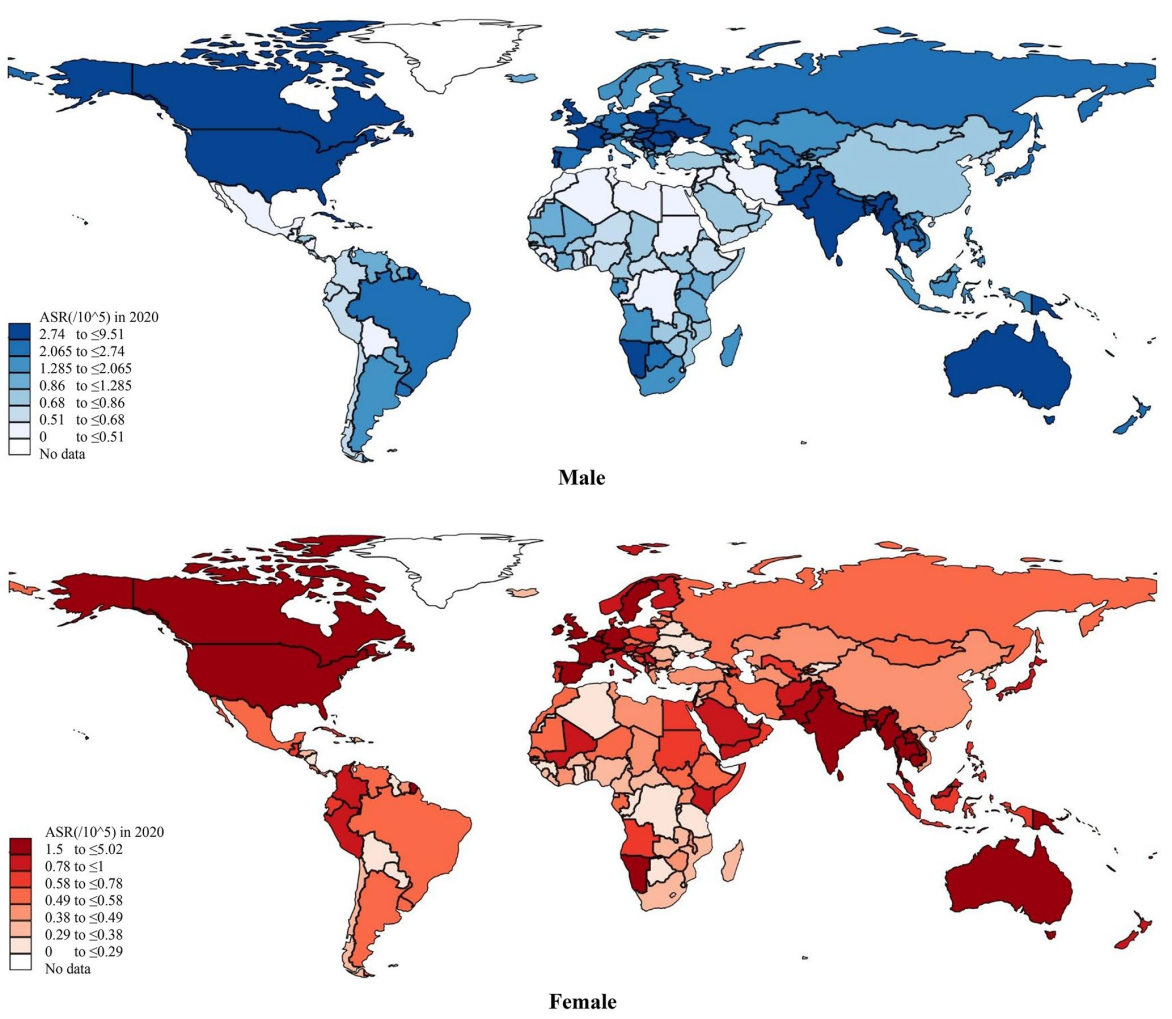
Global incidence of tongue cancer by sex, all ages, in 2020.

There were in total 151,338 cases of tongue cancer globally, with an ASR of 1.7 per 100,000 persons (Figure 1). The highest ASRs were reported in South‐Central Asia (3.4), Northern America (2.3), and Northern Europe (2.1); the lowest ASRs were reported in Northern Africa (0.4), Sub‐Saharan Africa (0.63), and Western Asia (0.65). There was an over eight‐fold variation across regions. As for countries, the highest ASRs were found in Papua New Guinea (7.1), Pakistan (3.9), India (3.8), Sri Lanka (3.7), and Bangladesh (3.6). The lowest ASRs were found in Brunei Darussalam (0.1), the Republic of the Gambia (0.14), and Algeria (0.18).

### 3.2 Tongue Cancer Incidence by Subgroup in 2020

**FIGURE 2:**
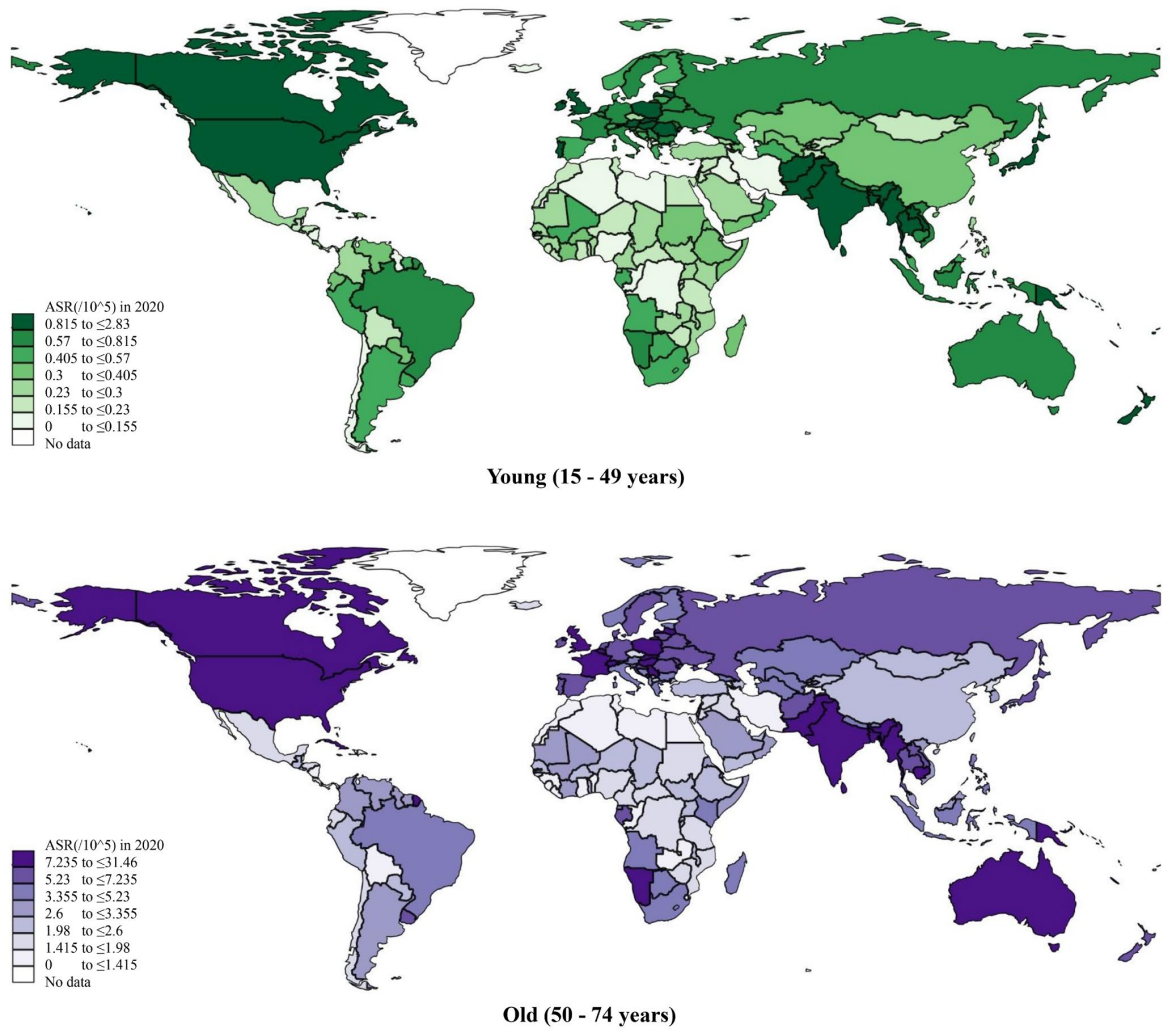
Global incidence of tongue cancer by age, both sexes, in 2020.

For the male population, a total of 109,432 new cases of tongue cancer were reported globally, with an age‐standardized incidence rate (ASR) of 2.6 per 100,000 persons. A much lower ASR was reported in females; there were 41,906 new cases and the ASR was 0.86. The greatest ASRs were found in South‐Central Asia (5.5 in males vs. 1.4 in females), Northern America (3.5 vs. 1.3), Central and Eastern Europe (3.0 vs. 0.51), and Northern Europe (2.9 vs. 1.3). As for countries, the greatest ASRs were found in Papua New Guinea (9.5 vs. 5.0), Sri Lanka (6.8 vs. 1.2), and India (6.2 vs. 1.4); whilst the lowest ASRs were found in Algeria (0.14 vs. 0.21), Brunei Darussalam (0.15 vs. 0.05), and the Republic of the Gambia (0.15 vs. 0.12). Detailed results can be referred to in Figure 1 and Table 1a. Turning to young and old populations, a higher ASR was found in the old population (ASR: 5.8, new cases: 90,714), compared with the young population (ASR: 1.0, new cases: 39,511). The greatest ASRs were found in South‐Central Asia (11.0 in males vs. 2.3 in females), Northern America (9.8 vs. 0.99), Northern Europe (8.5 vs. 0.92), and Western Europe (7.8 vs. 0.82). As for countries, the greatest ASRs were found in Papua New Guinea (31.5 vs. 2.0), Sri Lanka (13.4 vs. 1.8), and Bangladesh (13.0 vs. 2.0). The lowest ASRs were found in Algeria (0.47 vs. 0.13), Jamaica (0.53 vs. 0.13), and the Republic of Congo (0.78 vs. 0.06). Detailed results can be referred to in Figure 2 and Table 1b. Concerning the global incidence of tongue cancer by HDI, countries with very high HDI have the highest ASR (1.4), followed by medium (0.84), high (0.73), and low (0.56) in both sexes. The same trend was observable in males only, females only, young populations, and old populations respectively (Table S1).

**TABLE 1a:** Global incidence of tongue cancer by sex, all ages, in 2020.

Region	Both sexes		Male		Female	
	New cases	ASR	New cases	ASR	New cases	ASR
World	151,338	1.7	109,432	2.6	41,906	0.86
Asia	98,640	1.8	73,734	2.8	24,906	0.87
Eastern Asia	20,286	0.91	12,751	1.3	7535	0.54
China	13,694	0.59	8394	0.77	5300	0.46
Japan	5524	1.7	3696	2.5	1828	0.91
Korea, Democratic Republic of	149	0.41	97	0.59	52	0.24
Korea, Republic of	902	0.97	556	1.2	346	0.65
Mongolia	17	0.67	8	0.69	9	0.58
South‐Eastern Asia	8919	1.2	5980	1.8	2939	0.75
Brunei Darussalam	0	0.10	0	0.15	0	0.05
Cambodia	238	1.9	132	2.4	106	1.4
Indonesia	2821	0.98	1889	1.4	932	0.63
Lao People's Democratic Republic	99	1.9	60	2.2	39	1.5
Malaysia	329	0.97	189	1.1	140	0.85
Myanmar	1203	2.2	881	3.7	322	1.1
Philippines	908	0.99	538	1.3	370	0.70
Singapore	114	1.1	81	1.5	33	0.67
Thailand	2120	1.8	1393	2.7	727	1.0
Timor‐Leste	5	0.59	2	0.53	3	0.54
Viet Nam	1082	0.96	815	1.5	267	0.42
South‐Central Asia	67,856	3.4	54,157	5.5	13,699	1.4
Afghanistan	349	1.8	257	2.7	92	0.91
Bangladesh	5330	3.6	3883	5.1	1447	2.0
Bhutan	8	1.1	6	1.5	2	0.66
India	53,263	3.8	43,507	6.2	9756	1.4
Iran, Islamic Republic of	300	0.36	84	0.19	216	0.53
Kazakhstan	221	1.0	168	1.9	53	0.41
Kyrgyzstan	36	0.69	28	1.3	8	0.26
Maldives	2	0.46	2	0.95	0	0.00
Nepal	326	1.3	252	2.3	74	0.53
Pakistan	6469	3.9	4730	5.5	1739	2.2
Sri Lanka	1107	3.7	914	6.8	193	1.2
Tajikistan	29	0.54	17	0.71	12	0.34
Turkmenistan	63	1.2	50	2.3	13	0.47
Uzbekistan	353	1.3	259	2.2	94	0.59
Western Asia	1579	0.65	846	0.74	733	0.60
Armenia	16	0.37	13	0.67	3	0.12
Azerbaijan	79	0.69	38	0.77	41	0.64
Bahrain	8	0.78	5	0.67	3	0.66
Gaza Strip and West Bank	14	0.46	8	0.51	6	0.41
Georgia	67	1.0	52	1.8	15	0.33
Iraq	106	0.42	42	0.40	64	0.51
Israel	98	0.74	39	0.69	59	0.87
Jordan	33	0.43	19	0.53	14	0.37
Kuwait	33	0.85	23	0.91	10	0.78
Lebanon	33	0.42	18	0.47	15	0.38
Oman	25	0.73	18	0.74	7	0.60
Qatar	16	1.3	13	1.1	3	1.4
Saudi Arabia	209	0.79	116	0.73	93	0.84
Syrian Arab Republic	62	0.46	31	0.51	31	0.43
Turkey	636	0.63	354	0.78	282	0.49
United Arab Emirates	31	0.57	18	0.51	13	0.83
Yemen	113	0.65	39	0.54	74	0.83
Oceania	1457	2.5	945	3.3	512	1.7
Australia	872	2.0	576	2.8	296	1.2
Fiji	9	0.98	5	1.2	4	0.85
France, New Caledonia	6	1.7	4	2.3	2	1.1
French Polynesia	4	1.0	3	1.4	1	0.61
Guam	3	0.98	2	1.6	1	0.44
New Zealand	140	1.7	97	2.6	43	0.95
Sir	417	7.1	254	9.5	163	5.0
Samoa	1	1.0	1	1.7	0	0.30
Solomon Islands	3	0.81	2	0.97	1	0.61
Vanuatu	2	0.84	1	0.84	1	0.88
Northern America	15,240	2.3	10,829	3.5	4411	1.3
Canada	1472	2.1	1062	3.1	410	1.0
United States of America	13,768	2.4	9767	3.6	4001	1.3
Latin America and the Caribbean	7192	1.1	4693	1.5	2499	0.65
Central America & Caribbean	1234	0.72	613	0.91	621	0.57
Bahamas	2	0.48	2	1.1	0	0.00
Barbados	4	0.67	3	1.2	1	0.22
Belize	0	0.00	0	0.00	0	0.00
Costa Rica	38	0.57	21	0.67	17	0.44
Cuba	17	0.86	11	1.3	6	0.47
Dominican Republic	62	0.53	37	0.67	25	0.37
El Salvador	52	0.67	24	0.72	28	0.62
France, Guadeloupe	12	1.4	9	2.6	3	0.44
France, Martinique	7	0.97	6	1.8	1	0.33
Guatemala	78	0.62	28	0.47	50	0.71
Haiti	62	0.67	47	1.1	15	0.28
Honduras	42	0.53	27	0.77	15	0.31
Jamaica	7	0.23	4	0.25	3	0.17
Mexico	716	0.53	303	0.47	413	0.52
Nicaragua	14	0.24	9	0.35	5	0.15
Panama	24	0.45	13	0.53	11	0.39
Puerto Rico	77	1.2	56	2.1	21	0.56
Saint Lucia	4	2.0	4	3.8	0	0.33
Trinidad and Tobago	16	0.81	9	0.91	7	0.71
South America	5958	1.2	4080	1.8	1878	0.65
Argentina	530	0.93	351	1.4	179	0.52
Bolivia, Plurinational State of	33	0.29	22	0.42	11	0.15
Brazil	3930	1.4	3011	2.5	919	0.57
Chile	122	0.41	71	0.53	51	0.33
Colombia	496	0.76	183	0.65	313	0.87
Ecuador	135	0.70	62	0.67	73	0.72
French Guiana	1	0.39	1	0.76	0	0.00
Guyana	5	0.60	5	1.2	0	0.06
Paraguay	44	0.69	39	1.2	5	0.11
Peru	305	0.77	103	0.55	202	0.99
Suriname	5	0.83	4	1.3	1	0.42
Uruguay	75	1.3	53	2.2	22	0.57
Venezuela, Bolivarian Republic of	277	0.87	175	1.2	102	0.55
Europe	24,181	1.7	16,535	2.7	7646	0.85
Northern Europe	4128	2.1	2667	2.9	1461	1.3
Denmark	154	1.4	96	1.7	58	0.98
Estonia	32	1.2	21	1.9	11	0.63
Finland	177	1.4	103	1.8	74	0.93
Iceland	4	0.63	3	1.1	1	0.31
Ireland	145	1.8	93	2.5	52	1.2
Latvia	90	2.4	77	5.2	13	0.48
Lithuania	67	1.3	51	2.3	16	0.46
Norway	132	1.2	84	1.6	48	0.80
Sweden	311	1.4	171	1.6	140	1.2
United Kingdom	3016	2.4	1968	3.4	1048	1.5
Western Europe	7322	1.9	4721	2.6	2601	1.2
Austria	235	1.4	160	2.0	75	0.80
Belgium	420	2.0	278	2.7	142	1.3
France	2756	2.3	1814	3.2	942	1.4
Germany	2994	1.8	1885	2.4	1109	1.1
Luxembourg	19	1.7	15	2.8	4	0.65
Switzerland	336	1.9	237	2.9	99	0.93
The Netherlands	562	1.6	332	2.0	230	1.2
Southern Europe	5345	1.4	3426	2.0	1919	0.80
Albania	40	0.69	22	0.81	18	0.61
Bosnia and Herzegovina	76	1.2	54	1.8	22	0.61
Croatia	410	2.0	325	3.4	85	0.71
Cyprus	387	1.9	259	2.71	128	1.1
Greece	231	0.92	154	1.5	77	0.46
Italy	1661	1.2	994	1.5	667	0.80
Malta	9	0.70	4	0.73	5	0.68
Montenegro	19	1.7	13	2.6	6	0.80
North Macedonia	26	0.64	17	0.92	9	0.39
Portugal	423	2.0	309	3.5	114	0.72
Serbia	331	1.9	247	3.0	84	0.91
Slovenia	49	1.2	38	2.0	11	0.48
Spain	1683	1.5	990	2.1	693	1.0
Central and Eastern Europe	7386	1.6	5721	3.0	1665	0.51
Belarus	209	1.3	174	2.6	35	0.29
Bulgaria	128	0.94	93	1.5	35	0.43
Czechia	76	0.56	41	0.55	35	0.54
Hungary	356	2.0	259	3.2	97	0.89
Poland	1306	1.8	943	3.0	363	0.78
Republic of Moldova	89	1.4	80	3.0	9	0.20
Romania	613	1.7	526	3.3	87	0.35
Russian Federation	3358	1.4	2534	2.5	824	0.53
Slovakia	241	2.5	207	4.9	34	0.58
Ukraine	1010	1.3	864	2.77	146	0.27
Africa	4628	0.60	2696	0.68	1932	0.48
Northern Africa	884	0.40	280	0.28	604	0.53
Algeria	73	0.18	30	0.14	43	0.21
Egypt	388	0.43	98	0.24	290	0.65
Libya	17	0.29	5	0.20	12	0.41
Morocco	189	0.48	73	0.37	116	0.53
Sudan	154	0.56	50	0.37	104	0.73
Tunisia	63	0.45	24	0.36	39	0.49
Sub‐Saharan Africa	3744	0.63	2416	0.85	1328	0.48
Angola	170	1.2	117	1.7	53	0.67
Benin	25	0.33	12	0.28	13	0.35
Botswana	22	1.2	19	2.6	3	0.25
Burkina Faso	63	0.60	46	0.74	17	0.38
Burundi	33	0.56	23	0.88	10	0.31
Cabo Verde	10	2.1	7	2.9	3	1.2
Cameroon	80	0.53	51	0.70	29	0.38
Central African Republic	12	0.49	7	0.59	5	0.38
Chad	41	0.56	24	0.70	17	0.41
Comoros	2	0.35	2	0.77	0	0.00
Congo, Democratic Republic of	169	0.35	104	0.48	65	0.26
Congo, Republic of	6	0.18	3	0.19	3	0.17
Côte d'Ivoire	120	1.5	92	2.5	28	0.54
Djibouti	5	0.63	3	0.63	2	0.57
Equatorial Guinea	6	0.81	5	1.2	1	0.35
Eritrea	15	0.67	8	0.74	7	0.57
Eswatini	2	0.35	1	0.44	1	0.23
Ethiopia	433	0.63	223	0.63	210	0.57
France, La Réunion	23	1.6	19	2.9	4	0.45
Gabon	17	1.3	14	1.9	3	0.57
Ghana	68	0.33	50	0.52	18	0.16
Guinea	23	0.33	13	0.48	10	0.24
Guinea‐Bissau	4	0.42	2	0.52	2	0.35
Kenya	277	1.0	154	1.1	123	0.90
Lesotho	10	0.63	7	1.0	3	0.31
Liberia	13	0.42	7	0.48	6	0.38
Madagascar	147	0.91	118	1.5	29	0.35
Malawi	64	0.60	38	0.74	26	0.48
Mali	89	0.91	39	0.88	50	0.92
Mauritania	18	0.70	10	0.88	8	0.54
Mauritius	21	1.1	13	1.4	8	0.73
Mozambique	91	0.53	60	0.81	31	0.32
Namibia	36	2.3	24	3.8	12	1.3
Niger	56	0.56	27	0.59	29	0.54
Nigeria	449	0.42	276	0.55	173	0.31
Rwanda	35	0.46	25	0.70	10	0.24
Sao Tome and Principe	0	0.00	0	0.00	0	0.00
Senegal	60	0.67	30	0.77	30	0.57
Sierra Leone	19	0.42	11	0.55	8	0.31
Somalia	60	0.70	32	0.74	28	0.67
South Africa	511	1.1	415	1.9	96	0.34
South Sudan	41	0.67	24	0.77	17	0.51
Tanzania, United Republic of	169	0.56	116	0.88	53	0.29
The Republic of the Gambia	2	0.14	1	0.15	1	0.12
Togo	23	0.49	14	0.63	9	0.38
Uganda	121	0.72	84	0.96	37	0.39
Zambia	43	0.53	27	0.74	16	0.32
Zimbabwe	40	0.57	19	0.72	21	0.45

*Note:* ASR: per 100,000 persons.

**TABLE 1b:** Global incidence of tongue cancer by age, both sexes, in 2020.

Region	Young		Old	
	New cases	ASR	New cases	ASR
World	39,511	1.0	90,714	5.8
Asia	31,467	1.3	55,258	5.9
Eastern Asia	3682	0.57	12,953	3.1
China	2663	0.31	9452	2.3
Japan	769	1.2	2893	6.3
Korea, Democratic Republic of	31	0.19	99	1.6
Korea, Republic of	215	0.71	498	2.9
Mongolia	4	0.20	11	2.6
South‐Eastern Asia	2317	0.60	5711	4.5
Brunei Darussalam	0	0.00	0	1.65
Cambodia	48	0.66	168	7.2
Indonesia	894	0.58	1889	3.8
Lao People's Democratic Republic	32	0.96	61	6.9
Malaysia	87	0.50	179	3.0
Myanmar	378	1.3	743	7.5
Philippines	161	0.30	592	3.7
Singapore	23	0.60	79	4.04
Thailand	360	0.89	1346	6.6
Timor‐Leste	0	0.00	4	3.0
Viet Nam	334	0.59	650	3.3
South‐Central Asia	25,108	2.3	35,663	11.0
Afghanistan	138	0.97	181	5.9
Bangladesh	1770	2.0	3048	13.0
Bhutan	2	0.53	4	3.8
India	19,867	2.7	27,633	11.7
Iran, Islamic Republic of	78	0.15	141	0.97
Kazakhstan	30	0.32	151	4.0
Kyrgyzstan	6	0.21	24	2.5
Maldives	1	0.31	1	1.8
Nepal	102	0.77	202	4.8
Pakistan	2819	2.8	3281	12.5
Sri Lanka	211	1.8	721	13.4
Tajikistan	9	0.22	13	1.3
Turkmenistan	17	0.57	40	4.6
Uzbekistan	58	0.33	223	4.6
Western Asia	360	0.26	931	2.4
Armenia	1	0.05	14	1.7
Azerbaijan	18	0.31	44	2.2
Bahrain	0	0.02	8	4.3
Gaza Strip and West Bank	4	0.19	8	1.7
Georgia	5	0.25	51	4.3
Iraq	25	0.14	59	1.6
Israel	14	0.33	51	2.7
Jordan	12	0.23	16	1.4
Kuwait	13	0.33	19	3.0
Lebanon	5	0.14	20	1.5
Oman	13	0.41	13	2.8
Qatar	2	0.11	6	3.1
Saudi Arabia	64	0.26	119	2.9
Syrian Arab Republic	15	0.17	37	1.7
Turkey	122	0.26	390	2.3
United Arab Emirates	10	0.13	20	2.2
Yemen	37	0.36	56	2.2
Oceania	208	0.97	1038	10.5
Australia	98	0.73	608	8.5
Fiji	3	0.79	5	2.9
France, New Caledonia	0	0.18	5	7.7
French Polynesia	0	0.18	3	4.8
Guam	0	0.00	2	4.5
New Zealand	23	0.95	90	6.5
Papua New Guinea	81	2.0	322	31.5
Samoa	0	0.00	1	5.2
Solomon Islands	2	0.55	1	2.2
Vanuatu	1	0.40	1	2.6
Northern America	1791	0.99	10,900	9.8
Canada	180	0.94	1068	8.7
United States of America	1611	0.99	9832	9.9
Latin America and the Caribbean	1486	0.47	4178	3.8
Central America & Caribbean	280	0.31	661	2.6
Bahamas	0	0.00	2	2.8
Barbados	0	0.00	2	2.7
Belize	0	0.00	0	0.00
Costa Rica	7	0.24	22	2.1
Cuba	3	0.40	9	2.9
Dominican Republic	22	0.40	25	1.4
El Salvador	12	0.35	19	1.7
France, Guadeloupe	2	0.72	7	5.3
France, Martinique	1	0.66	5	3.5
Guatemala	14	0.18	44	2.3
Haiti	28	0.52	21	1.4
Honduras	8	0.16	18	1.6
Jamaica	2	0.13	3	0.53
Mexico	163	0.24	402	1.7
Nicaragua	3	0.09	8	0.92
Panama	4	0.17	11	1.4
Puerto Rico	8	0.49	47	4.7
Saint Lucia	1	0.53	4	10.1
Trinidad and Tobago	2	0.27	12	3.4
South America	1206	0.54	3517	4.3
Argentina	102	0.46	302	3.2
Bolivia, Plurinational State of	10	0.18	19	0.99
Brazil	809	0.68	2395	5.2
Chile	13	0.13	72	1.5
Colombia	71	0.26	277	2.7
Ecuador	37	0.40	51	1.7
French Guiana	0	0.11	1	1.8
Guyana	0	0.00	4	3.1
Paraguay	14	0.40	25	2.3
Peru	89	0.49	154	2.5
Suriname	1	0.49	3	2.6
Uruguay	8	0.45	46	5.3
Venezuela, Bolivarian Republic of	52	0.36	168	3.0
Europe	3230	0.79	16,645	7.0
Northern Europe	508	0.92	2771	8.5
Denmark	13	0.42	116	6.1
Estonia	2	0.26	21	5.2
Finland	15	0.54	106	5.2
Iceland	0	0.00	2	1.9
Ireland	24	0.82	93	7.0
Latvia	11	1.0	63	10.3
Lithuania	6	0.44	52	5.7
Norway	13	0.46	86	5.1
Sweden	30	0.60	173	5.3
United Kingdom	394	1.1	2059	10.1
Western Europe	769	0.82	5102	7.8
Austria	40	0.83	151	5.2
Belgium	39	0.62	315	8.7
France	278	0.81	1972	9.6
Germany	323	0.75	2051	7.2
Luxembourg	2	0.62	13	7.6
Switzerland	21	0.46	227	8.1
The Netherlands	66	0.77	373	6.3
Southern Europe	643	0.60	3231	5.4
Albania	1	0.11	24	2.7
Bosnia and Herzegovina	10	0.55	55	4.8
Croatia	61	0.93	307	8.6
Cyprus	63	0.94	245	7.1
Greece	27	0.45	124	3.6
Italy	205	0.58	915	4.3
Malta	0	0.00	6	3.3
Montenegro	1	0.31	15	7.9
North Macedonia	0	0.00	15	2.4
Portugal	87	1.4	245	7.2
Serbia	32	0.64	227	7.7
Slovenia	8	0.63	33	4.8
Spain	148	0.48	1020	6.8
Central and Eastern Europe	1310	0.82	5541	6.8
Belarus	34	0.63	160	5.5
Bulgaria	28	0.68	91	3.8
Czechia	26	0.26	44	2.1
Hungary	52	0.82	280	9.1
Poland	205	0.91	950	7.7
Republic of Moldova	14	0.58	69	5.9
Romania	106	0.89	438	7.1
Russian Federation	624	0.76	2567	5.8
Slovakia	45	1.3	180	10.7
Ukraine	176	0.68	762	5.6
Africa	1329	0.25	2695	2.1
Northern Africa	250	0.22	380	1.0
Algeria	30	0.13	33	0.47
Egypt	103	0.22	141	0.96
Libya	3	0.08	9	1.0
Morocco	40	0.22	98	1.4
Sudan	60	0.34	71	1.6
Tunisia	14	0.23	28	1.1
Sub‐Saharan Africa	1079	0.26	2315	2.4
Angola	51	0.46	100	4.2
Benin	14	0.25	12	1.2
Botswana	6	0.53	13	4.6
Burkina Faso	30	0.39	32	2.2
Burundi	8	0.19	19	2.2
Cabo Verde	4	1.5	4	5.0
Cameroon	31	0.30	44	2.1
Central African Republic	4	0.22	8	1.9
Chad	13	0.25	24	2.1
Comoros	0	0.13	1	1.6
Congo, Democratic Republic of	46	0.15	105	1.4
Congo, Republic of	1	0.06	4	0.78
Côte d'Ivoire	13	0.57	84	6.0
Djibouti	1	0.29	4	2.7
Equatorial Guinea	2	0.39	4	3.5
Eritrea	4	0.31	9	2.6
Eswatini	1	0.26	1	1.0
Ethiopia	158	0.33	242	2.3
France, La Réunion	2	0.53	16	6.5
Gabon	5	0.50	12	5.3
Ghana	22	0.17	43	1.3
Guinea	11	0.24	10	0.88
Guinea‐Bissau	1	0.23	3	1.8
Kenya	66	0.30	174	3.9
Lesotho	3	0.33	6	2.2
Liberia	4	0.22	6	1.4
Madagascar	38	0.36	89	3.5
Malawi	20	0.28	32	2.3
Mali	32	0.43	41	2.9
Mauritania	5	0.25	12	2.8
Mauritius	2	0.29	16	4.4
Mozambique	32	0.29	46	1.9
Namibia	9	0.78	21	8.2
Niger	13	0.20	38	2.1
Nigeria	105	0.15	304	1.7
Rwanda	7	0.14	21	1.7
Sao Tome and Principe	0	0.00	0	0.00
Senegal	20	0.28	34	2.4
Sierra Leone	7	0.22	8	1.2
Somalia	18	0.33	36	2.8
South Africa	134	0.46	404	4.6
South Sudan	11	0.26	25	2.5
Tanzania, United Republic of	43	0.16	88	1.9
The Republic of the Gambia	0	0.00	0	0.00
Togo	8	0.21	14	2.1
Uganda	41	0.26	72	2.5
Zambia	20	0.30	16	1.3
Zimbabwe	13	0.22	18	1.6

*Note:* ASR: per 100,000 persons.

### 3.3 Associations of Risk Factors With Tongue Cancer Incidence

In terms of all sexes and ages, the tongue cancer was associated with higher HDI (*β* = 0.111, CI 0.031 to 0.192, *p* = 0.007; Table 2), higher GDP per capita (*β* = 0.076, CI 0.013 to 0.138, *p* = 0.017), higher prevalence of smoking (*β* = 0.038, CI 0.016 to 0.059, *p* = 0.001), alcohol drinking (*β* = 0.049, CI 0.027 to 0.072, *p* < 0.001), dietary (*β* = 0.013, CI 0.002 to 0.024, *p* = 0.025), diabetes (*β* = 0.033, CI 0.011 to 0.056, *p* = 0.004) and lipid disorder (*β* = 0.016, CI 0.005 to 0.026, *p* = 0.003). No significant association could be found between tongue cancer and physical inactivity, obesity, and hypertension.

**TABLE 2:** Log transformation results of risk factors with tongue cancer.

Outcome	Risk factor	Overall			
		*β*	*95% CI*		*P*
	HDI	0.129	0.066	0.192	< 0.001*
	GDP per capita	0.088	0.039	0.136	< 0.001*
	Smoking	0.043	0.026	0.060	< 0.001*
	Alcohol drinking	0.061	0.044	0.078	< 0.001*
All Sexes and ages	Dietary	0.013	0.004	0.022	0.006*
	Physical inactivity	0.006	−0.026	0.038	0.718
	Obesity	0.004	−0.005	0.014	0.337
	Hypertension	0.014	0.003	0.026	0.015*
	Diabetes	0.026	0.007	0.044	0.006*
	Lipid	0.018	0.010	0.027	< 0.001*
	HDI	0.148	0.071	0.225	< 0.001*
	GDP per capita	0.102	0.044	0.161	< 0.001*
	Smoking	0.029	0.014	0.044	< 0.001*
	Alcohol drinking	0.062	0.048	0.075	< 0.001*
Male	Dietary	0.017	0.009	0.025	< 0.001*
	Physical inactivity	−0.039	−0.077	0.000	0.048*
	Obesity	0.004	−0.007	0.015	0.438
	Hypertension	0.023	0.010	0.036	< 0.001*
	Diabetes	0.025	0.004	0.047	0.018*
	Lipid	0.020	0.011	0.030	< 0.001*
	HDI	0.092	0.030	0.154	0.004*
	GDP per capita	0.077	0.030	0.124	0.002*
	Smoking	0.036	0.018	0.054	< 0.001*
	Alcohol drinking	0.034	0.008	0.059	0.010*
Female	Dietary	0.001	−0.009	0.012	0.819
	Physical inactivity	0.037	0.009	0.066	0.010*
	Obesity	0.000	−0.009	0.008	0.979
	Hypertension	−0.003	−0.014	0.008	0.570
	Diabetes	0.013	−0.005	0.032	0.158
	Lipid	0.013	0.005	0.021	0.001*
	HDI	0.108	0.033	0.182	0.005*
	GDP per capita	0.102	0.043	0.162	< 0.001*
	Smoking	0.036	0.014	0.059	0.002*
	Alcohol drinking	0.053	0.033	0.072	< 0.001*
Young	Dietary	0.020	0.010	0.030	< 0.001*
	Physical inactivity	−0.026	−0.065	0.013	0.185
	Obesity	−0.010	−0.021	0.001	0.062
	Hypertension	−0.012	−0.032	0.008	0.226
	Diabetes	0.033	−0.019	0.086	0.213
	Lipid	0.017	0.005	0.028	0.004*
	HDI	0.134	0.064	0.205	< 0.001*
	GDP per capita	0.099	0.045	0.152	< 0.001*
	Smoking	0.043	0.027	0.058	< 0.001*
	Alcohol drinking	0.068	0.048	0.087	< 0.001*
Old	Dietary	0.012	0.003	0.020	0.009*
	Physical inactivity	−0.007	−0.041	0.027	0.684
	Obesity	0.002	−0.006	0.010	0.696
	Hypertension	0.001	−0.010	0.011	0.891
	Diabetes	−0.005	−0.016	0.007	0.419
	Lipid	0.018	0.009	0.027	< 0.001*

*Note:* The analysis was conducted using univariable linear regression model at a country level. The beta coefficient can be interpreted as the change in incidence or mortality associated with 1% increase of a certain risk factor. Bold values: *p < 0.05.

Abbreviations: ASR, age‐standardized rate; CI, confidence interval; GDP, gross domestic products; HDI, human development index; *β*, beta coefficient.

### 3.4 Associations of Risk Factors With Tongue Cancer Incidence by Subtype

**FIGURE 3:**
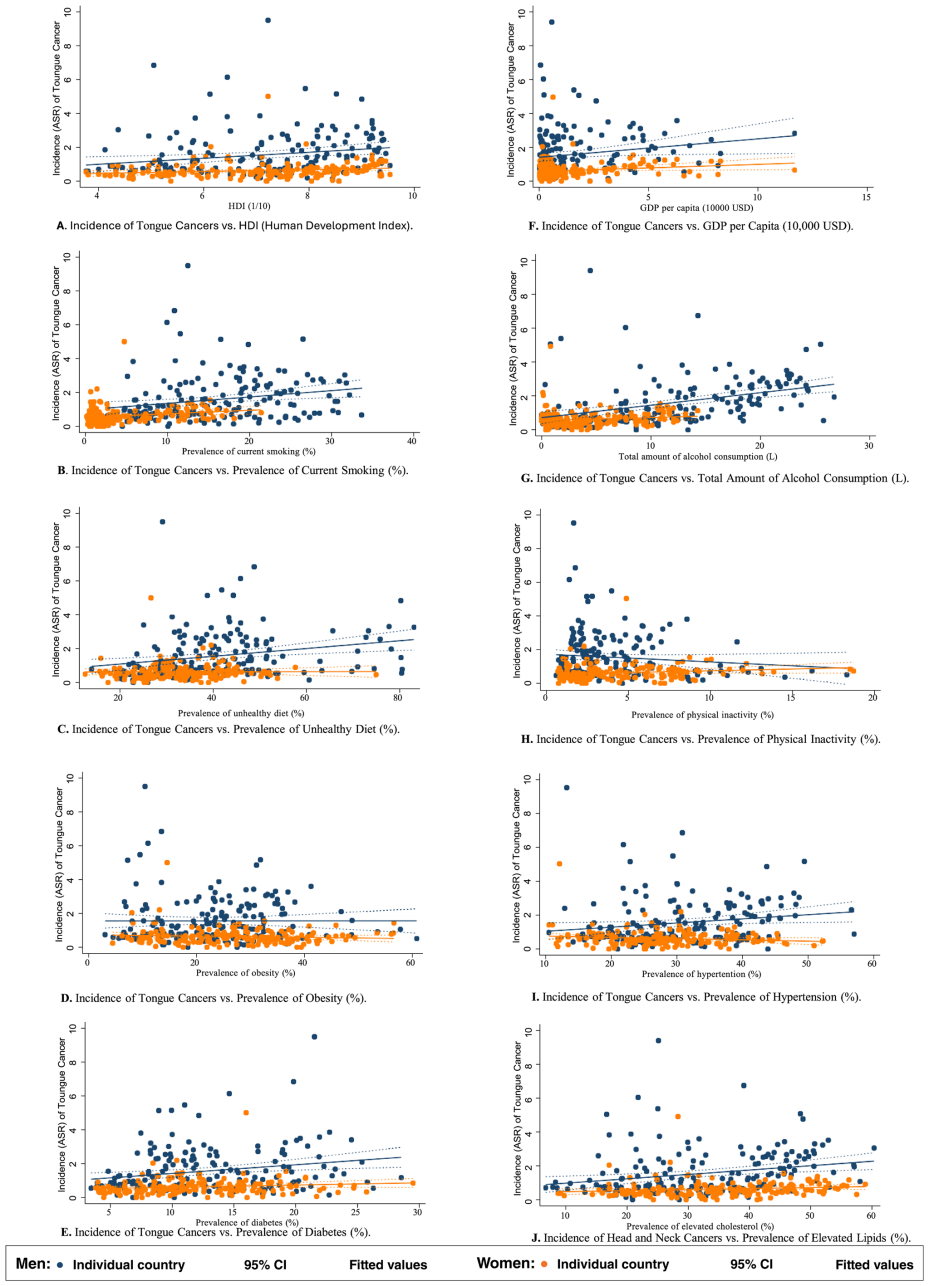
Associations between risk factors and tongue cancer by sex.

In the male population, tongue cancer was associated with higher HDI (*β* = 0.178, CI 0.059 to 0.308, *p* = 0.008), higher GDP per capita (*β* = 0.109, CI 0.009 to 0.210, *p* = 0.034), higher prevalence of smoking (*β* = 0.038, CI 0.012 to 0.063, *p* = 0.004), alcohol drinking (*β* = 0.073, CI 0.048 to 0.099, *p* < 0.001), dietary (*β* = 0.023, CI 0.009 to 0.036, *p* = 0.001), hypertension (*β* = 0.024, CI 0.003 to 0.046, *p* = 0.028), diabetes (*β* = 0.052, CI 0.017 to 0.086, *p* = 0.004) and lipid disorder (*β* = 0.026, CI 0.009 to 0.042, *p* = 0.002, Figure 3). Moreover, higher HDI (*β* = 0.054, CI 0.006 to 0.102, *p* = 0.026), higher GDP per capita (*β* = 0.042, CI 0.005 to 0.079, *p* = 0.026), and higher prevalence of smoking (*β* = 0.021, CI 0.006 to 0.035, *p* = 0.005) were the only associations observed in the female population.

**FIGURE 4:**
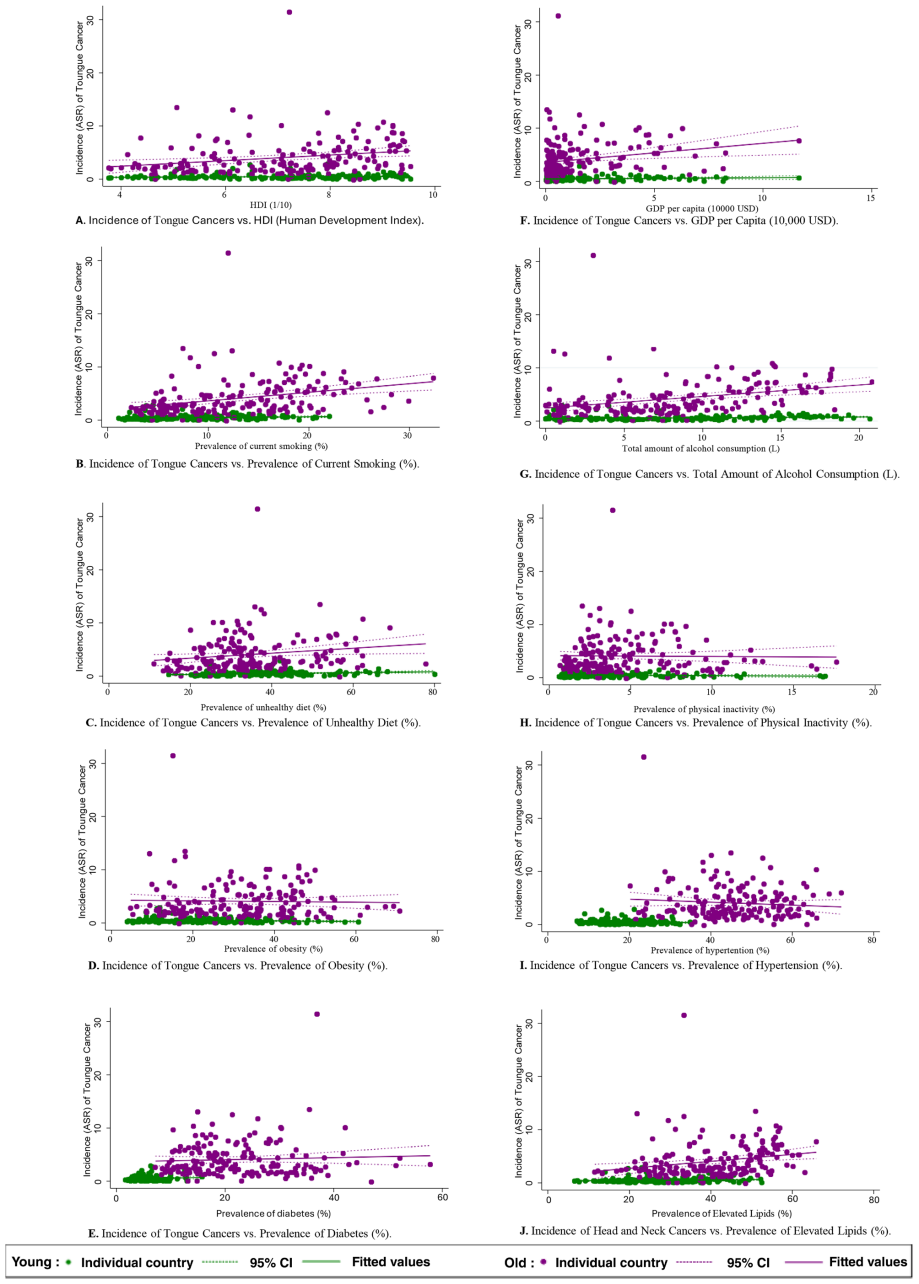
Associations between risk factors and tongue cancer by age.

Turning to the age groups, alcohol drinking (young: *β* = 0.016, CI 0.004 to 0.028, *p* = 0.007; old: *β* = 0.217, CI 0.120 to 0.314, *p* < 0.001) and dietary (young: *β* = 0.008, CI 0.002 to 0.014, *p* = 0.005; old: *β* = 0.047, CI 0.006 to 0.087. *p* = 0.024) were the common factors found in the young and old populations (Figure 4). Furthermore, in the old age group, tongue cancer was also positively associated with HDI (*β* = 0.522, CI 0.192 to 0.853, *p* = 0.002), GDP per capita (*β* = 0.359, CI 0.105 to 0.613, *p* = 0.006), higher prevalence of smoking (*β* = 0.161, CI 0.086 to 0.236, *p* < 0.001) and lipid disorder (*β* = 0.066, CI 0.023 to 0.1.9, *p* = 0.003). Moreover, obesity (*β* = −0.006, CI −0.012 to −0.001, *p* = 0.032) was the only negative association found in the young population.

### 3.5 Age‐ and Sex‐Specific Trend Analysis by Subtype

We found that an increasing trend was presented globally among 2003 and 2012, with 11 countries showing increases and only one country showing a drop. The largest increase could be found in Ireland (AAPC: 5.02, CI 2.47 to 4.63, *p* = 0.002), United Kingdom (AAPC: 4.97, CI 4.21 to 5.74, *p* < 0.001), Japan (AAPC: 3.78, CI 2.06 to 5.53, *p* = 0.001), Canada (AAPC: 3.74, CI 1.86 to 5.66, *p* < 0.001) and Denmark (AAPC: 3.62, CI 0.06 to 7.32, *p* = 0.047). Meanwhile, Philippines (AAPC: −6.37, CI −10.55 to −1.98, *p* = 0.011) showed the only significant drop (Figure S1, Figure S2, Table S2).

**FIGURE 5:**
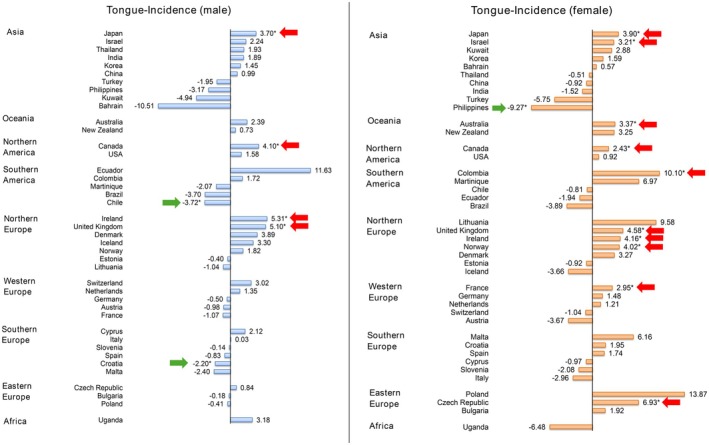
AAPC of tongue cancer incidence by sex, all ages.

**FIGURE 6:**
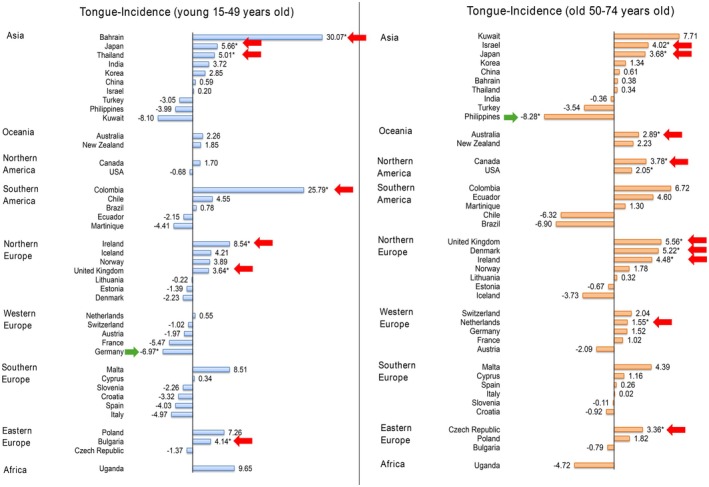
AAPC of tongue cancer incidence by ages, both sexes.

Also, a rising trend was reported in both male and female populations (Figure 5). For the male population, 4 countries and 2 countries showed growing trends and declining trends respectively, in which Ireland (AAPC: 5.31, CI 2.10 to 8.61, *p* = 0.005) and Chile (AAPC: −3.72, CI −6.00 to −1.38, *p* = 0.002) showed the greatest increase and decrease respectively. For the female population, 10 countries presented the increasing trends and only one country presented the decreases. The largest rises could be found in Colombia (AAPC: 10.10, CI 3.37 to 17.27, *p* = 0.008), Czech Republic (AAPC: 6.93, CI 4.11 to 9.82, *p* < 0.001), United Kingdom (AAPC: 4.58, CI 3.75 to 5.42, *p* < 0.001); Philippines (AAPC: −9.27, CI −14.31 to −3.94, *p* = 0.004) presented the only decline. Turning to the age groups, more countries reported increasing trends than decreasing trends in both groups (Figure 6). As for the young population, 7 countries found increasing trends and a country found a declining trend: Bahrain (AAPC: 30.07, CI 26.61 to 33.61, *p* < 0.001), Colombia (AAPC: 25.79, CI 2.39 to 54.53, *p* = 0.029), Ireland (AAPC: 8.54, CI 0.87 to 16.80, *p* = 0.033) showed the largest rises; Germany (AAPC: −6.97, CI −12.93 to −0.60, *p* = 0.036) was the only country reported a significant decline. As for the old population, 10 countries reported increasing trends and a country reported the only decrease. The greatest rises could be found in the United Kingdom (AAPC: 5.56, CI 4.46 to 6.66, *p* < 0.001), Denmark (AAPC: 5.22, CI 1.26 to 9.33, *p* = 0.016), Ireland (AAPC: 4.48, CI 1.77 to 7.27, *p* = 0.005). In the meantime, Philippines (AAPC: −8.28, CI −13.49 to −2.75, *p* = 0.009) presented the only drop.

## 4 Discussion

### 4.1 Summary of Major Findings

The present study examines the global incidence, associated risk factors, and temporal trends of tongue cancer across various subgroups. Significant geographic variation was observed among regions and countries, with the highest incidences reported in South‐Central Asia, Northern America, and Northern Europe. Additionally, a greater disease burden was identified in male and older populations. The study also found that tongue cancer was associated with higher HDI, GDP per capita, and a higher prevalence of smoking and alcohol consumption. Furthermore, an overall increasing trend was reported among 2003 and 2012 globally, especially in the female population, which reported the most evident increasing trend. Additionally, the Philippines presented the only drop in most of the subgroups.

### 4.2 Variation in the Disease Burden

For many decades, countries in the Indian subcontinent have reported a high incidence rate of intraoral cancers, including tongue cancer [42], which includes Bangladesh, India, Pakistan, and Sri Lanka, and these countries were found to have the highest ASRs of incidence in our study. An ethnic difference in oral cancer rate was also found in Central Asia and Southeast Asia [43]. The Tamils in Sri Lanka (Ratio of percentage: 1.88) and the Indians in Malaysia (Ratio of percentage: 5.69) were identified with the highest cancer risk as compared to other ethnic groups [44]. The possible reason could be due to cultural habits and differences in dietary habits, such as the chewing of betel‐quid [43, 45]. A greater disease burden was observed among the male population, which may be attributed to higher smoking rates and a greater prevalence of HPV infection [12]. In the US, the overall prevalence of oral HPV among men was 11.5% (95% CI: 9.8% to 13.1%), which was much higher than the incidence rate of 3.2% (95% CI: 2.7% to 3.8%) in women [46]. Globally, there is a huge difference in smoking rates between males and females, with 34.1% of males and 6.4% of females being current smokers [47]. Additionally, the observed burden difference among males and females may be partially attributed to the underdiagnosis and difficulty in accessing health services among the female population [48], especially in the low and middle‐income countries [49, 50].

The potential contribution of culture and social factors to the incidence of tongue cancer is multifaceted. Cultural practices such as tobacco or betel nut chewing, alcohol consumption, dietary habits, and exposure to environmental carcinogens can significantly impact the development of tongue cancer. Socioeconomic factors like access to healthcare, education, and awareness programs also play a crucial role. Cultural beliefs and attitudes towards seeking medical help, as well as social norms influencing lifestyle choices, can influence the prevalence of risk factors associated with tongue cancer. Additionally, disparities in healthcare infrastructure and resources based on cultural or social determinants may affect early detection, treatment outcomes, and overall incidence rates of tongue cancer within specific populations.

### 4.3 Risk Factors Associated With Tongue Cancer

The previous study found that current cigarette smokers had a 10.5 times higher risk (95% CI 3.2–34.1) of developing tongue cancer. Furthermore, an elevated risk was reported in those who consumed 56 or more glasses of wine per week (56–83 glasses of wine: OR 3.9, 95% CI 1.8 to 8.1; ≥ 84 glasses of wine: OR 8.8, 95% CI 3.4 to 22.6) [11]. However, the effect of beer and hard liquors on tongue cancer is not significant (consumed ≥ 14 glasses of beer per week: OR 1.1, 95% CI 0.6 to 2.0; consumed ≥ 7 glasses of hard liquor per week: OR 1.0, 95% CI 0.6 to 1.6) [11]. The carcinogenic compounds in tobacco smoke and the oxidative stress and mucosal damage caused by excessive alcohol consumption are well‐documented contributors to oral and tongue carcinogenesis [51, 52]. Furthermore, betel quid chewing was also found to be associated with oral cavity carcinoma and a group 1 human carcinogen as defined by WHO [53, 54]. In our study, unhealthy dietary habits were positively associated with the risk of having tongue cancer. In terms of dietary habits, butter intake was found to have a positive association with tongue cancer. Intermediate butter consumption was associated with a 2.5‐fold increased risk (95% CI 1.5 to 4.3), while high butter consumption was linked to a 2‐fold increased risk (95% CI 1.0 to 4.2) [11]. High intake of fresh fruits and vegetables seems to have a protective effect against tongue cancer (Green vegetables: OR 0.8, 95% CI 0.40 to 1.5; Carrots: OR 0.6, 95% CI 0.30 to 1.1; Fresh fruits: OR 0.8, 95% CI 0.50 to 1.3) [11]. The protective effects of fruits and vegetables are likely mediated through their antioxidant and anti‐inflammatory properties, which can counter the damaging effects of unhealthy dietary habits [55, 56]. Another possible risk factor for tongue cancer is HPV infection. HPV‐positive cases have been associated with better clinical outcomes compared to HPV‐negative cases, with a 5‐year survival rate of 80% versus 40%, even with less intensive treatment [57]. Furthermore, an increasing trend of HPV‐driven tongue cancers was also observed in multiple regions [58, 59], which aligned with our results and indicated a potential association of tongue cancer and HPV infection. The unexpected negative association between obesity and tongue cancer in the young population could likely be explained by distinct metabolic profiles, hormonal influences, and lifestyle behaviors prevalent in younger individuals. Metabolic factors linked to obesity in younger age groups might interact differently with cancer development pathways, potentially resulting in a protective effect against tongue cancer. Furthermore, hormonal fluctuations during early adulthood and unique genetic variations in this age group could modify the relationship between obesity and cancer risk. This study showed the association of diabetes and hyperlipidemia with tongue cancer. The association between diabetes and oral cancers, including tongue cancer, has shown conflicting results and is still debatable as a risk factor for oral premalignant lesions [60, 61, 62]. As for the association between hyperlipidemia and tongue cancer, a previous study found a difference in lipid profile between cancer patients and healthy individuals [63]. Since diabetes causes decreased cell adhesion and changes in the cell skeleton, diabetes could increase the risk of oral cancer [64]. However, the mechanisms of how diabetes and hyperlipidemia cause tongue cancer await further study to prove. Moreover, there was also a possibility of the presence of ecological fallacy in an ecological study, which led to generalization error [65].

This study also found an association between higher HDI and GDP per capita with the incidence of tongue cancer. The findings from this study may be the result of detection bias or registry density effects instead of etiological differences. Previous studies had indicated that poor‐quality health data and poor management of the health system were found among low and middle‐income countries [66]. The poor‐quality data could result in lower registry coverage, underreporting, and misclassification [67, 68], which may introduce underestimates of the disease burden, detection bias, or registry density effects in the findings observed. In addition, limited education and screening also contributed to the low incidence of cases due to a lack of preventive and disease control measures [67]. Further investigations focusing on these factors are warranted to validate and elucidate the underlying mechanisms driving this finding.

### 4.4 Incidence Trends of Tongue Cancer

A rising trend of tongue cancer was reported in Nordic countries among 2003 and 2012. The ASR incidence of tongue cancer for both sexes (age 20–79) increased persistently in 2000–2008 [69]. Our study reported a significant rising incidence in Denmark, and other nearby countries in Northern Europe such as Ireland and the United Kingdom reported the largest rises. The scholars suggested that the possible causes of the rise are still unclear, as the prevalence of smoking and alcohol is decreasing and oral hygiene has improved in the past few decades, in those countries [69]. Our study revealed a more evident increasing trend in the female population. However, a previous study reported a greater increase for women in some countries only. The annual rate of incidence increase was higher in women compared to men in Austria (2.8%), Bulgaria (7.5%), and Ireland (9.3%). Conversely, in England, Denmark, Sweden, and parts of the United States, a higher incidence increase was observed in men compared to women, with percentages ranging from 0.4% to 2.0%. Furthermore, a decreasing trend was observed in the Philippines for several subgroups; this finding was also confirmed by the previous literature [70, 71]. The declining use of betel quid and the increasing health consciousness among individuals may explain the trend [70, 71]. The Philippine Cancer Control Program begun in 1988, discussed and provided recommendations for the causation and prevention of oral cavity cancer [72]. As the program offered examination for high‐risk patients, regular dental examinations and counseling for tobacco and alcohol abuse adolescents [72]. The trends could also be the result of the successful public health policy implementation which could be followed by other governments to reduce the incidence of tongue cancer. The current study merged the tongue cancer of the base of tongue (C01), other and unspecified parts of the tongue (C02) and investigated the incidence, risk factors and trends of tongue cancer. However, part of the previous studies separated the subsites and investigated incidence and trends. A Brazilian study revealed the cancer incidence rates had increased for base of tongue cancer in both sexes and in the unspecified parts of the tongue in women from 2000 to 2014 [73]. For the unspecified parts of the tongue, a global study that included 22 international registries and revealed a general increasing incidence trend worldwide with mixed trends in the differences in incidence increase among the two sexes for different registry data [74]. The study also indicated the increasing trends among subjects younger than 45 years old [74]. Furthermore, a Korean study also indicated an increasing trend in both base and unspecified sites of the tongue, with significant increasing trends observed among females for C02 tongue cancer [75]. Despite the etiologic differences between the base and the nonspecific parts of tongue cancer and the differences between countries, general rising trends were observed among both sites of the tongue and the female and younger populations, showing significant increasing trends, which aligned with the findings of this study.

Moreover, the HPV prevalence had been found to be highest among women less than 25 years of age and declined in older age [76]. As HPV was the risk factor for cancer in the base of the tongue, the current incidence of HPV may be explained by the rise in female incidence worldwide.

### 4.5 Implications

The findings carry significant implications for public health policies aimed at reducing the incidence of tongue cancer. Policymakers should prioritize the implementation of comprehensive prevention programs targeting key risk factors, including smoking, alcohol consumption, and HPV infection. These programs should include robust tobacco control measures, targeted HPV vaccination campaigns, and health education initiatives to raise awareness about tongue cancer and promote healthy lifestyle behaviors. Public health policies should also emphasize the importance of high‐risk population screenings and early detection, along with research and surveillance efforts to monitor trends and guide evidence‐based interventions. Additionally, policies should aim to reduce health disparities by ensuring equitable access to prevention, screening, and treatment services across diverse populations.

Furthermore, our study revealed global trends and risk factors associated with tongue cancer, which may contribute to the surgical management of this condition in several important ways. First, identifying specific risk factors could enhance preoperative assessments, allowing for more tailored surgical approaches that may improve patient outcomes. Second, understanding global trends may assist in guiding resource allocation and the development of targeted surgical protocols that address varying disease burdens across different regions. Additionally, insights into factors influencing disease progression could support surgical decision‐making, potentially enabling the adoption of more effective techniques.

This study provides valuable insights and connections with other cancers and diseases by highlighting shared risk factors such as tobacco use, alcohol consumption, and HPV infection, which are also linked to malignancies like lip, mouth, throat and cervical cancers. Additionally, findings on geographic and socioeconomic variations in tongue cancer incidence can illuminate health disparities relevant to multiple cancers, informing targeted public health strategies. Effective prevention programs and early detection strategies developed for tongue cancer can be adapted for other cancers, enhancing overall cancer control efforts. This research may also foster interdisciplinary connections between oral health and systemic diseases, such as cardiovascular disease and diabetes, enhancing our understanding of the interplay between various health conditions.

### 4.6 Strengths and Limitations

Previous studies have often grouped tongue cancer with other types of oral cancer, which can obscure its unique characteristics. Tongue cancer exhibits distinct disease burdens, risk factors, and trends that differ from other oral cancers. This study addresses these differences by utilizing high‐quality data from cancer registries spanning 186 countries, enabling a comprehensive analysis of the global incidence, risk factors, and temporal trends specifically related to tongue cancer. By providing up‐to‐date, large‐scale insights and detailed estimates, the study enhances our understanding of tongue cancer's unique epidemiology. This focused approach facilitates the identification of specific risk factors and trends, informing more effective prevention and treatment strategies tailored to tongue cancer, ultimately contributing to improved public health outcomes.

However, several limitations must be acknowledged. These include issues related to the data source, study design, statistical analysis, and external factors. Firstly, the analysis of risk factors was conducted at the country level rather than the individual level; the inference of individual‐level risk relationships or establishing causality inference is not available due to the data source constraints. Secondly, certain confounding variables that may exist at the individual level could not be controlled for in the current study, potentially influencing the observed associations. Risk factors, such as Betel quid chewing, chewing tobacco, and HPV were important risk factors for tongue cancer. However, these unmeasured factors may also affect the incidence and are not included in this study due to the availability of data sources. Thirdly, comparing cancer data across countries may not always be appropriate due to the frequent changes that cancer registries undergo. These changes can impact the consistency and comparability of the data. Nevertheless, comparing data for countries, regions, and sexes during the same period should provide relatively reliable insights. Furthermore, the estimates provided in this study do not reflect the full effect of the COVID‐19 pandemic. The lockdown measures implemented during the pandemic may have led to delays in cancer diagnoses, potentially affecting the reported incidence rates. The COVID‐19 pandemic may also lead to changes in trends of tongue cancer, given a dramatic change in lifestyle during this period. Fifthly, since our data source does not include the ethnicity of each country in the incidence of tongue cancer and in the data of AAPC, the effect of ethnicity on the incidence of tongue cancer remains unknown. Sixthly, CI5 Plus data only covered pre‐2012 data; the findings of this study may not reflect the pattern of post‐2012 periods. A further updated database or study should be conducted to update the trends of tongue cancer worldwide. Lastly, the study did not separate the analysis based on distinct clinical characteristics between the base of the tongue and the anterior tongue. Considering the differences between these regions could provide further insights into tongue cancer patterns and risk factors.

## 5 Conclusions

A notable variation was observed in the geographic distribution of tongue cancer, with South‐Central Asia having the highest disease burden. Also, the higher incidence of tongue cancer in males is likely explained by the higher prevalence of cigarette smoking and alcohol drinking among males; this highlights the need for intensive lifestyle modifications. Additionally, HPV infection and cultural habits, such as betel‐quid chewing, were identified as significant risk factors for tongue cancer. This highlights the importance of early prevention efforts and the promotion of health awareness to address these specific risks. Furthermore, the mechanism of the current increasing global trend of tongue cancer, especially in Northern Europe and the female population, remains unclear with the decreasing prevalence of smoking and alcohol drinking, as well as the improved oral hygiene of the region. Further study could be conducted to explore the factors contributing to the rising trend of tongue cancer at a global level and in certain groups.

## Author Contributions

**Junjie Huang:** conceptualization (equal), writing – original draft (equal). **Wing Sze Pang:** writing – original draft (equal). **Claire Chenwen Zhong:** writing – original draft (equal). **Fung Yu Mak:** writing – original draft (equal). **Sze Chai Chan:** data curation (lead), formal analysis (lead). **Jinqiu Yuan:** writing – review and editing (equal). **Lin Zhang:** writing – review and editing (equal). **Wanghong Xu:** writing – review and editing (equal). **Zhi‐Jie Zheng:** writing – review and editing (equal). **Zigui Chen:** writing – review and editing (equal). **Jason Y. K. Chan:** writing – review and editing (equal). **Martin C. S. Wong:** conceptualization (equal), writing – review and editing (equal).

## Funding

The authors have nothing to report.

## Ethics Statement

This study was approved by the Survey and Behavioral Research Ethics Committee, The Chinese University of Hong Kong (No. SBRE‐20‐332).

## Consent

As the data utilized in this retrospective analysis were de‐identified and contained no personal details, the committee waived the requirement for informed consent.

## Conflicts of Interest

The authors declare no conflicts of interest.

## Supporting information


**Data S1:** Flow Chart. Steps for trend analysis using Joinpoint regression.
**Table S1:** Global incidence of tongue cancer by HDI in 2020.
**Table S2:** Results of Joinpoint regression for trend analysis.
**Figure S1:** Tongue cancer incidence trends for individual countries.
**Figure S2:** Plots of the Joinpoint regression for trend analysis.

## Data Availability

The datasets used and/or analyzed during the current study are available from the corresponding author on reasonable request.
