# Supplementary material for: Incidence, Risk Factors, and Temporal Trends of Tongue Cancer: A Population‐Based Study
Source: Cancer Med. 2026 Feb 3;15(2):e71435. doi: 10.1002/cam4.71435 (PMC12868936; doi:10.1002/cam4.71435)
Supplement: Supplementary file 1 — Data S1: Flow Chart. Steps for trend analysis using Joinpoint regression. Table S1: Global incidence of tongue cancer by HDI in 2020. Table S2: Results of Joinpoint regression for trend analysis. Figure S1: Tongue cancer incidence trends for individual countries. Figure S2: Plots of the Joinpoint regression for trend analysis. [file CAM4-15-e71435-s001.docx]

**Supplementary Legends**

**Supplementary Flow Chart.** Steps for trend analysis using Joinpoint regression

**Supplementary Table 1.** Global incidence of tongue cancer by HDI in 2020

**Supplementary Table 2.** Results of Joinpoint regression for trend analysis

**Supplementary Figure 1.** Tongue cancer incidence trends for individual countries

**Supplementary Figure 2.** Plots of the Joinpoint regression for trend analysis

**
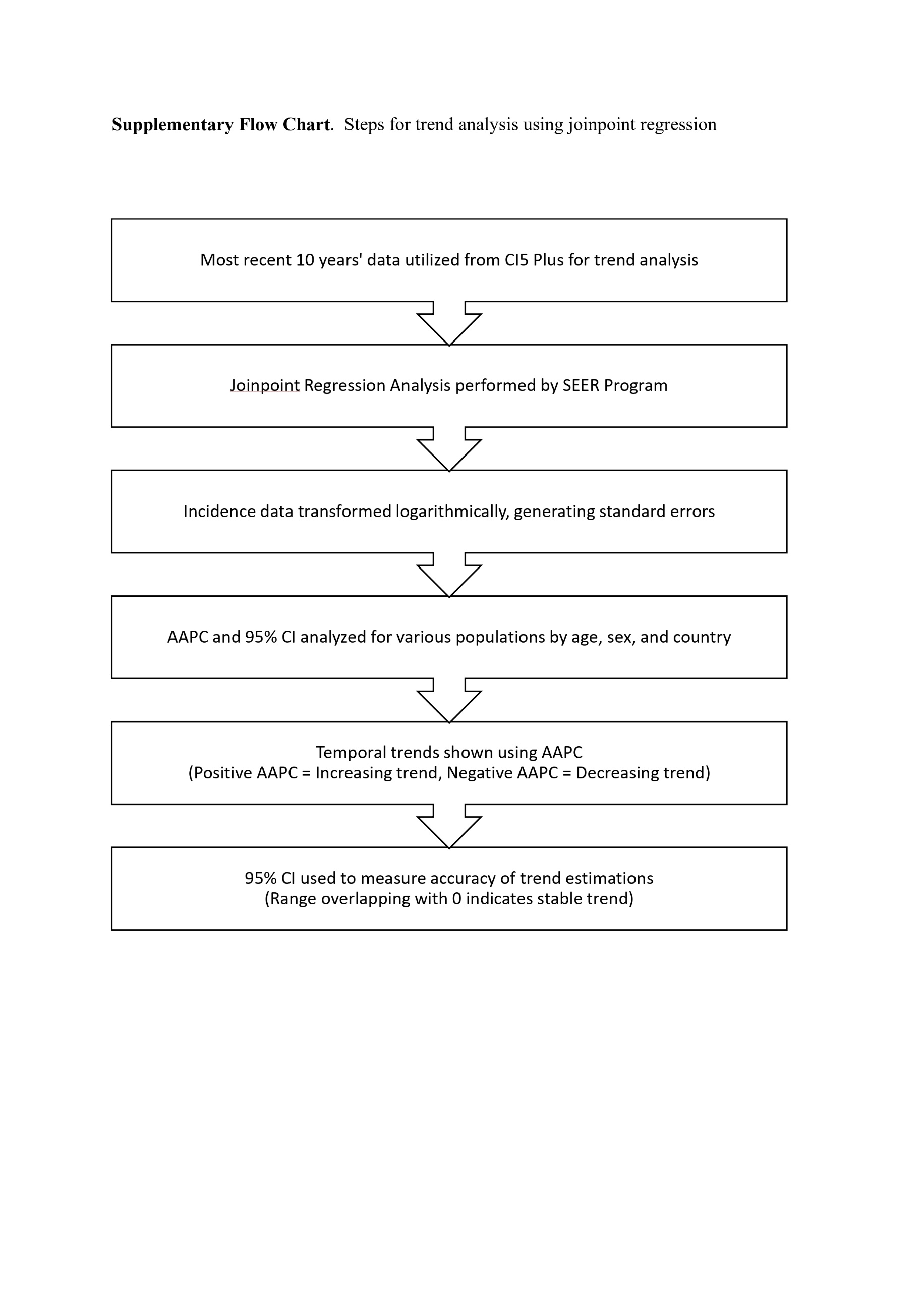
**

**Supplementary Table 1.** Global incidence of tongue cancer by HDI in 2020

|  |  | Median | Q1 | Q3 |
| --- | --- | --- | --- | --- |
| Both | Very high | 1.4 | 0.96 | 1.9 |
|  | High | 0.73 | 0.505 | 1 |
|  | Medium | 0.84 | 0.53 | 1.3 |
|  | Low | 0.56 | 0.46 | 0.67 |
|  |  |  |  |  |
| Male | Very high | 2.05 | 1.35 | 2.825 |
|  | High | 0.92 | 0.66 | 1.75 |
|  | Medium | 1.2 | 0.525 | 2.3 |
|  | Low | 0.72 | 0.55 | 0.88 |
|  |  |  |  |  |
| Female | Very high | 0.8 | 0.6175 | 1 |
|  | High | 0.47 | 0.35 | 0.61 |
|  | Medium | 0.53 | 0.285 | 0.795 |
|  | Low | 0.38 | 0.31 | 0.57 |
|  |  |  |  |  |
| Young | Very high | 0.61 | 0.33 | 0.845 |
|  | High | 0.31 | 0.16 | 0.49 |
|  | Medium | 0.39 | 0.185 | 0.585 |
|  | Low | 0.26 | 0.22 | 0.34 |
|  |  |  |  |  |
| Old | Very high | 5.95 | 3.525 | 7.75 |
|  | High | 2.7 | 1.7 | 4.35 |
|  | Medium | 3.3 | 1.6 | 4.9 |
|  | Low | 2.1 | 1.6 | 2.5 |

**Supplementary Table 2.** Results of Joinpoint regression for trend analysis

a) Male

| **Region** | **AAPC** | **Lower CI** | **Upper CI** | **p-value** | **Significant** |
| --- | --- | --- | --- | --- | --- |
| ***Asia*** |  |  |  |  |  |
| Bahrain | -10.51 | -24.17 | 5.63 | 0.161 |  |
| China | 0.99 | -3.23 | 5.39 | 0.610 |  |
| India | 1.89 | -0.28 | 4.11 | 0.080 |  |
| Israel | 2.24 | -1.70 | 6.34 | 0.230 |  |
| Japan | 3.70 | 1.96 | 5.46 | 0.001 | * |
| Korea | 1.45 | -0.29 | 3.21 | 0.091 |  |
| Kuwait | -4.94 | -17.03 | 8.92 | 0.416 |  |
| Philippines | -3.17 | -8.27 | 2.22 | 0.208 |  |
| Thailand | 1.93 | -2.54 | 6.60 | 0.354 |  |
| Turkey | -1.95 | -7.34 | 3.76 | 0.446 |  |
| ***Oceania*** |  |  |  |  |  |
| Australia | 2.39 | -0.30 | 5.15 | 0.082 |  |
| New Zealand | 0.73 | -3.07 | 4.68 | 0.711 |  |
| ***Northern America*** |  |  |  |  |  |
| Canada | 4.10 | 1.96 | 6.28 | <0.001 | * |
| USA | 1.58 | -0.05 | 3.23 | 0.057 |  |
| ***Southern America*** |  |  |  |  |  |
| Brazil | -3.70 | -10.57 | 3.71 | 0.275 |  |
| Chile | -3.72 | -6.00 | -1.38 | 0.002 | * |
| Colombia | 1.72 | -8.39 | 12.93 | 0.717 |  |
| Ecuador | 11.63 | -6.07 | 32.65 | 0.180 |  |
| Martinique | -2.07 | -13.10 | 10.36 | 0.697 |  |
| ***Northern Europe*** |  |  |  |  |  |
| Denmark | 3.89 | -0.15 | 8.09 | 0.058 |  |
| Estonia | -0.40 | -5.03 | 4.45 | 0.851 |  |
| Iceland | 3.30 | -6.99 | 14.74 | 0.496 |  |
| Ireland | 5.31 | 2.10 | 8.61 | 0.005 | * |
| Lithuania | -1.04 | -3.51 | 1.50 | 0.370 |  |
| Norway | 1.82 | -2.02 | 5.81 | 0.311 |  |
| United Kingdom | 5.10 | 4.18 | 6.03 | <0.001 | * |
| ***Western Europe*** |  |  |  |  |  |
| Austria | -0.98 | -3.65 | 1.75 | 0.428 |  |
| France | -1.07 | -2.87 | 0.77 | 0.215 |  |
| Germany | -0.50 | -4.18 | 3.33 | 0.769 |  |
| Netherlands | 1.35 | -0.94 | 3.69 | 0.214 |  |
| Switzerland | 3.02 | -3.06 | 9.48 | 0.337 |  |
| ***Southern Europe*** |  |  |  |  |  |
| Croatia | -2.20 | -3.83 | -0.53 | 0.016 | * |
| Cyprus | 2.12 | -14.43 | 21.86 | 0.792 |  |
| Italy | 0.03 | -4.01 | 4.25 | 0.985 |  |
| Malta | -2.40 | -14.95 | 12.01 | 0.695 |  |
| Slovenia | -0.14 | -5.60 | 5.63 | 0.954 |  |
| Spain | -0.83 | -4.15 | 2.62 | 0.591 |  |
| ***Eastern Europe*** |  |  |  |  |  |
| Bulgaria | -0.18 | -2.49 | 2.17 | 0.860 |  |
| Czech Republic | 0.84 | -0.15 | 1.84 | 0.087 |  |
| Poland | -0.41 | -8.39 | 8.27 | 0.912 |  |
| ***Africa*** |  |  |  |  |  |
| Uganda | 3.18 | -8.17 | 15.93 | 0.553 |  |

AAPC, annual percentage change; CI, confidence interval; * p values less than 0·05.

b) Female

| **Region** | **AAPC** | **Lower CI** | **Upper CI** | **p-value** | **Significant** |
| --- | --- | --- | --- | --- | --- |
| ***Asia*** |  |  |  |  |  |
| Bahrain | 0.57 | -8.61 | 10.66 | 0.908 |  |
| China | -0.92 | -4.09 | 2.36 | 0.531 |  |
| India | -1.52 | -5.02 | 2.12 | 0.359 |  |
| Israel | 3.21 | 0.03 | 6.48 | 0.048 | * |
| Japan | 3.90 | 0.64 | 7.26 | 0.024 | * |
| Korea | 1.59 | -2.83 | 6.21 | 0.437 |  |
| Kuwait | 2.88 | -12.70 | 21.23 | 0.701 |  |
| Philippines | -9.27 | -14.31 | -3.94 | 0.004 | * |
| Thailand | -0.51 | -6.80 | 6.21 | 0.862 |  |
| Turkey | -5.75 | -14.46 | 3.85 | 0.197 |  |
| ***Oceania*** |  |  |  |  |  |
| Australia | 3.37 | 0.83 | 5.96 | 0.015 | * |
| New Zealand | 3.25 | -1.36 | 8.08 | 0.145 |  |
| ***Northern America*** |  |  |  |  |  |
| Canada | 2.43 | 0.10 | 4.81 | 0.043 | * |
| USA | 0.92 | -0.58 | 2.45 | 0.196 |  |
| ***Southern America*** |  |  |  |  |  |
| Brazil | -3.89 | -16.69 | 10.88 | 0.586 |  |
| Chile | -0.81 | -24.10 | 29.63 | 0.953 |  |
| Colombia | 10.10 | 3.37 | 17.27 | 0.008 | * |
| Ecuador | -1.94 | -9.61 | 6.37 | 0.594 |  |
| Martinique | 6.97 | -7.87 | 24.20 | 0.329 |  |
| ***Northern Europe*** |  |  |  |  |  |
| Denmark | 3.27 | -0.92 | 7.64 | 0.111 |  |
| Estonia | -0.92 | -10.50 | 9.68 | 0.839 |  |
| Iceland | -3.66 | -16.13 | 10.66 | 0.552 |  |
| Ireland | 4.16 | 0.55 | 7.90 | 0.029 | * |
| Lithuania | 9.58 | -0.51 | 20.69 | 0.061 |  |
| Norway | 4.02 | 0.15 | 8.03 | 0.043 | * |
| United Kingdom | 4.58 | 3.75 | 5.42 | <0.001 | * |
| ***Western Europe*** |  |  |  |  |  |
| Austria | -3.67 | -7.50 | 0.31 | 0.066 |  |
| France | 2.95 | 0.71 | 5.25 | 0.016 | * |
| Germany | 1.48 | -5.70 | 9.20 | 0.657 |  |
| Netherlands | 1.21 | -1.09 | 3.57 | 0.262 |  |
| Switzerland | -1.04 | -6.18 | 4.40 | 0.665 |  |
| ***Southern Europe*** |  |  |  |  |  |
| Croatia | 1.95 | -3.73 | 7.96 | 0.459 |  |
| Cyprus | -0.97 | -13.51 | 13.39 | 0.872 |  |
| Italy | -2.96 | -6.88 | 1.12 | 0.152 |  |
| Malta | 6.16 | -10.87 | 26.43 | 0.453 |  |
| Slovenia | -2.08 | -11.42 | 8.24 | 0.641 |  |
| Spain | 1.74 | -1.51 | 5.10 | 0.255 |  |
| ***Eastern Europe*** |  |  |  |  |  |
| Bulgaria | 1.92 | -5.60 | 10.05 | 0.583 |  |
| Czech Republic | 6.93 | 4.11 | 9.82 | <0.001 | * |
| Poland | 13.87 | -4.52 | 35.81 | 0.128 |  |
| ***Africa*** |  |  |  |  |  |
| Uganda | -6.48 | -20.46 | 9.95 | 0.368 |  |

AAPC, annual percentage change; CI, confidence interval; * p values less than 0·05.

c) Young

| **Region** | **AAPC** | **Lower CI** | **Upper CI** | **p-value** | **Significant** |
| --- | --- | --- | --- | --- | --- |
| ***Asia*** |  |  |  |  |  |
| Bahrain | 30.07 | 26.61 | 33.61 | <0.001 | * |
| China | 0.59 | -5.06 | 6.57 | 0.822 |  |
| India | 3.72 | -0.73 | 8.36 | 0.091 |  |
| Israel | 0.20 | -4.34 | 4.95 | 0.925 |  |
| Japan | 5.66 | 0.88 | 10.66 | 0.025 | * |
| Korea | 2.85 | -1.39 | 7.27 | 0.162 |  |
| Kuwait | -8.10 | -23.44 | 10.32 | 0.317 |  |
| Philippines | -3.99 | -13.53 | 6.60 | 0.395 |  |
| Thailand | 5.01 | 1.52 | 8.63 | 0.010 | * |
| Turkey | -3.05 | -8.09 | 2.27 | 0.218 |  |
| ***Oceania*** |  |  |  |  |  |
| Australia | 2.26 | -0.74 | 5.36 | 0.122 |  |
| New Zealand | 1.85 | -2.49 | 6.39 | 0.360 |  |
| ***Northern America*** |  |  |  |  |  |
| Canada | 1.70 | -1.73 | 5.25 | 0.290 |  |
| USA | -0.68 | -2.31 | 0.98 | 0.371 |  |
| ***Southern America*** |  |  |  |  |  |
| Brazil | 0.78 | -5.40 | 7.37 | 0.785 |  |
| Chile | 4.55 | -2.82 | 12.48 | 0.233 |  |
| Colombia | 25.79 | 2.39 | 54.53 | 0.029 | * |
| Ecuador | -2.15 | -17.29 | 15.75 | 0.773 |  |
| Martinique | -4.41 | -15.82 | 8.55 | 0.437 |  |
| ***Northern Europe*** |  |  |  |  |  |
| Denmark | -2.23 | -8.49 | 4.47 | 0.456 |  |
| Estonia | -1.39 | -10.19 | 8.27 | 0.739 |  |
| Iceland | 4.21 | -7.01 | 16.77 | 0.428 |  |
| Ireland | 8.54 | 0.87 | 16.80 | 0.033 | * |
| Lithuania | -0.22 | -8.32 | 8.59 | 0.959 |  |
| Norway | 3.89 | -3.78 | 12.17 | 0.285 |  |
| United Kingdom | 3.64 | 2.08 | 5.22 | 0.001 | * |
| ***Western Europe*** |  |  |  |  |  |
| Austria | -1.97 | -6.59 | 2.88 | 0.370 |  |
| France | -5.47 | -10.74 | 0.11 | 0.054 |  |
| Germany | -6.97 | -12.93 | -0.60 | 0.036 | * |
| Netherlands | 0.55 | -2.37 | 3.57 | 0.678 |  |
| Switzerland | -1.02 | -10.79 | 9.82 | 0.826 |  |
| ***Southern Europe*** |  |  |  |  |  |
| Croatia | -3.32 | -6.87 | 0.36 | 0.071 |  |
| Cyprus | 0.34 | -11.31 | 13.53 | 0.950 |  |
| Italy | -4.97 | -10.97 | 1.43 | 0.109 |  |
| Malta | 8.51 | -5.29 | 24.33 | 0.239 |  |
| Slovenia | -2.26 | -15.87 | 13.56 | 0.735 |  |
| Spain | -4.03 | -8.65 | 0.83 | 0.091 |  |
| ***Eastern Europe*** |  |  |  |  |  |
| Bulgaria | 4.14 | 0.29 | 8.14 | 0.038 | * |
| Czech Republic | -1.37 | -5.06 | 2.48 | 0.431 |  |
| Poland | 7.26 | -2.25 | 17.70 | 0.120 |  |
| ***Africa*** |  |  |  |  |  |
| Uganda | 9.65 | -8.47 | 31.36 | 0.318 |  |

AAPC, annual percentage change; CI, confidence interval; * p values less than 0·05.

d) Old

| **Region** | **AAPC** | **Lower CI** | **Upper CI** | **p-value** | **Significant** |
| --- | --- | --- | --- | --- | --- |
| ***Asia*** |  |  |  |  |  |
| Bahrain | 0.38 | -14.19 | 17.42 | 0.957 |  |
| China | 0.61 | -2.41 | 3.73 | 0.656 |  |
| India | -0.36 | -3.12 | 2.48 | 0.776 |  |
| Israel | 4.02 | 0.65 | 7.50 | 0.025 | * |
| Japan | 3.68 | 1.61 | 5.80 | 0.003 | * |
| Korea | 1.34 | -0.25 | 2.95 | 0.088 |  |
| Kuwait | 7.71 | -13.34 | 33.88 | 0.503 |  |
| Philippines | -8.28 | -13.49 | -2.75 | 0.009 | * |
| Thailand | 0.34 | -3.98 | 4.86 | 0.864 |  |
| Turkey | -3.54 | -10.19 | 3.59 | 0.277 |  |
| ***Oceania*** |  |  |  |  |  |
| Australia | 2.89 | 0.53 | 5.31 | 0.016 | * |
| New Zealand | 2.23 | -1.77 | 6.39 | 0.239 |  |
| ***Northern America*** |  |  |  |  |  |
| Canada | 3.78 | 2.35 | 5.22 | <0.001 | * |
| USA | 2.05 | 1.13 | 2.99 | 0.001 | * |
| ***Southern America*** |  |  |  |  |  |
| Brazil | -6.90 | -15.27 | 2.30 | 0.118 |  |
| Chile | -6.32 | -20.07 | 9.81 | 0.421 |  |
| Colombia | 6.72 | -3.54 | 18.07 | 0.176 |  |
| Ecuador | 4.60 | -11.26 | 23.29 | 0.546 |  |
| Martinique | 1.30 | -12.58 | 17.38 | 0.845 |  |
| ***Northern Europe*** |  |  |  |  |  |
| Denmark | 5.22 | 1.26 | 9.33 | 0.016 | * |
| Estonia | -0.67 | -5.27 | 4.16 | 0.753 |  |
| Iceland | -3.73 | -15.48 | 9.65 | 0.519 |  |
| Ireland | 4.48 | 1.77 | 7.27 | 0.005 | * |
| Lithuania | 0.32 | -2.45 | 3.17 | 0.797 |  |
| Norway | 1.78 | -1.60 | 5.29 | 0.263 |  |
| United Kingdom | 5.56 | 4.46 | 6.66 | <0.001 | * |
| ***Western Europe*** |  |  |  |  |  |
| Austria | -2.09 | -4.85 | 0.76 | 0.128 |  |
| France | 1.02 | -0.11 | 2.17 | 0.072 |  |
| Germany | 1.52 | -1.19 | 4.31 | 0.233 |  |
| Netherlands | 1.55 | 0.38 | 2.73 | 0.015 | * |
| Switzerland | 2.04 | -2.24 | 6.51 | 0.309 |  |
| ***Southern Europe*** |  |  |  |  |  |
| Croatia | -0.92 | -3.32 | 1.54 | 0.410 |  |
| Cyprus | 1.16 | -12.06 | 16.36 | 0.854 |  |
| Italy | 0.02 | -3.77 | 3.96 | 0.990 |  |
| Malta | 4.39 | -9.78 | 20.79 | 0.516 |  |
| Slovenia | -0.11 | -3.74 | 3.66 | 0.949 |  |
| Spain | 0.26 | -2.95 | 3.59 | 0.857 |  |
| ***Eastern Europe*** |  |  |  |  |  |
| Bulgaria | -0.79 | -3.02 | 1.48 | 0.441 |  |
| Czech Republic | 3.36 | 2.50 | 4.23 | <0.001 | * |
| Poland | 1.82 | -5.37 | 9.55 | 0.586 |  |
| ***Africa*** |  |  |  |  |  |
| Uganda | -4.72 | -15.78 | 7.78 | 0.392 |  |

AAPC, annual percentage change; CI, confidence interval; * p values less than 0·05.

**Supplementary Figure 1.** Tongue cancer incidence trends for individual countries

| **Asia** | | |
| --- | --- | --- |
|  |  |  |
|  |  |  |
|  |  |  |
|  |  |  |

| **Oceania** | | |
| --- | --- | --- |
|  |  |  |
| **Northern America** | | |
|  |  |  |

| **Southern America** | | |
| --- | --- | --- |
|  |  |  |
|  |  |  |

| **Northern Europe** | | |
| --- | --- | --- |
|  |  |  |
|  |  |  |
|  |  |  |
| **Western Europe** | | |
|  |  |  |
|  |  |  |

| **Southern Europe** | | |
| --- | --- | --- |
|  |  |  |
|  |  |  |

| **Eastern Europe** | | |
| --- | --- | --- |
|  |  |  |
| **Africa** | | |
|  |  |  |

**Supplementary Figure 2.** Plots of the joinpoint regression for trend analysis

a) Male

| **Asia** | |
| --- | --- |
| 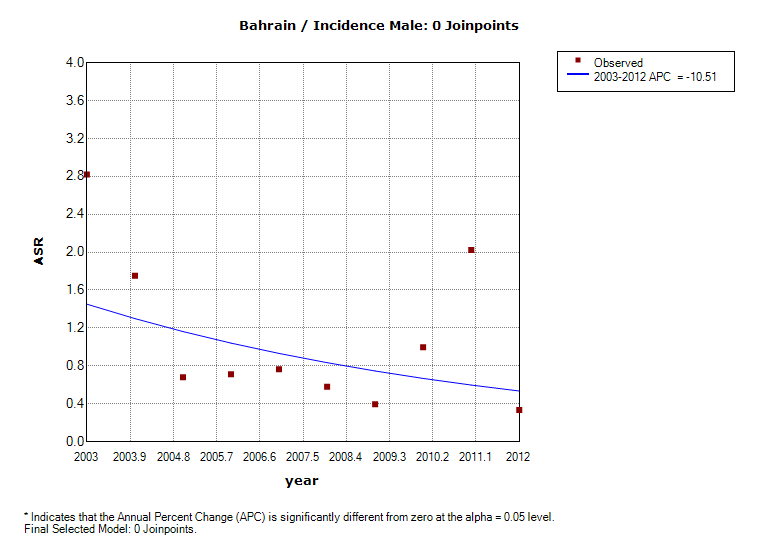 | 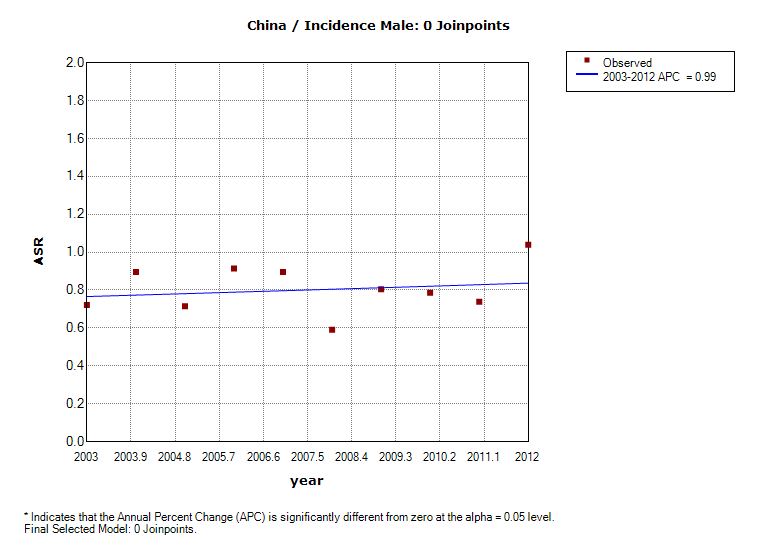 |
| 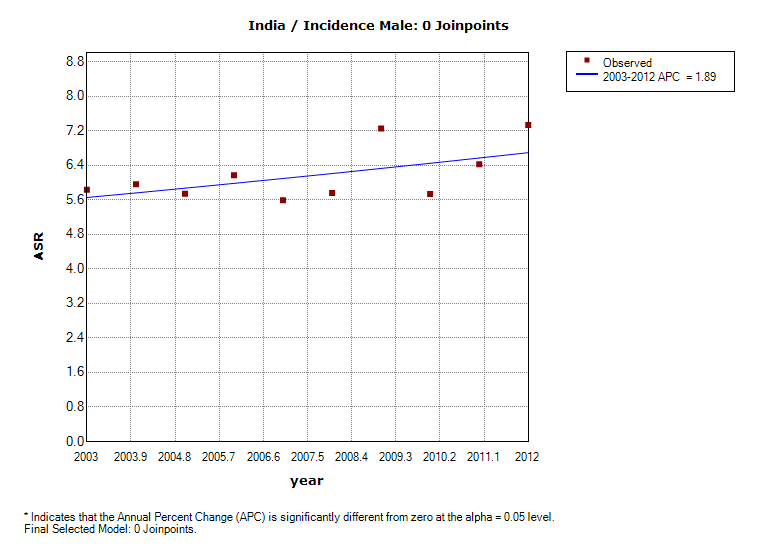 | 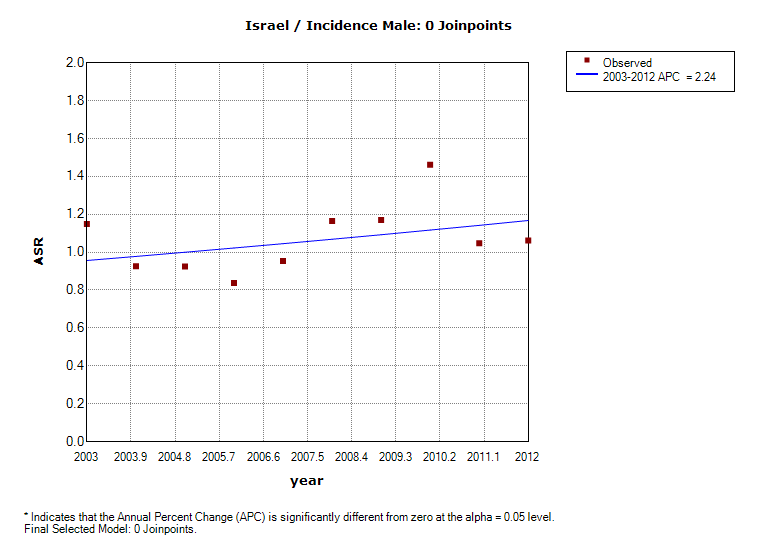 |
| 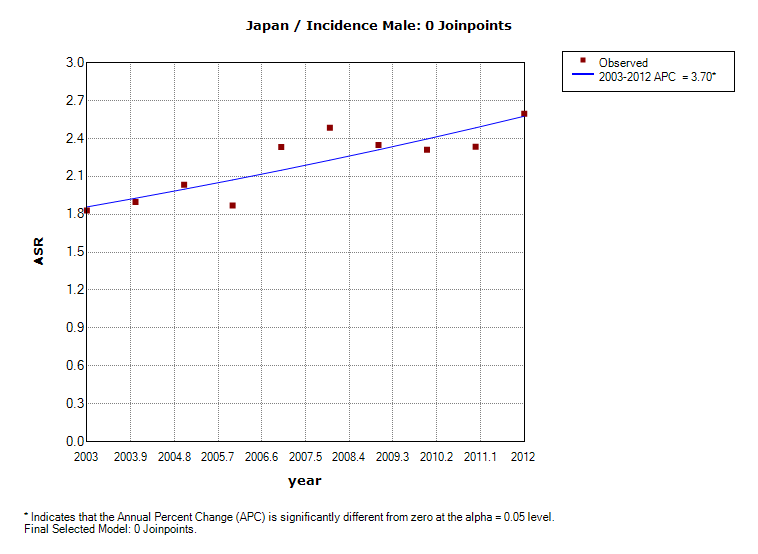 | 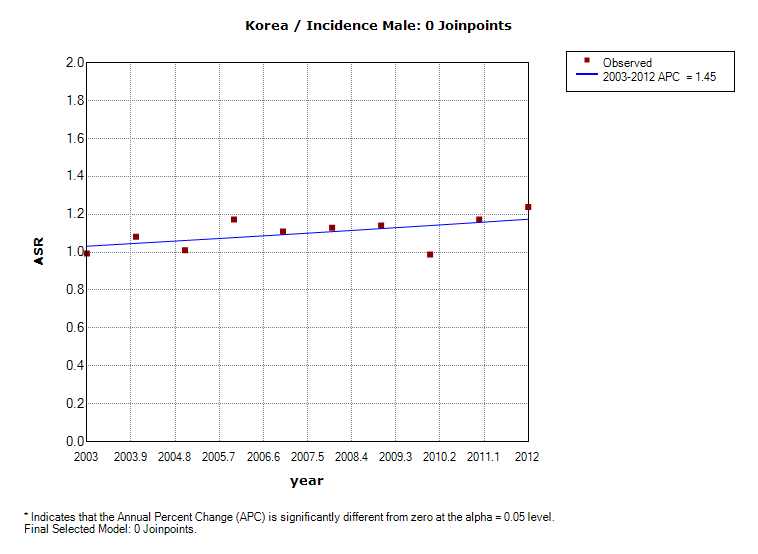 |
| 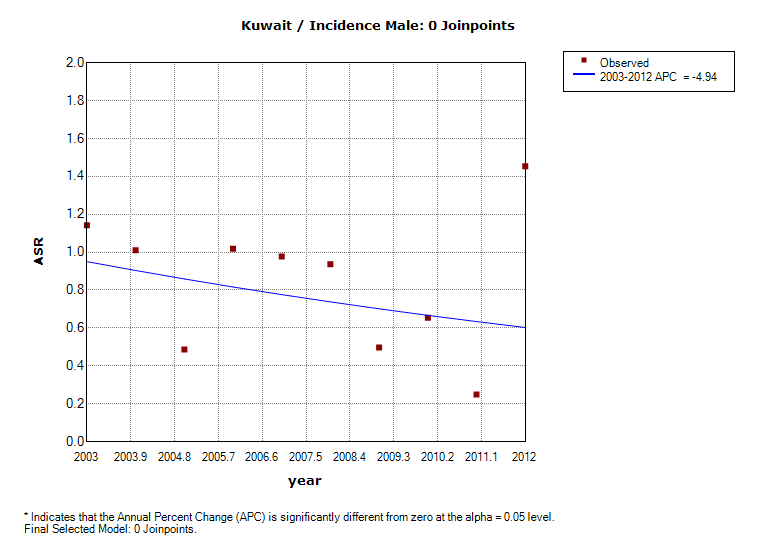 | 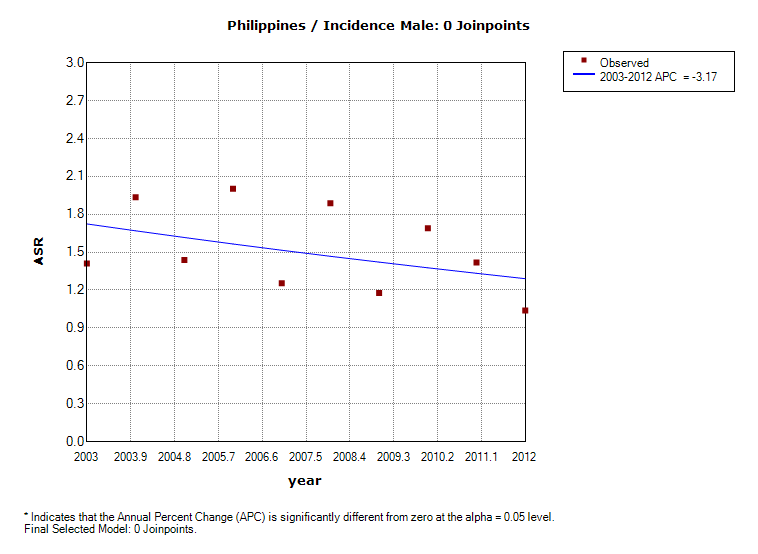 |
| 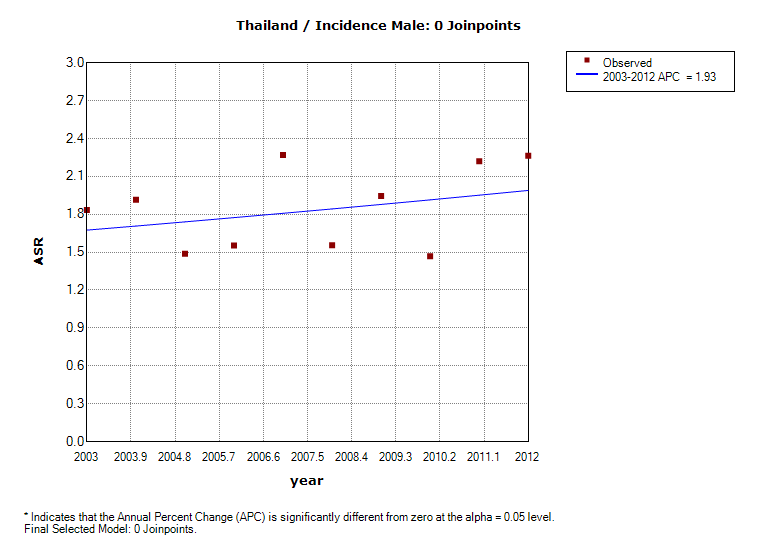 | 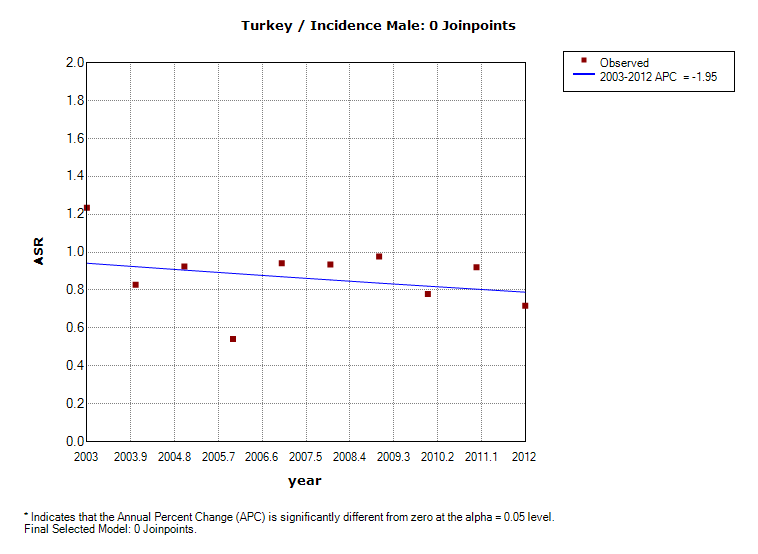 |
| **Oceania** | |
| 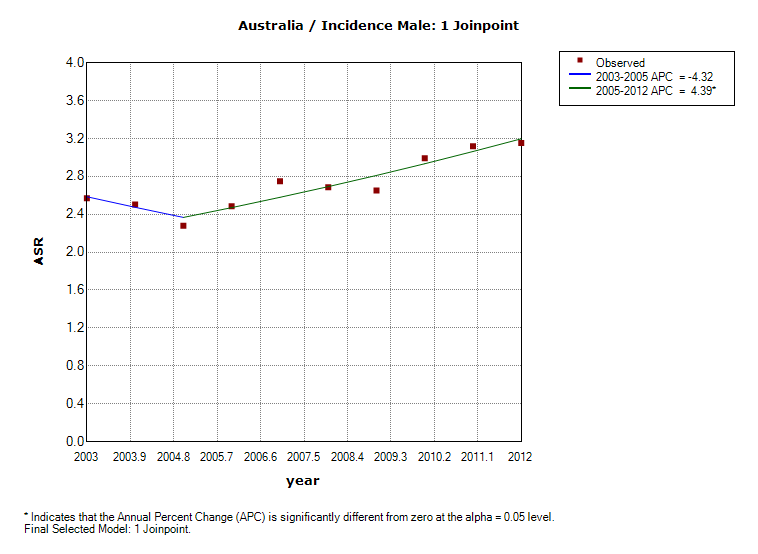 | 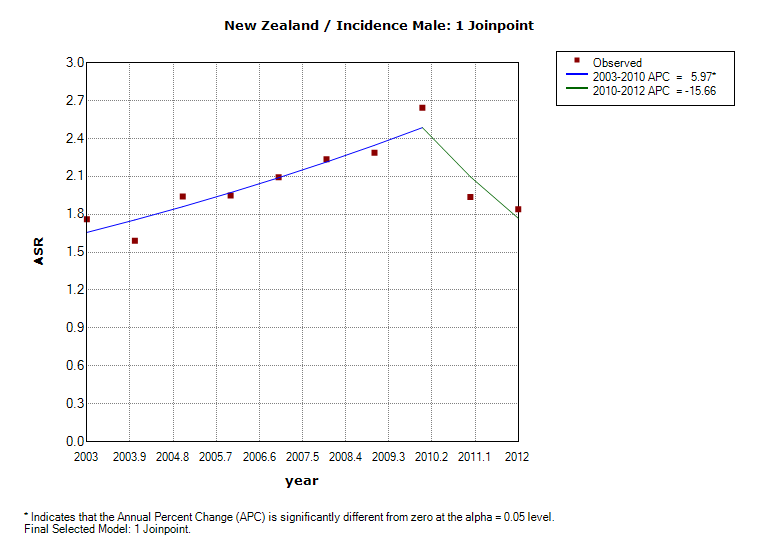 |

| **Northern America** | |
| --- | --- |
| 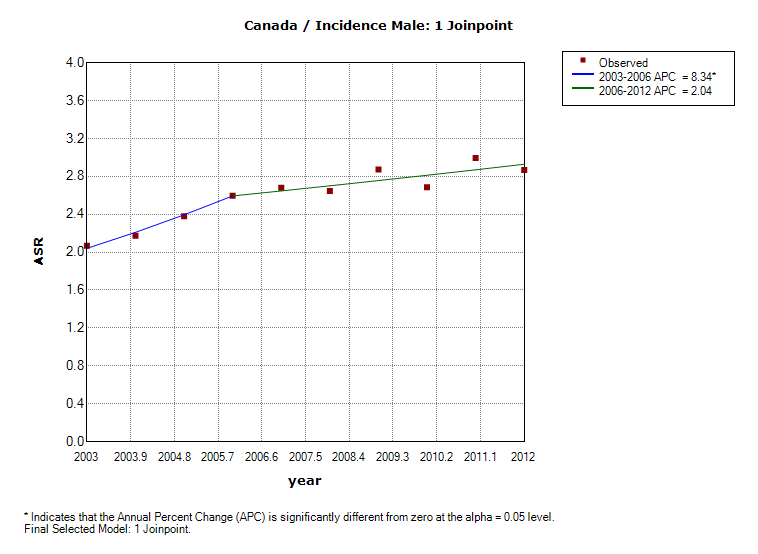 | 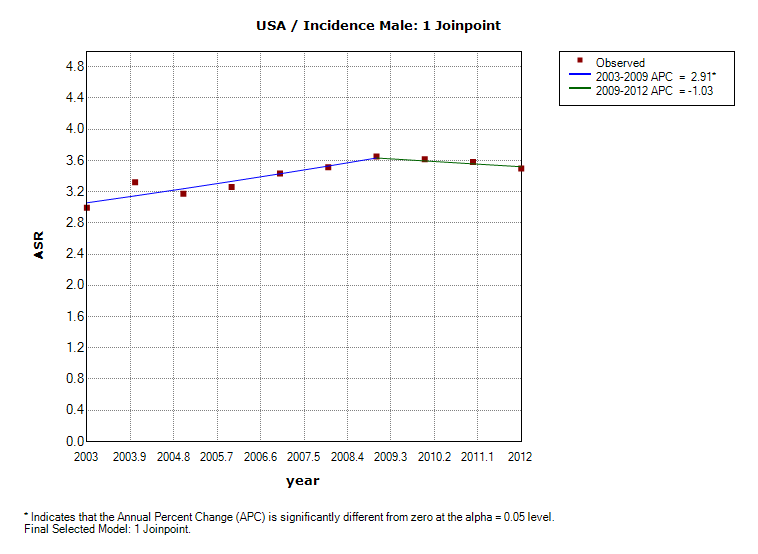 |
| **Southern America** | |
| 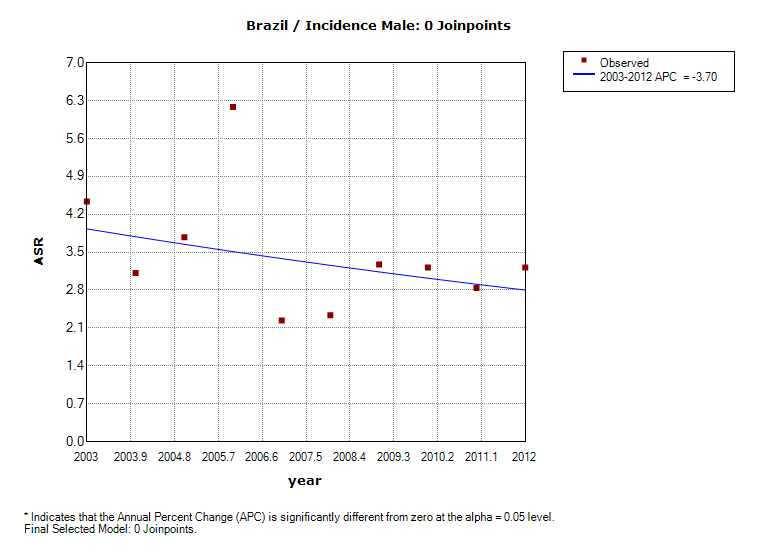 | 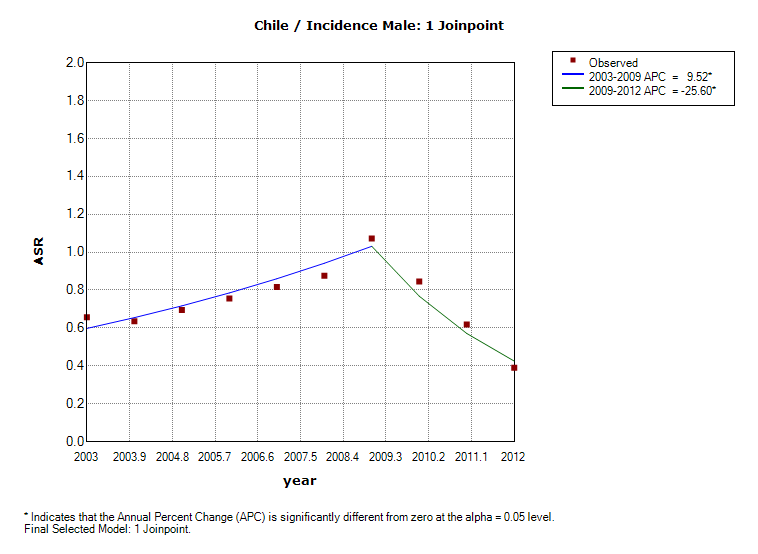 |
| 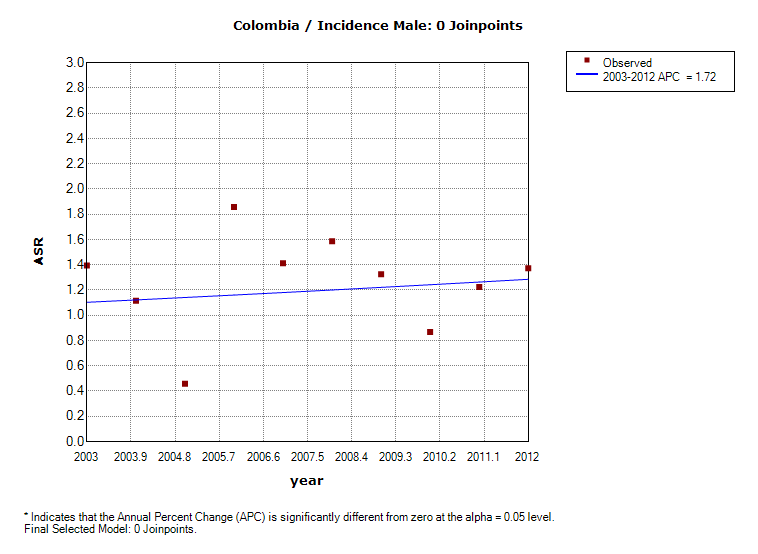 | 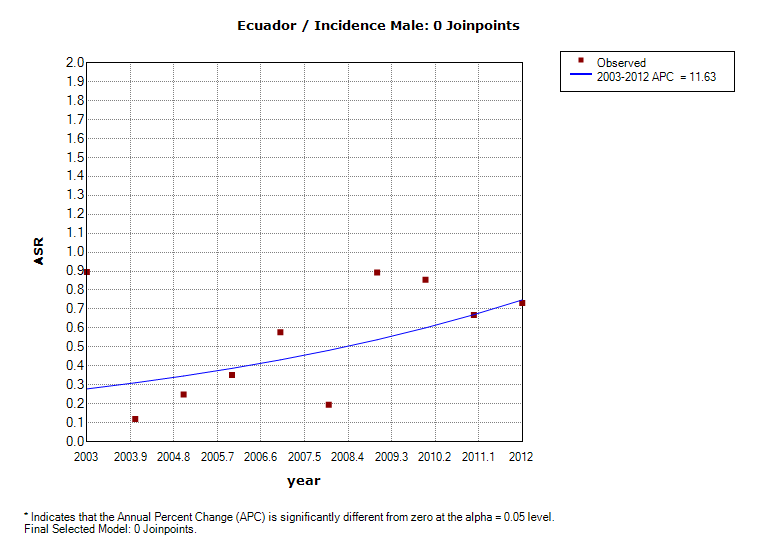 |
| 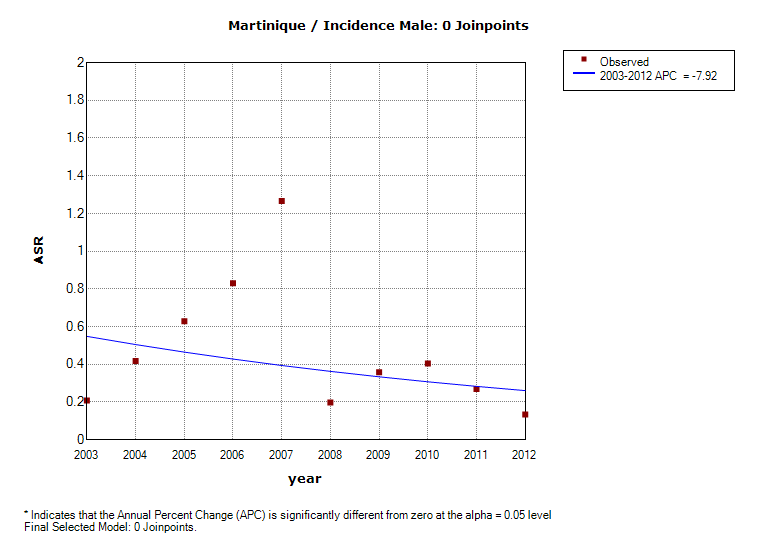 |  |

| **Northern Europe** | |
| --- | --- |
| 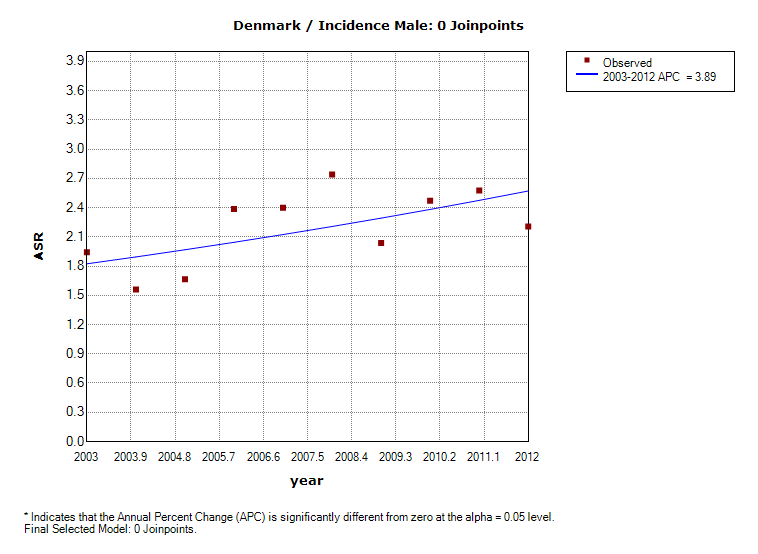 | 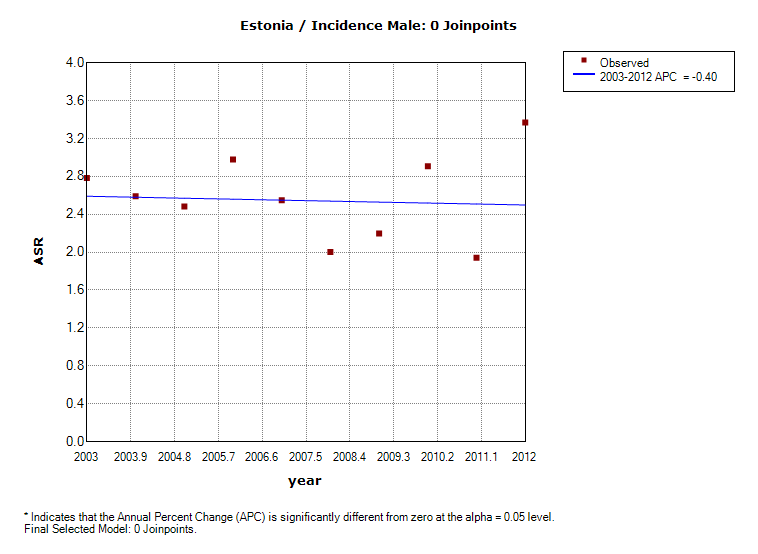 |
| 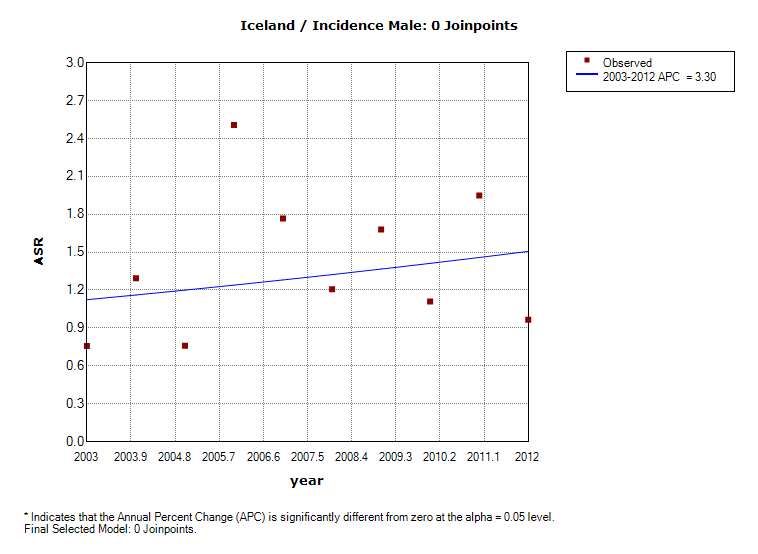 | 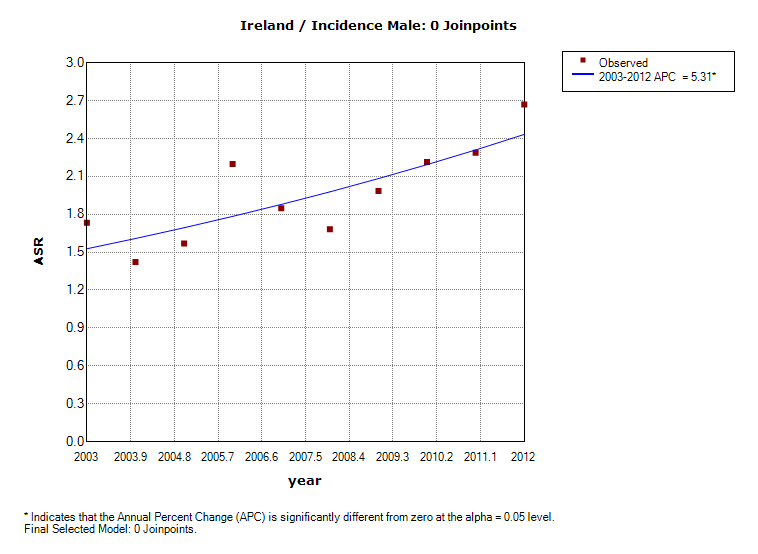 |
| 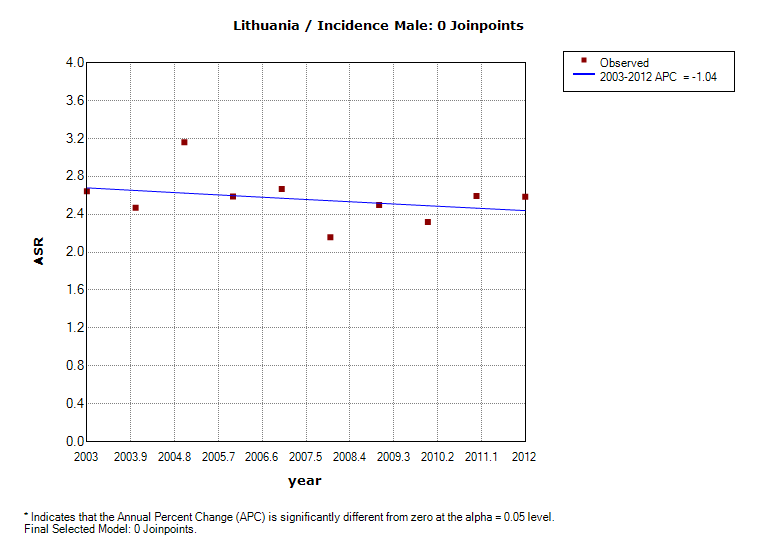 | 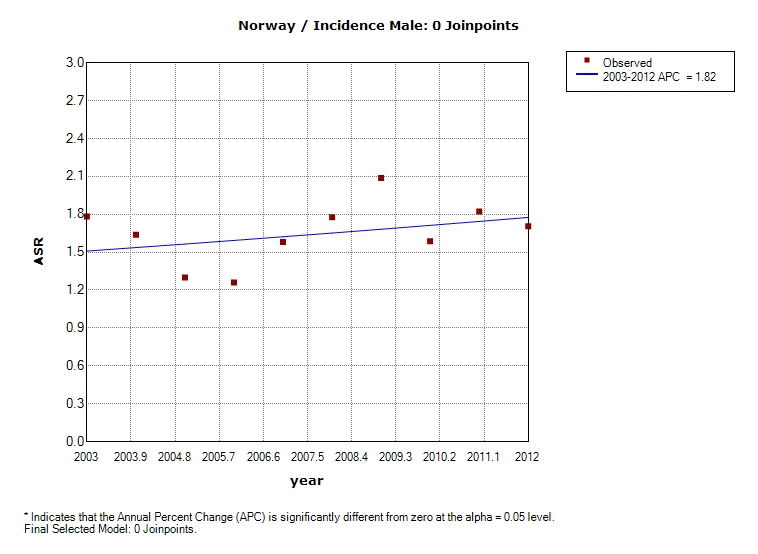 |
| 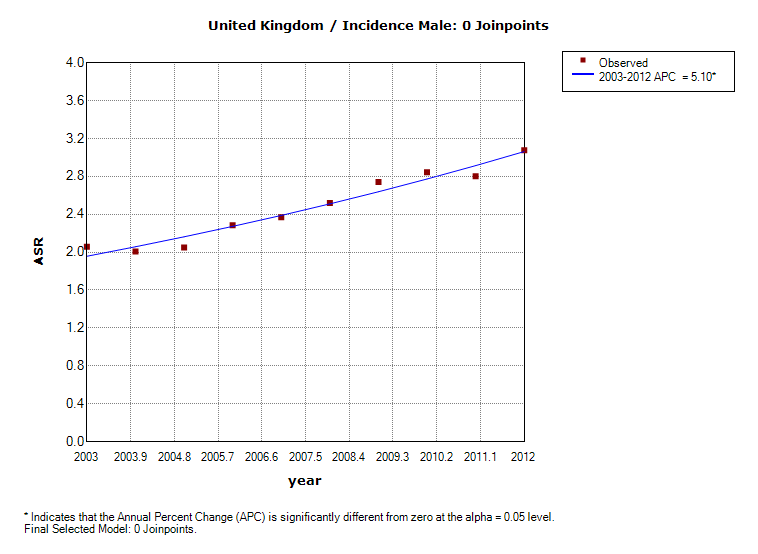 |  |

| **Western Europe** | |
| --- | --- |
| 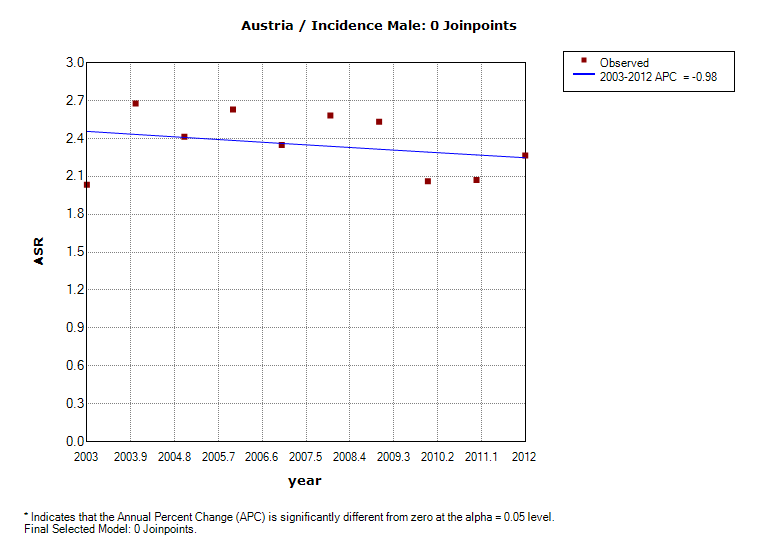 | 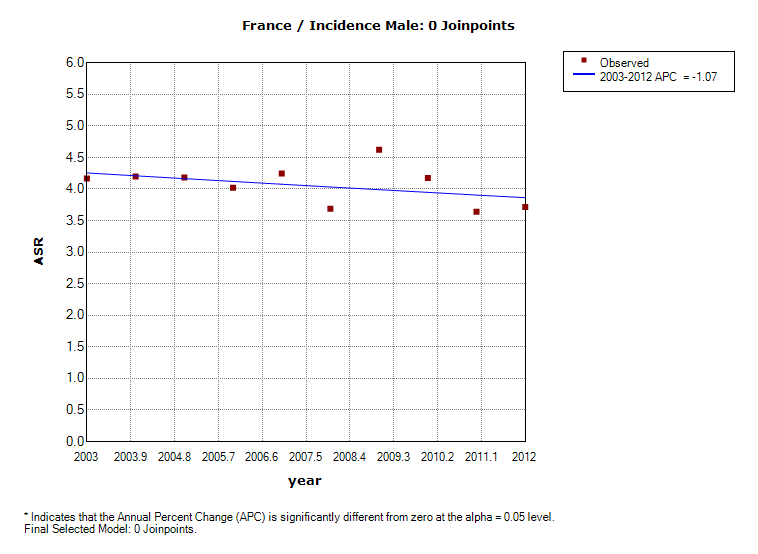 |
| 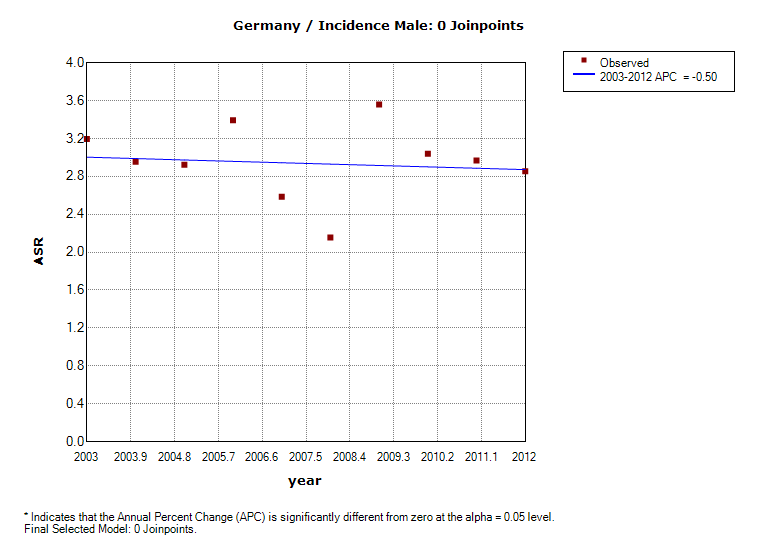 | 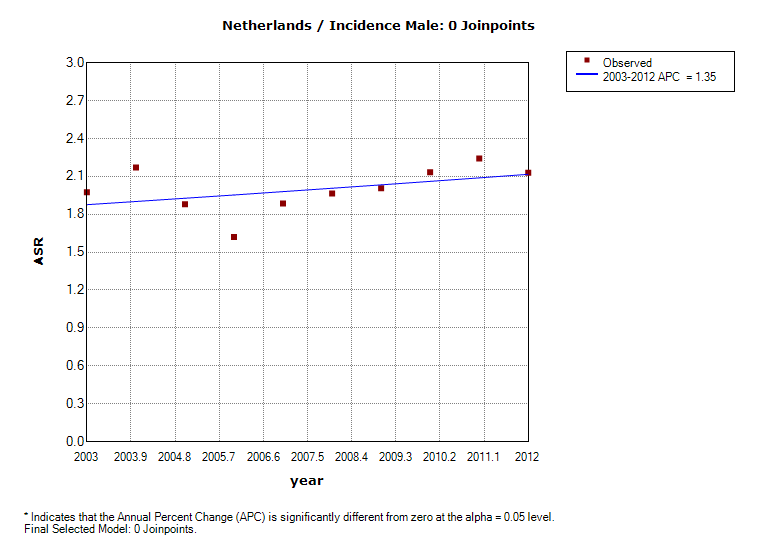 |
| 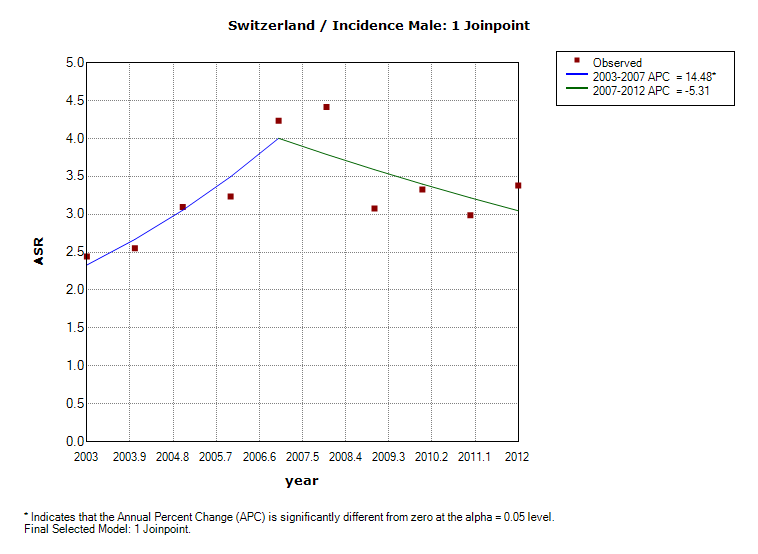 |  |

| **Southern Europe** | |
| --- | --- |
| 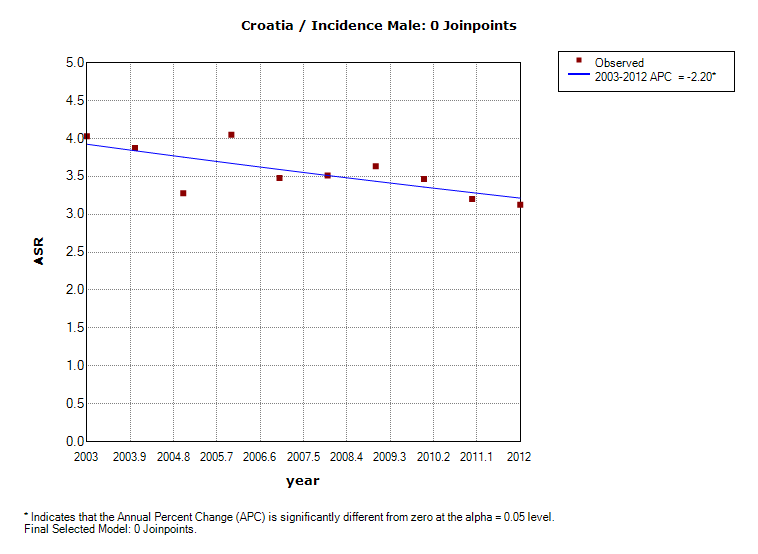 | 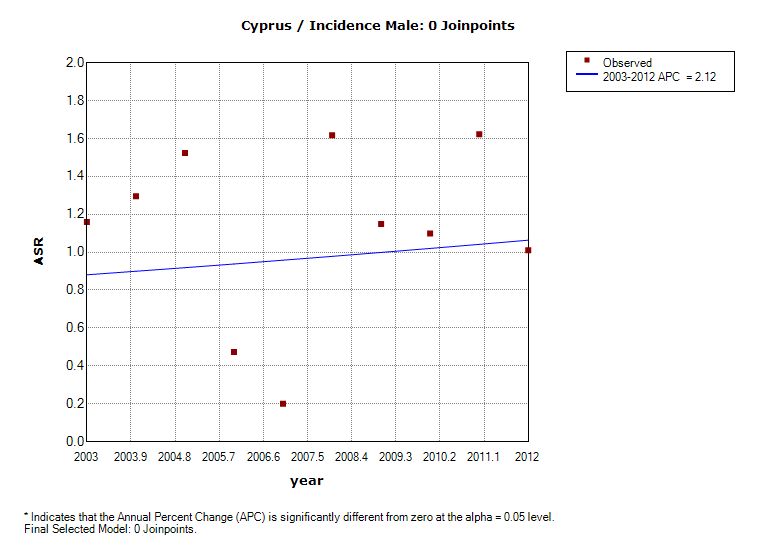 |
| 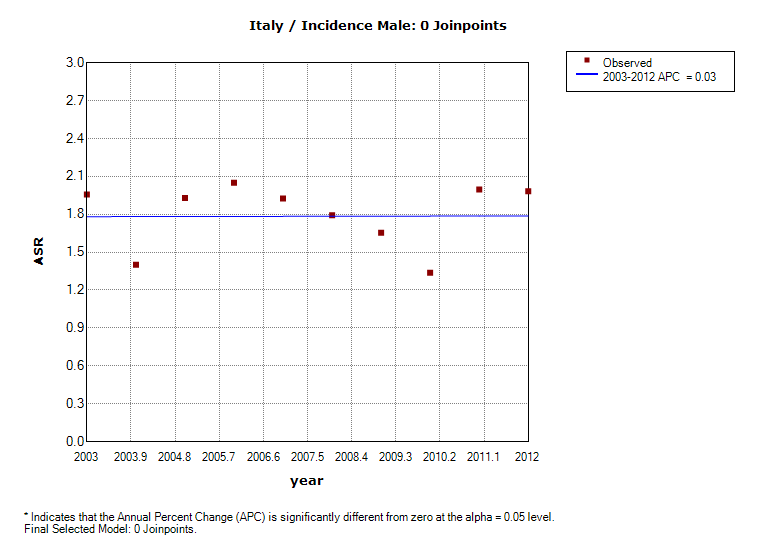 | 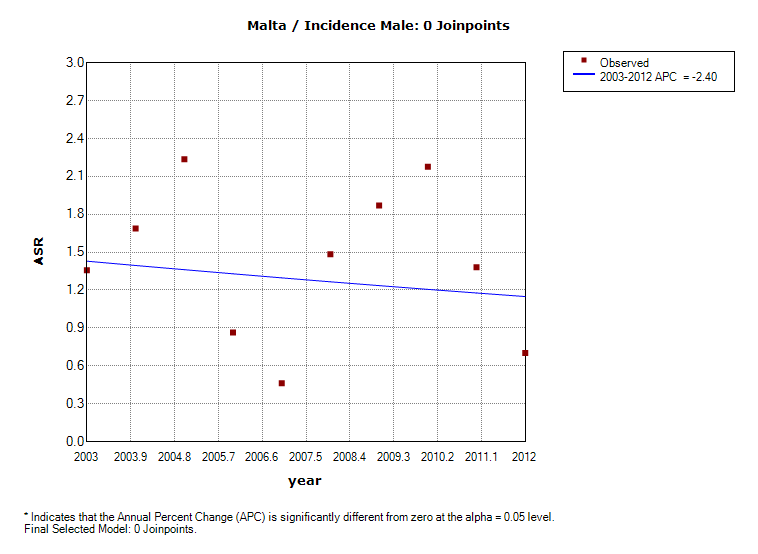 |
| 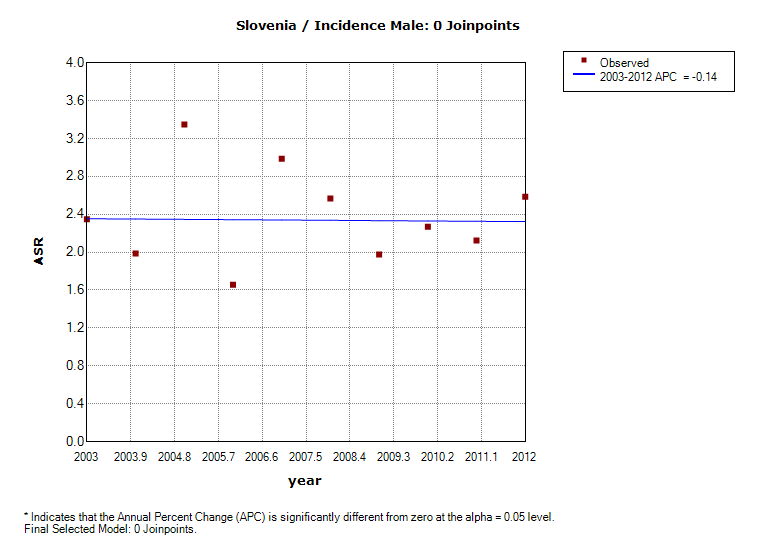 | 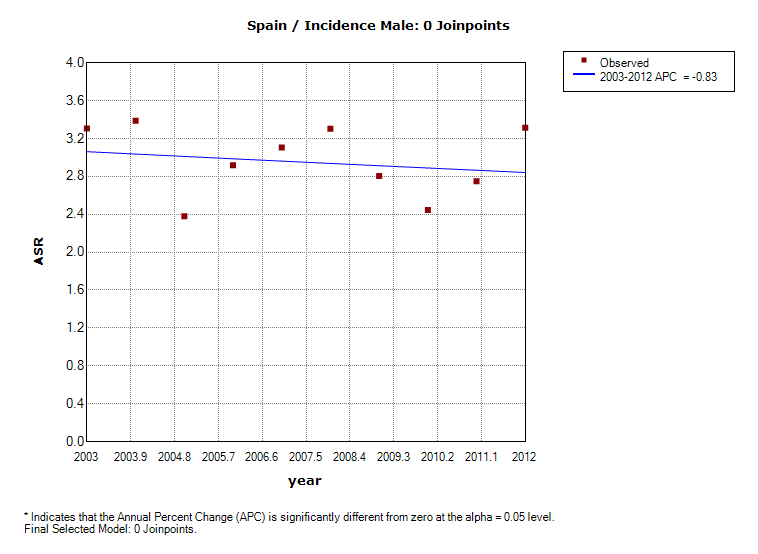 |

| **Eastern Europe** | |
| --- | --- |
| 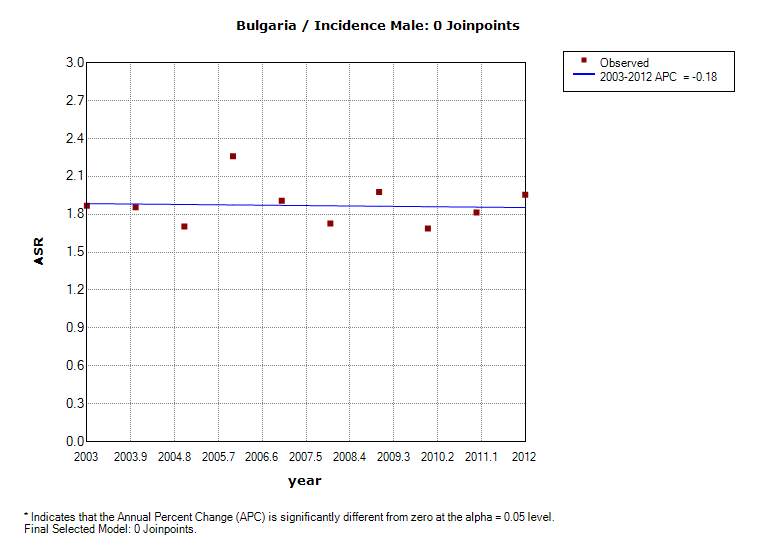 | 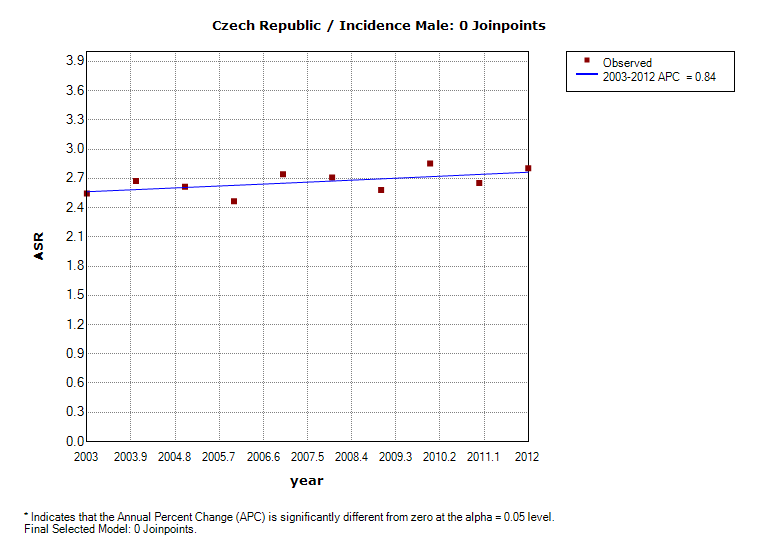 |
| 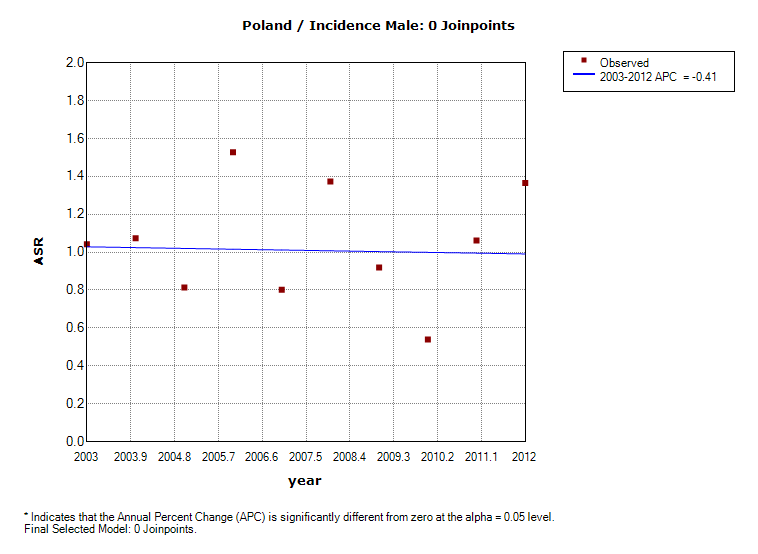 |  |
| **Africa** | |
| 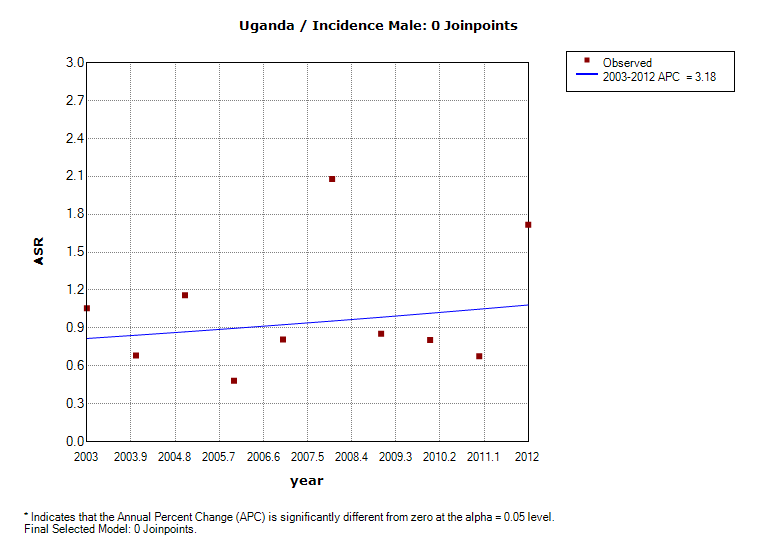 |  |

b) Female

| **Asia** | |
| --- | --- |
| 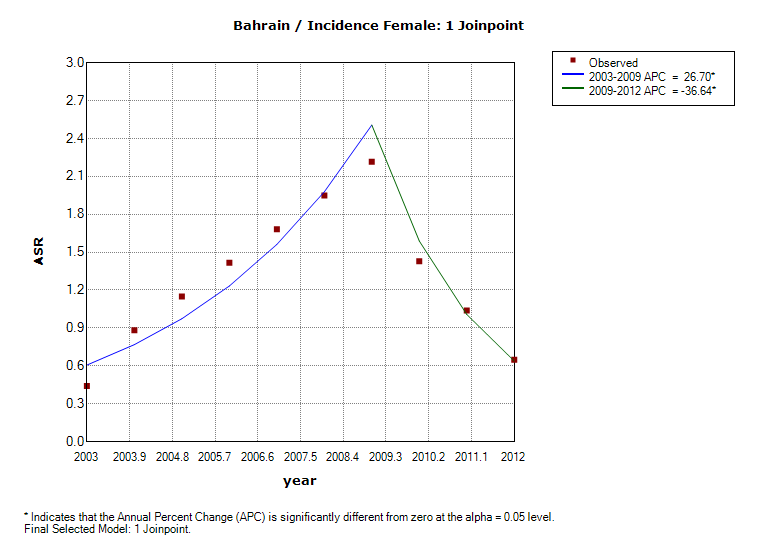 | 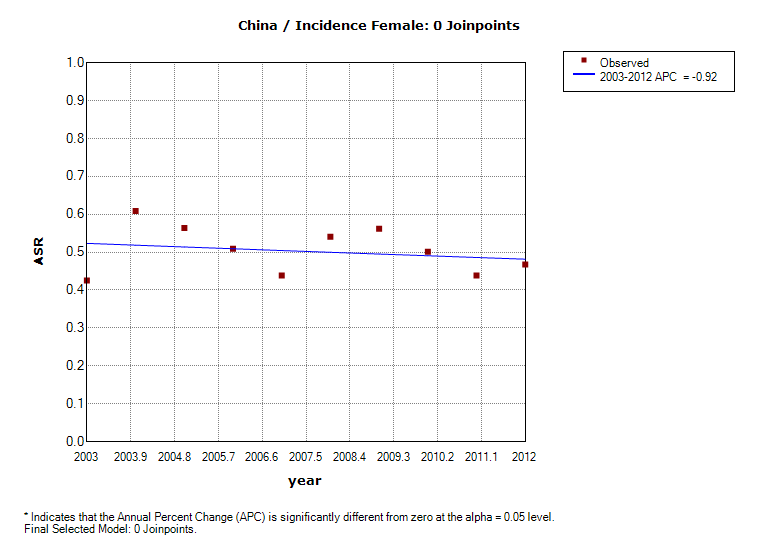 |
| 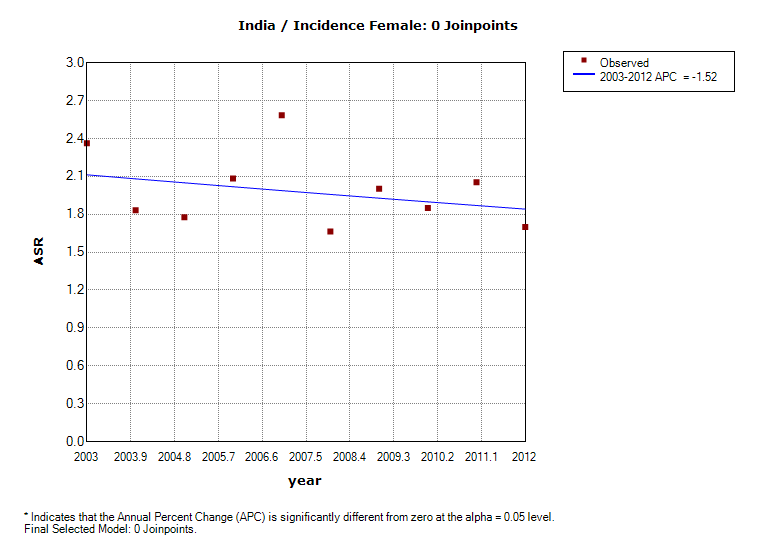 | 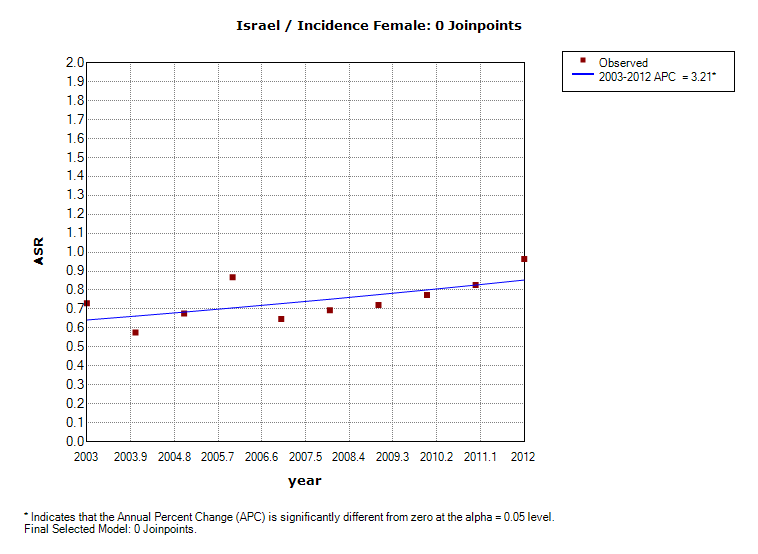 |
| 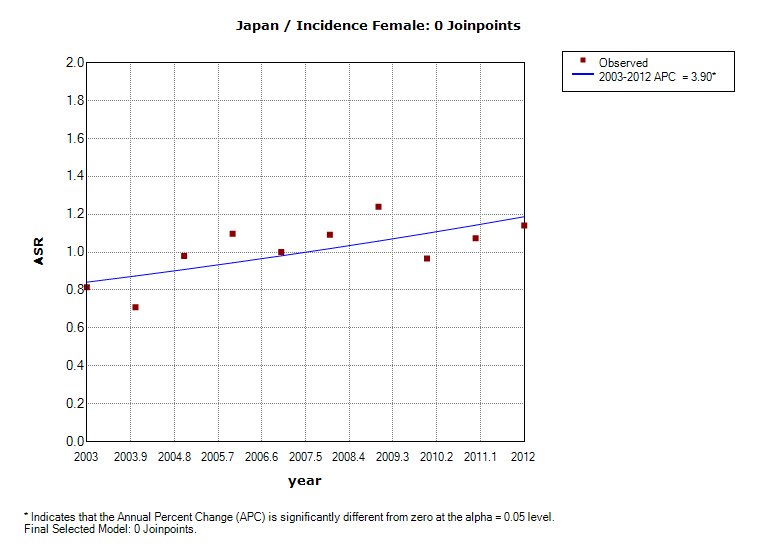 | 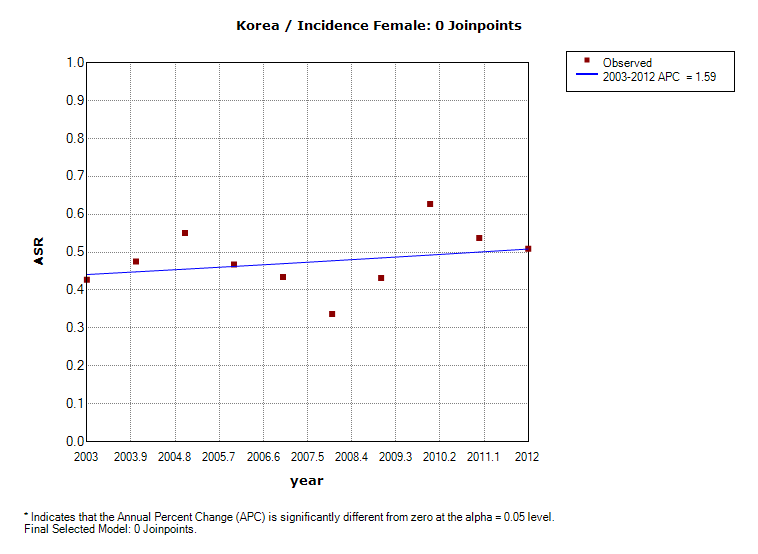 |
| 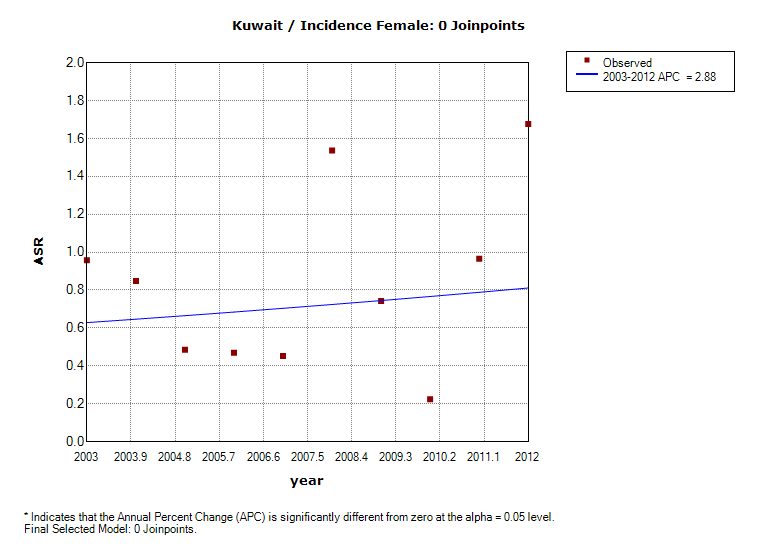 | 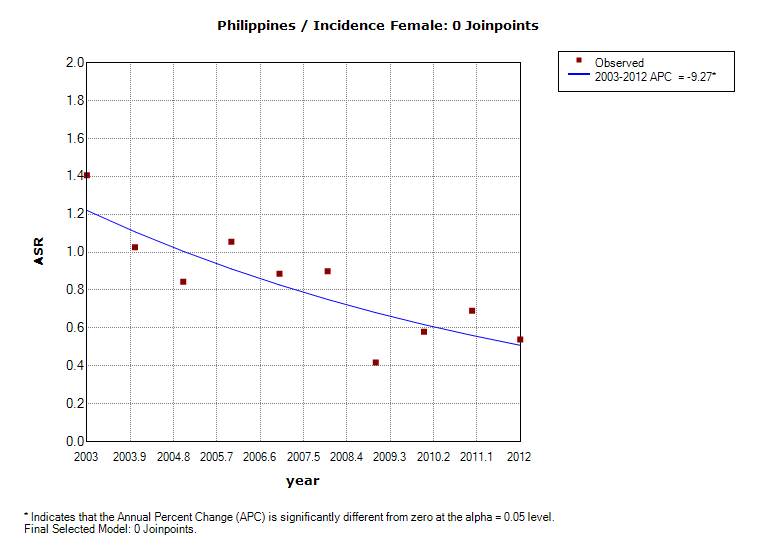 |
| 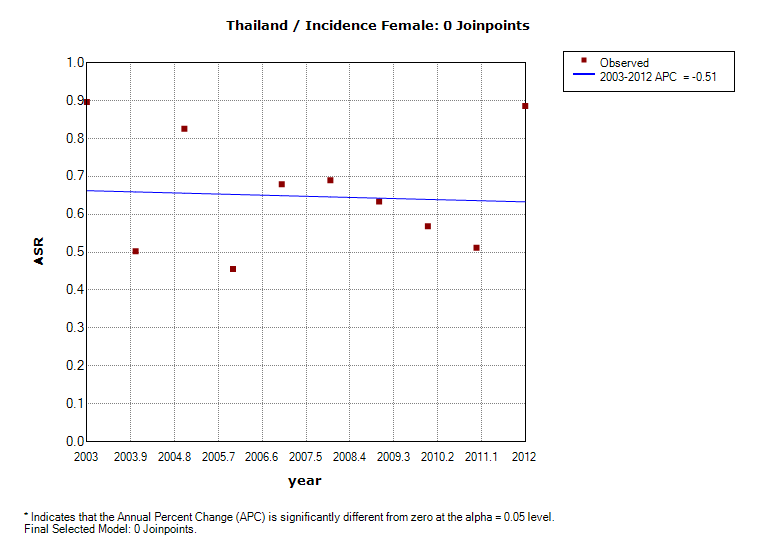 | 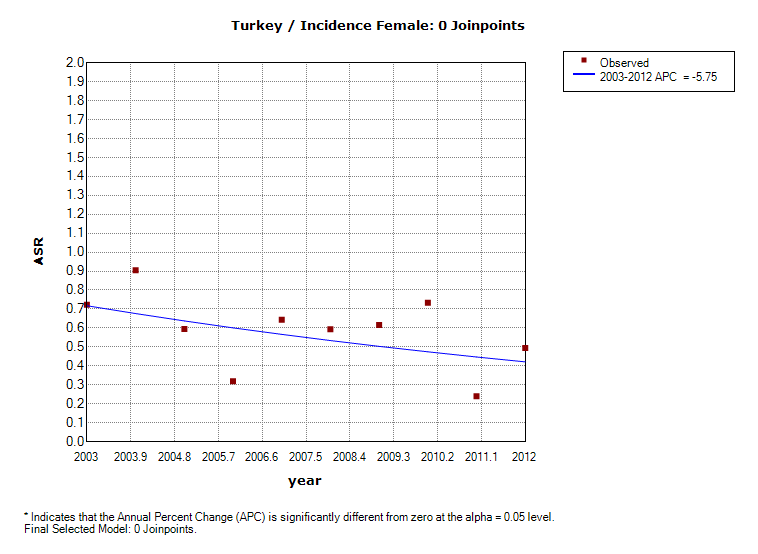 |
| **Oceania** | |
| 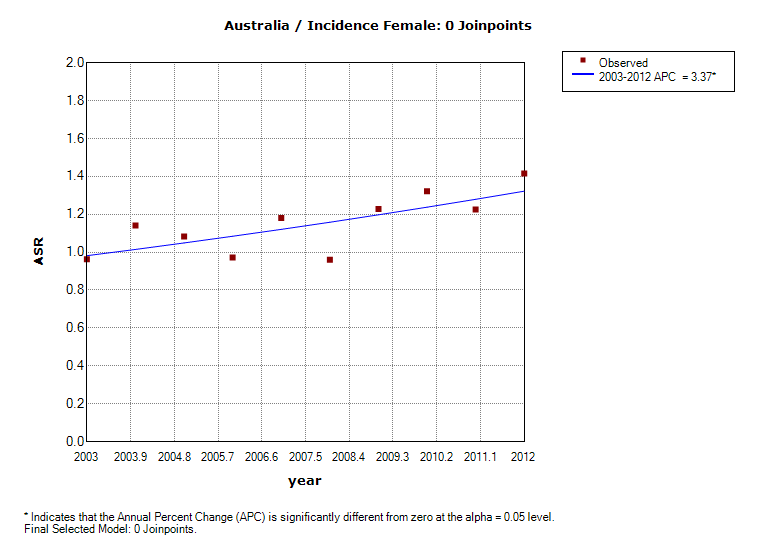 | 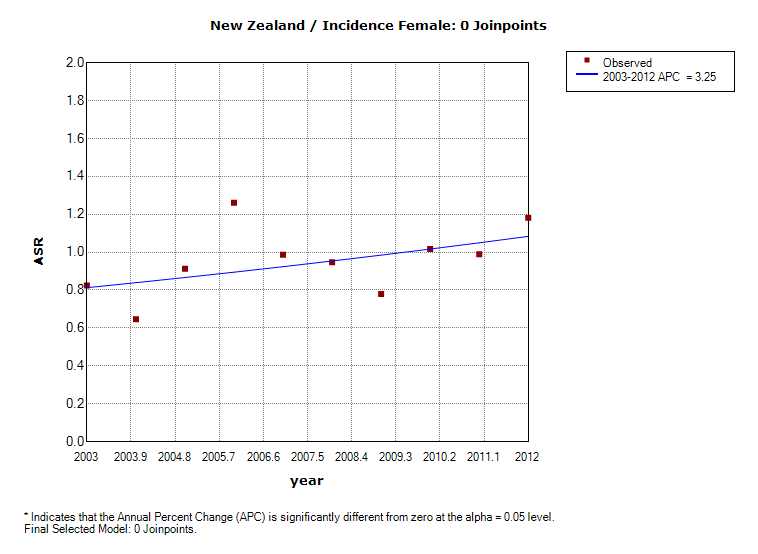 |

| **Northern America** | | |
| --- | --- | --- |
| 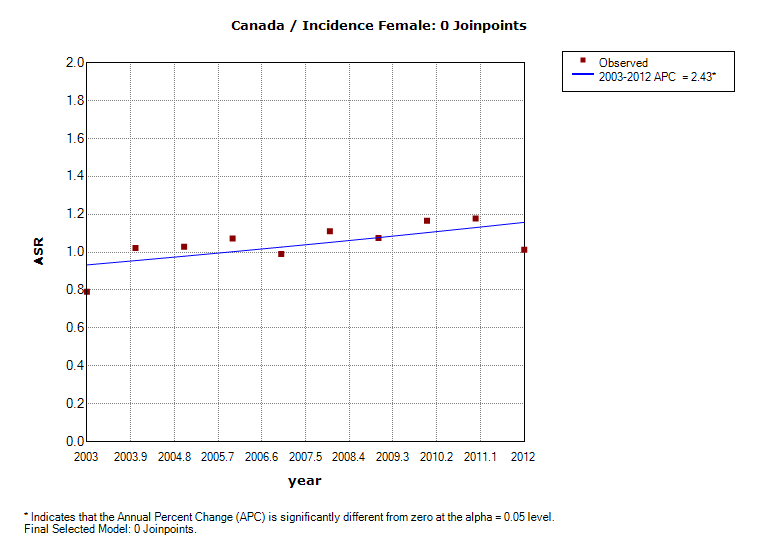 | 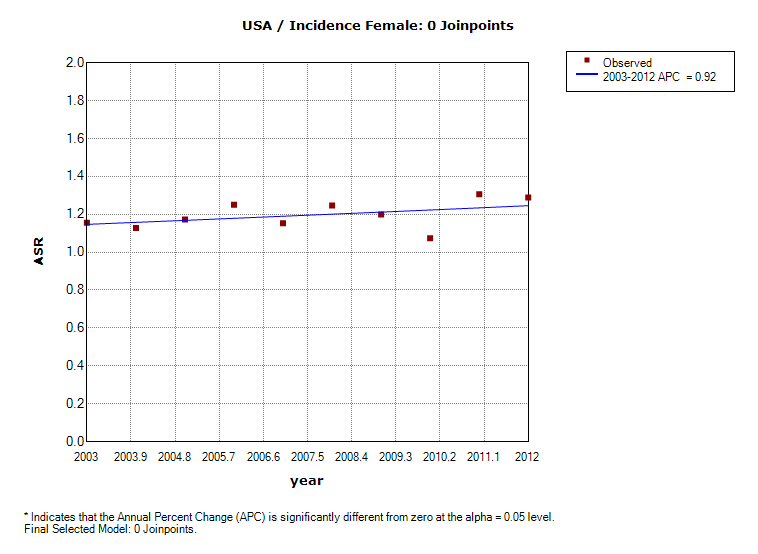 | |
| **Southern America** | | |
| 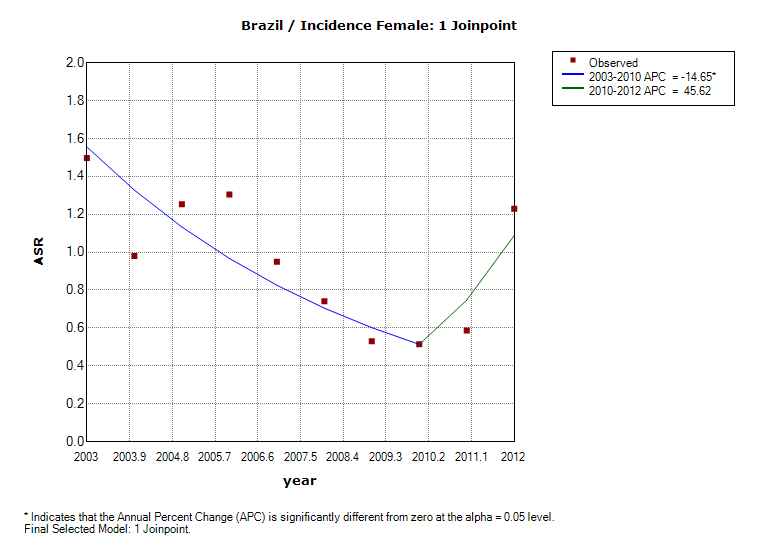 | | 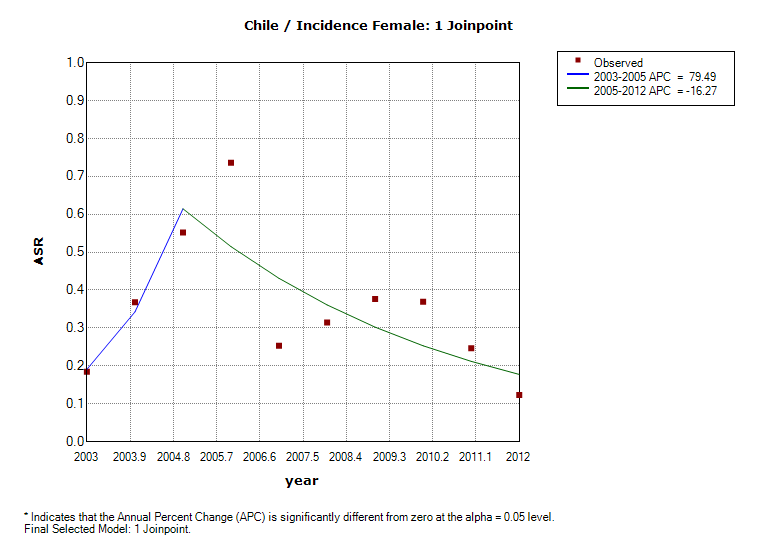 |
| 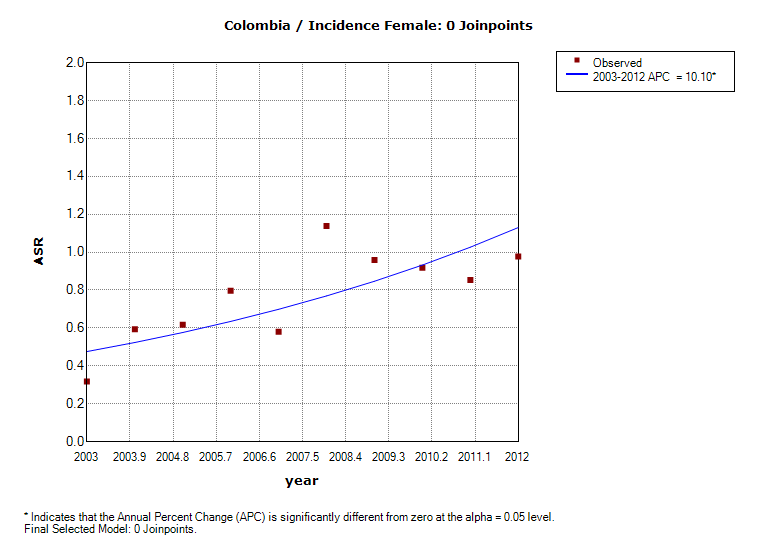 | | 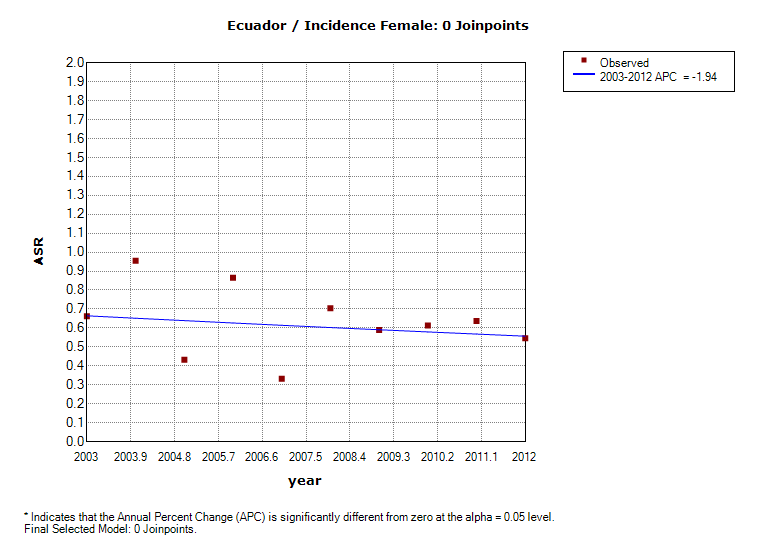 |
| 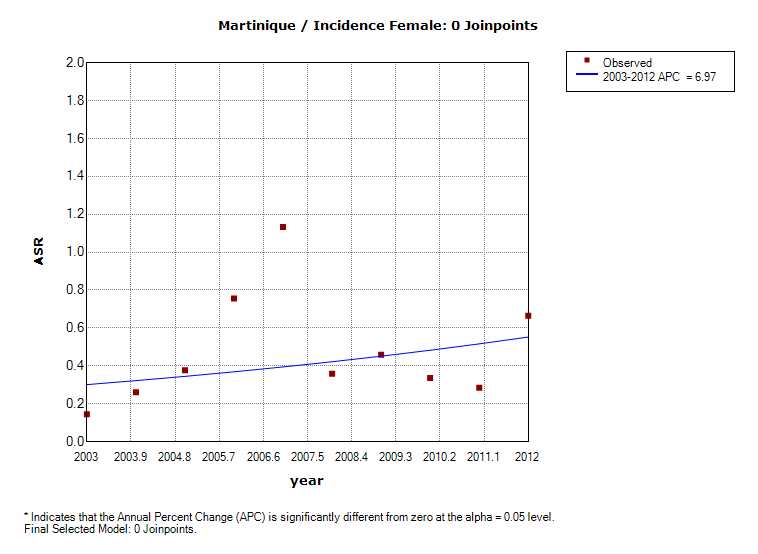 | |  |

| **Northern Europe** | |
| --- | --- |
| 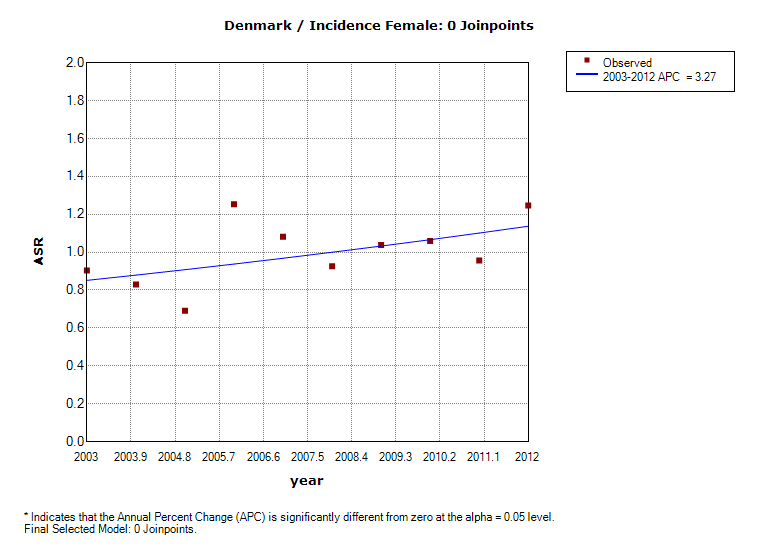 | 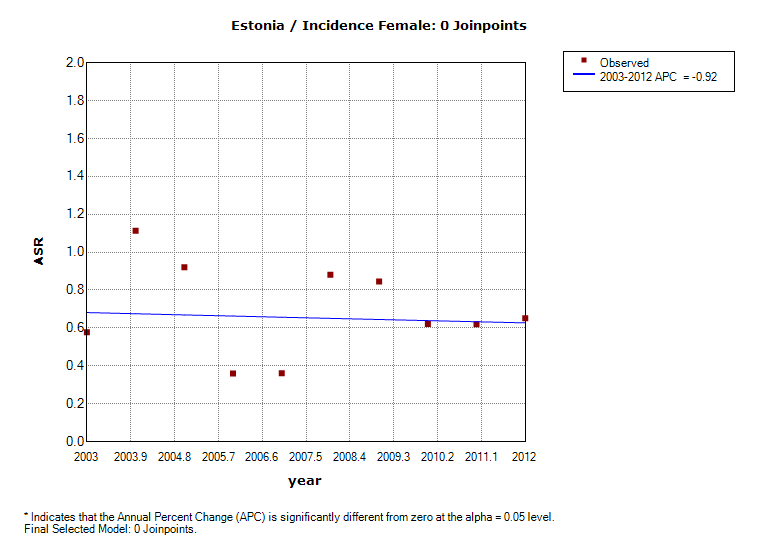 |
| 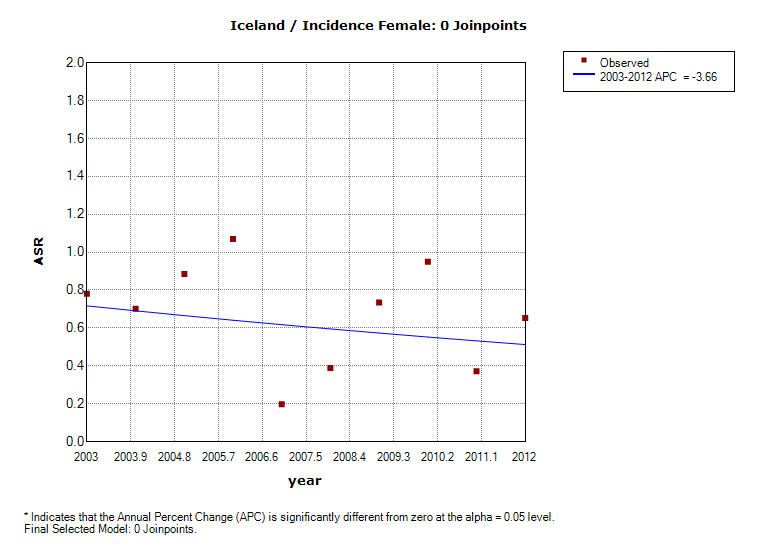 | 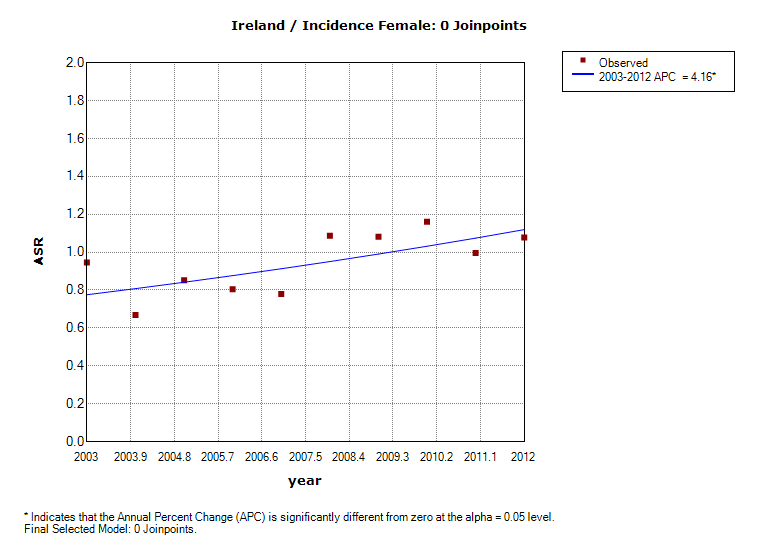 |
| 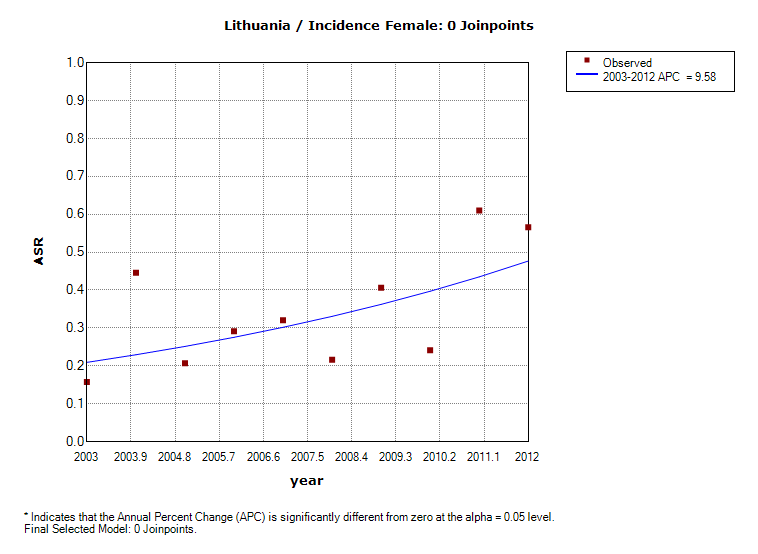 | 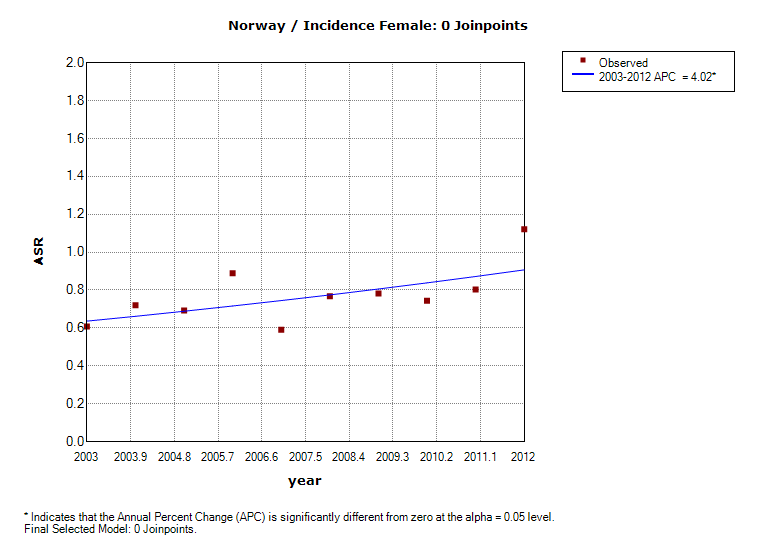 |
| 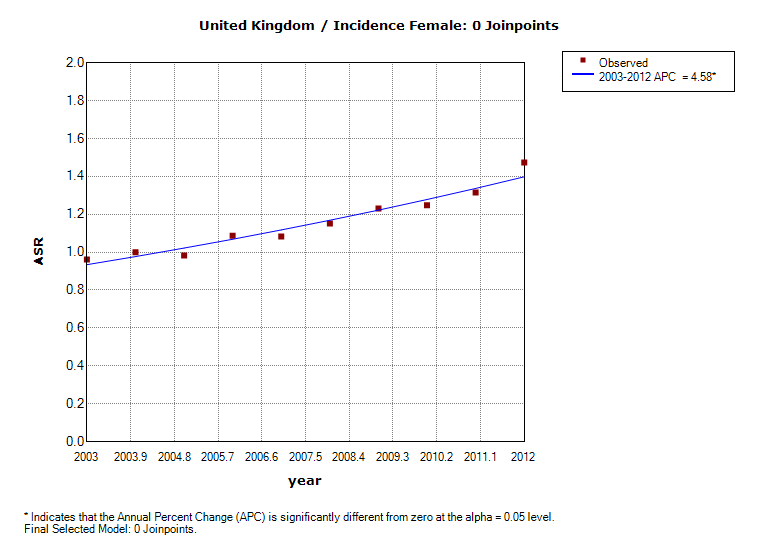 |  |
| **Western Europe** | |
| 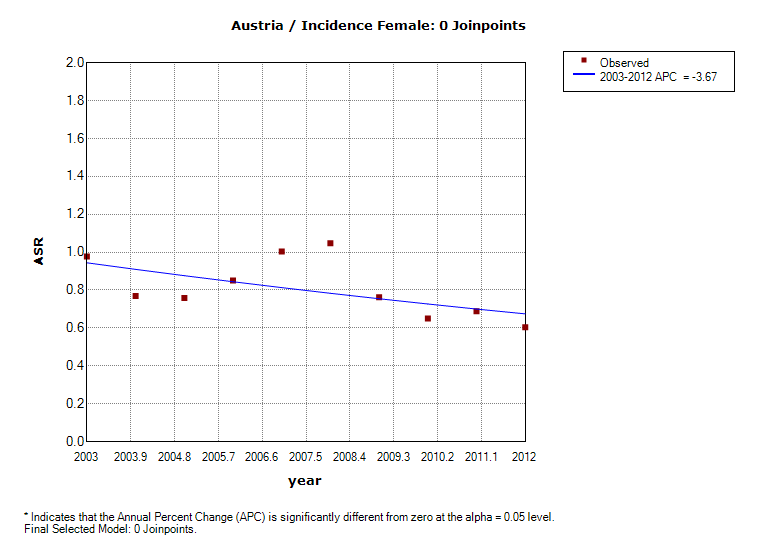 | 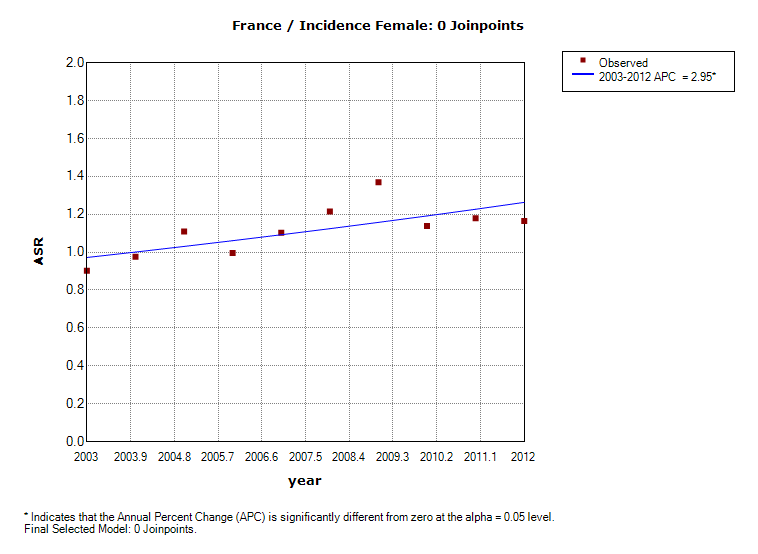 |
| 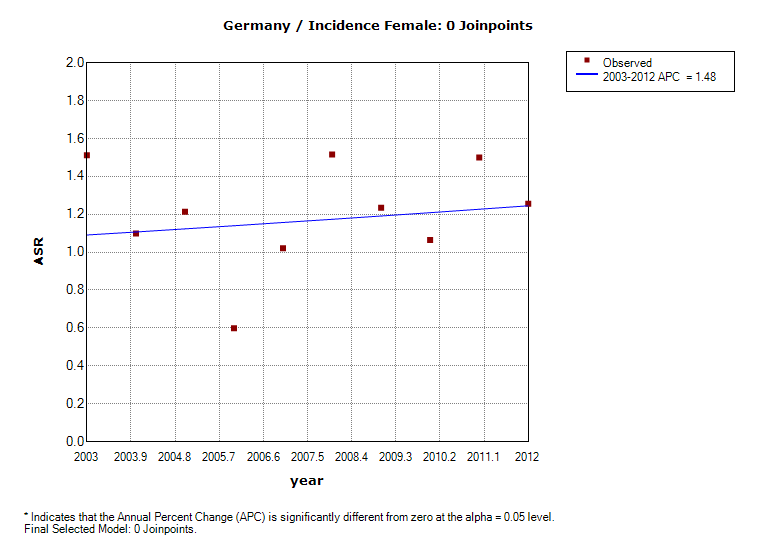 | 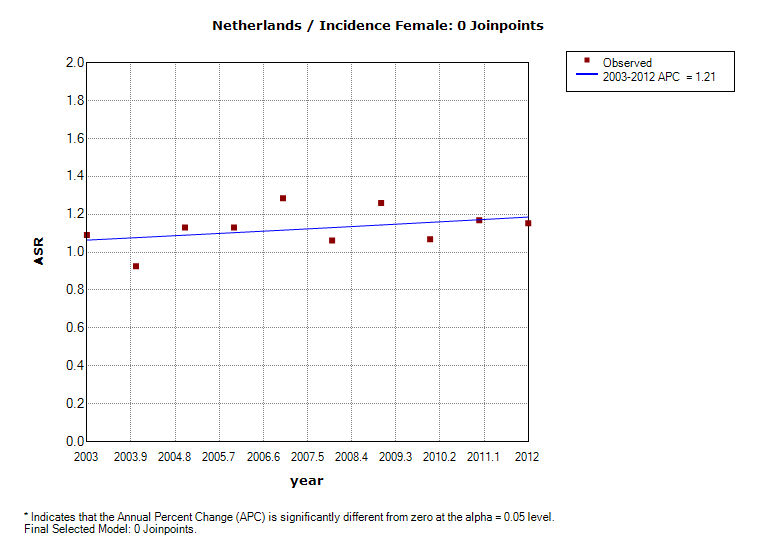 |
| 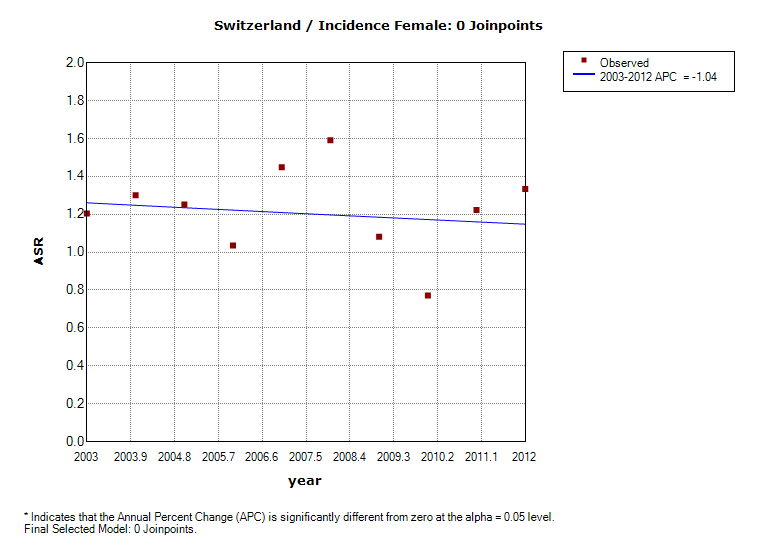 |  |

| **Southern Europe** | |
| --- | --- |
| 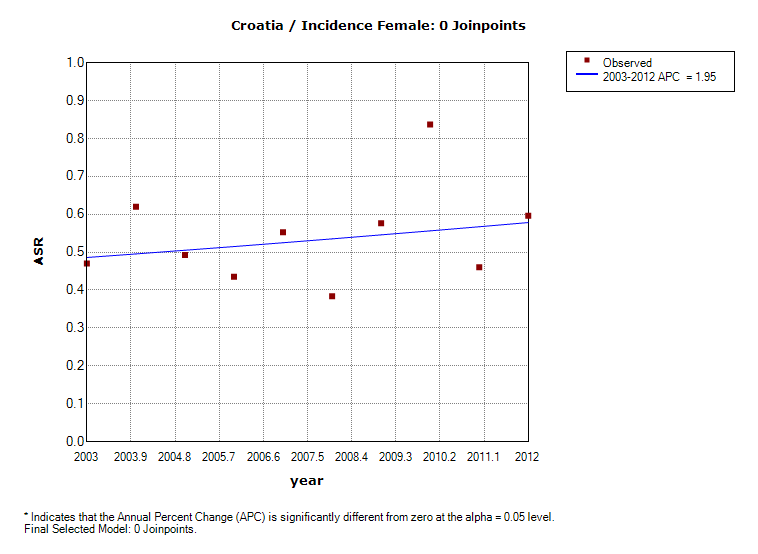 | 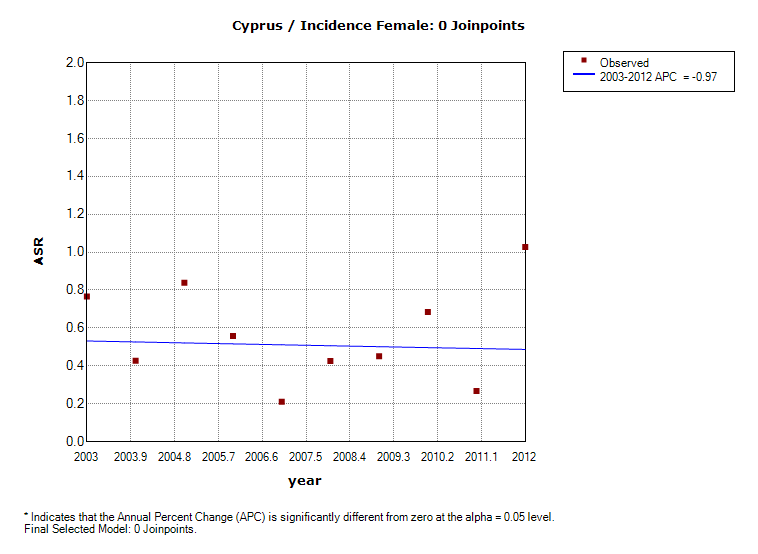 |
| 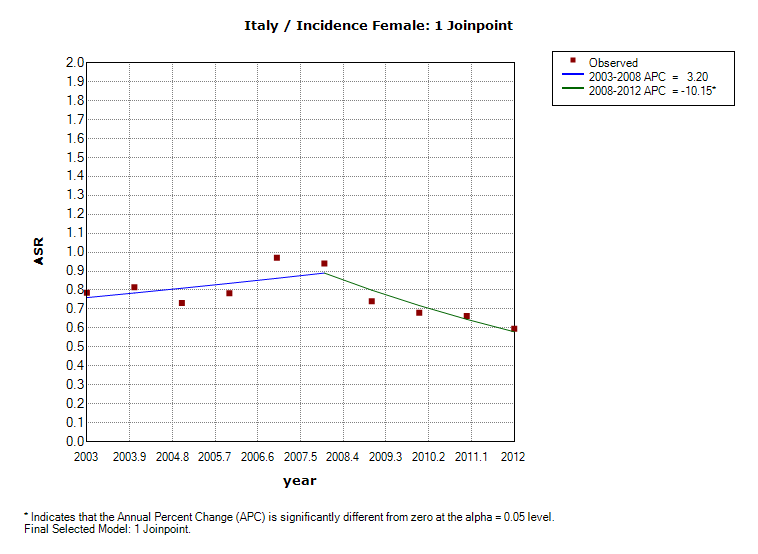 | 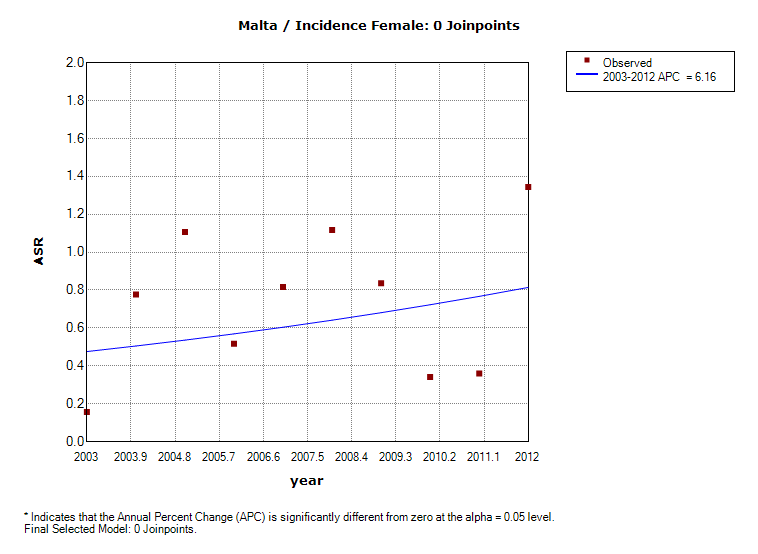 |
| 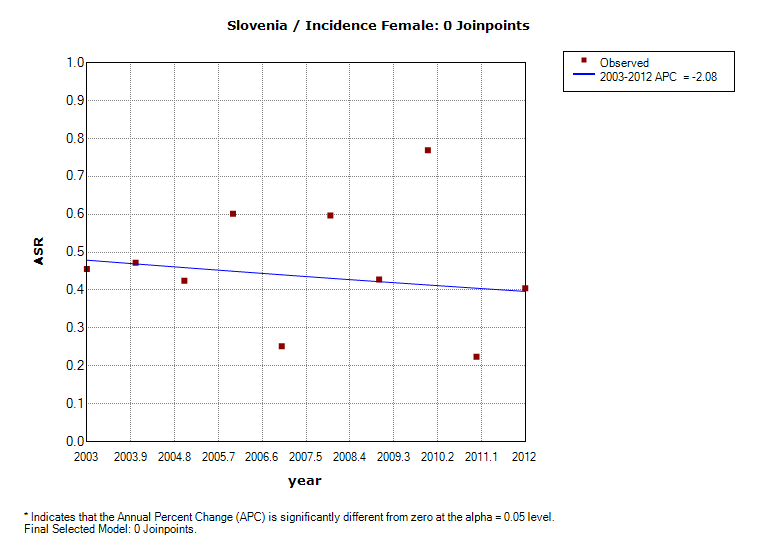 | 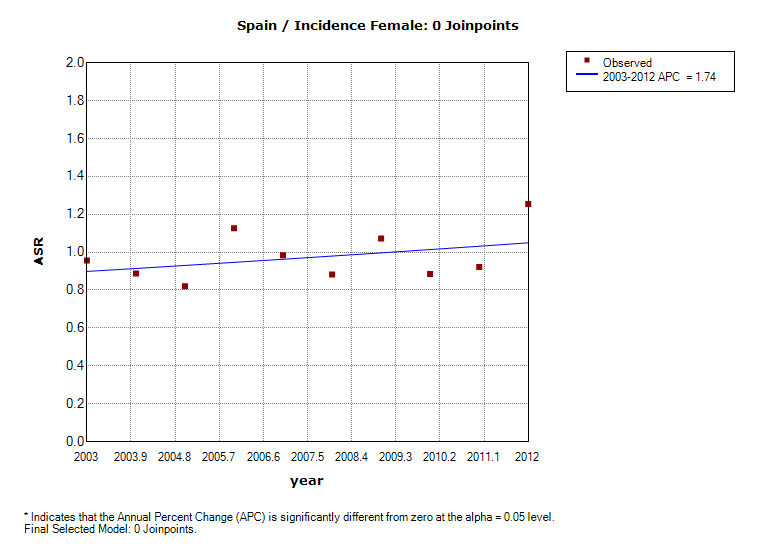 |

| **Eastern Europe** | | |
| --- | --- | --- |
| 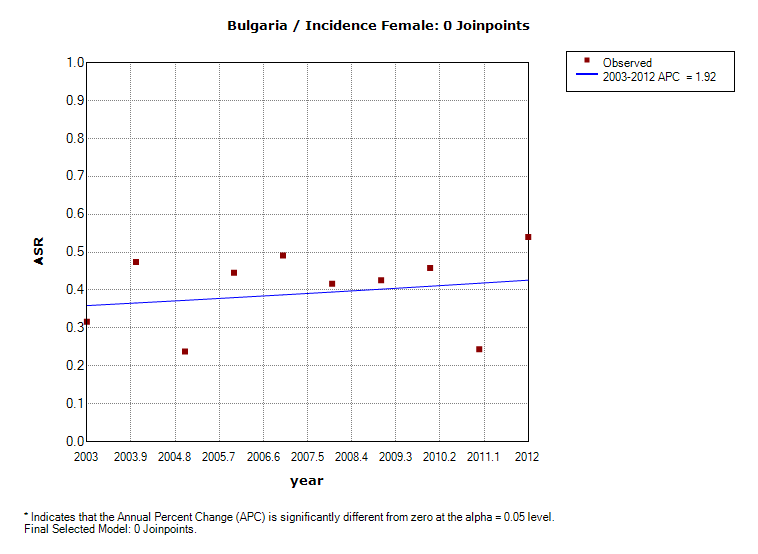 | 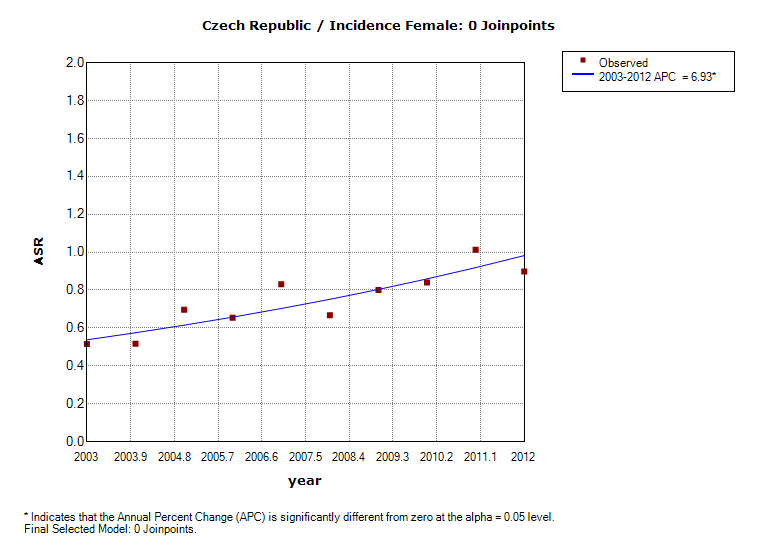 | |
| 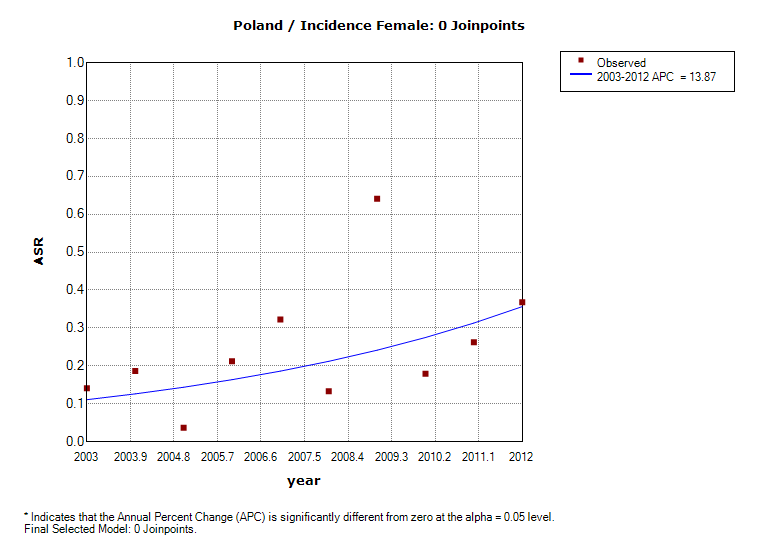 |  | |
| **Africa** | | |
| 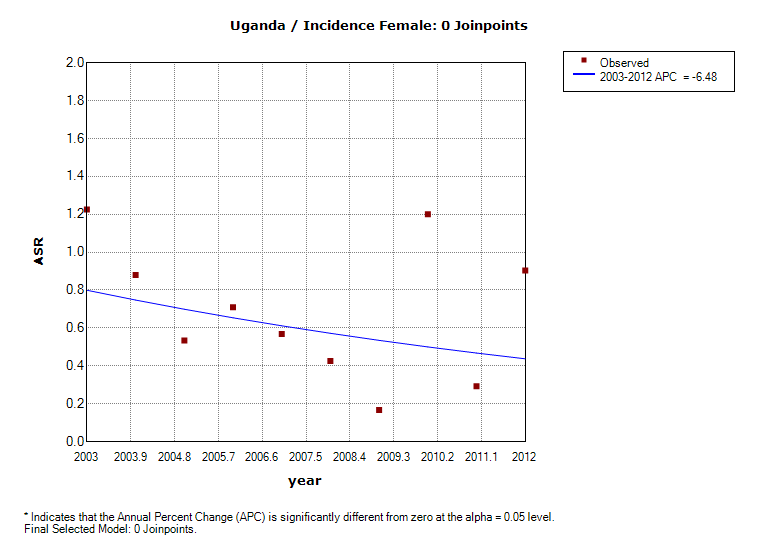 | |  |

c) Both

| **Asia** | |
| --- | --- |
| 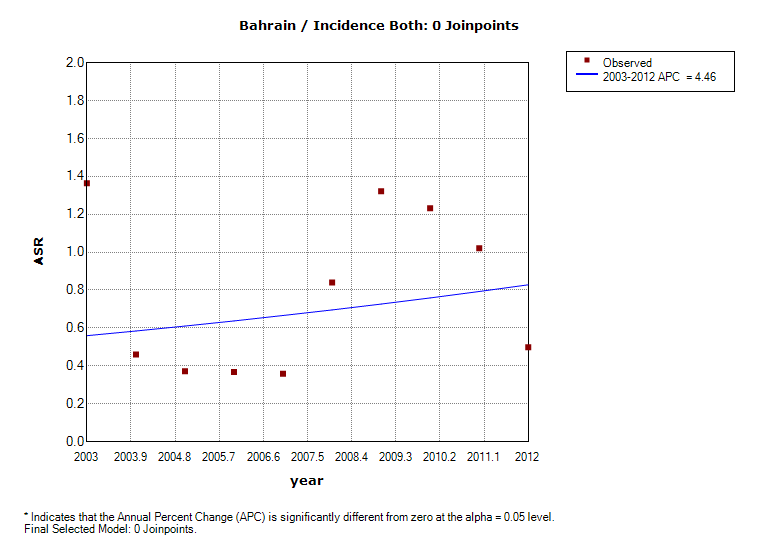 | 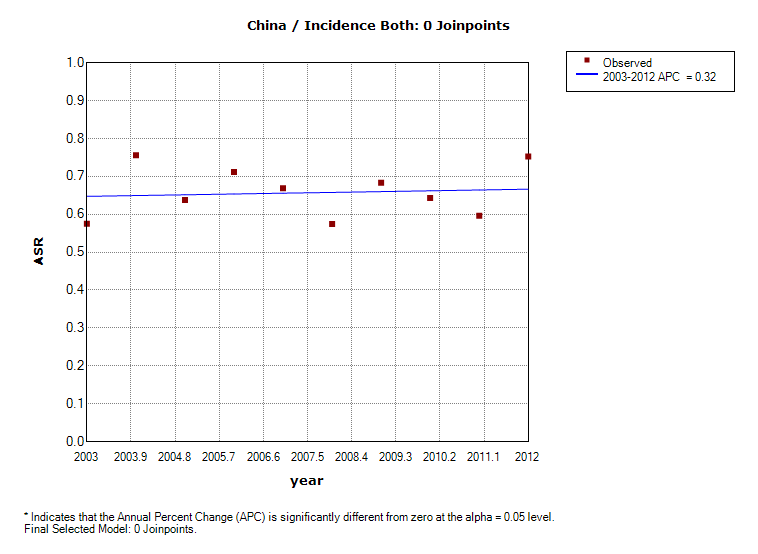 |
| 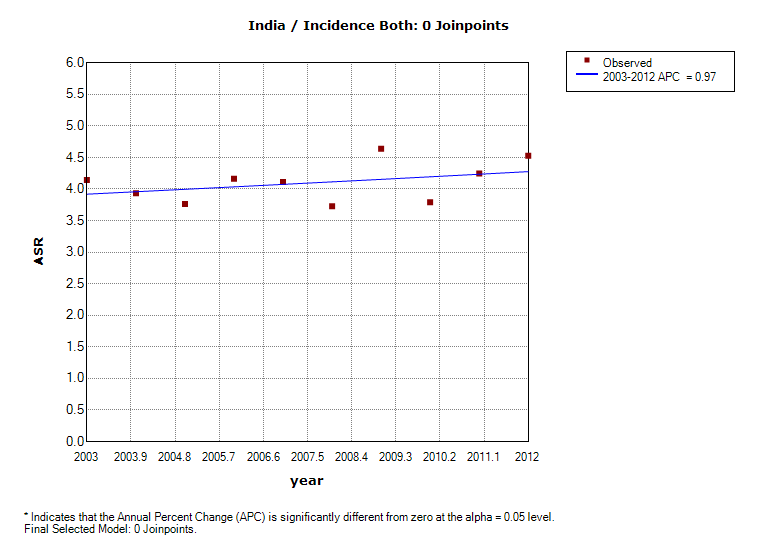 | 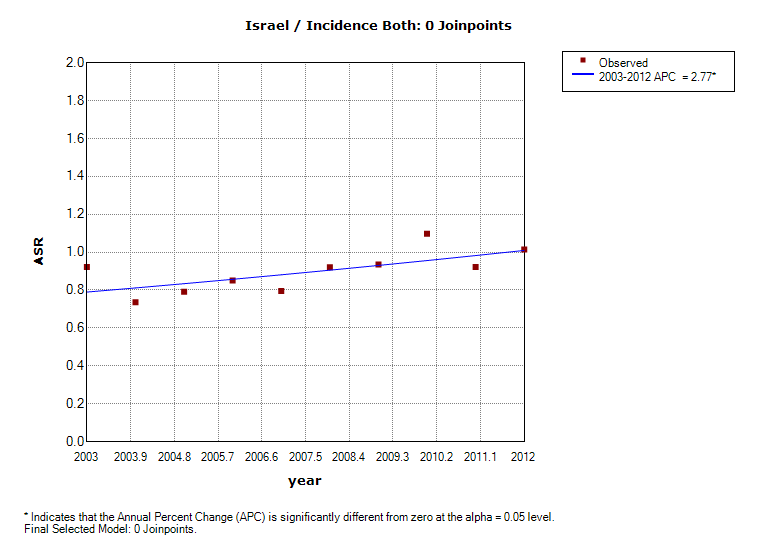 |
| 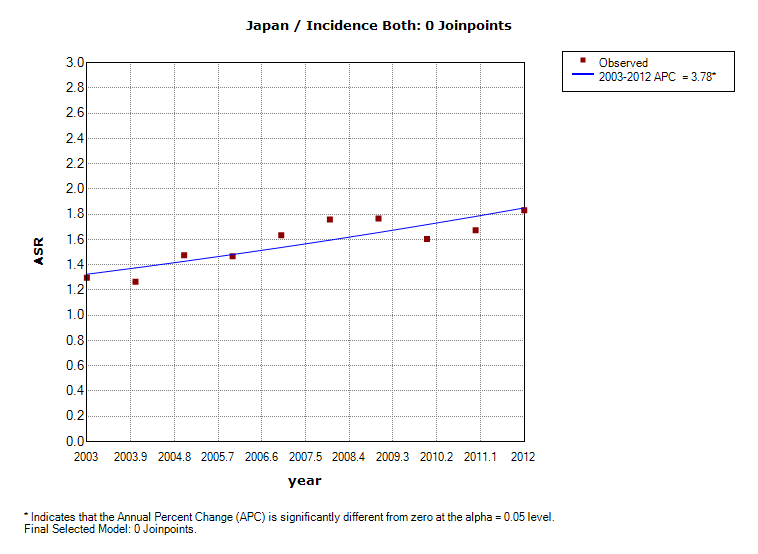 | 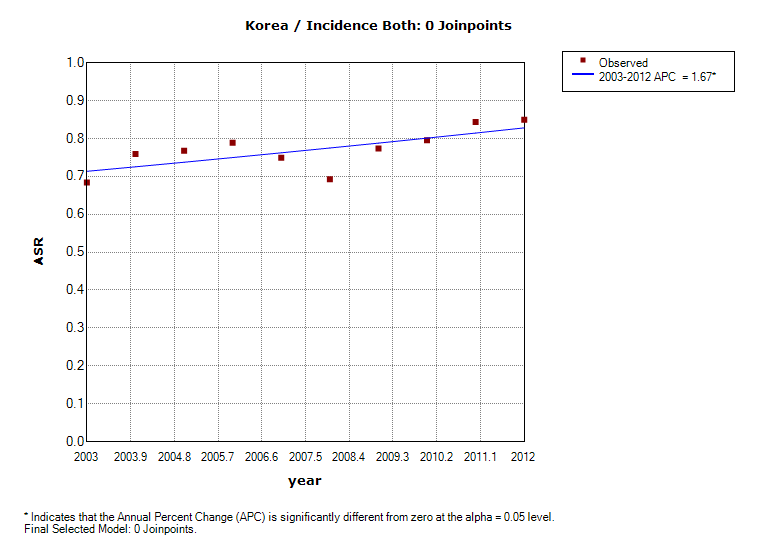 |
| 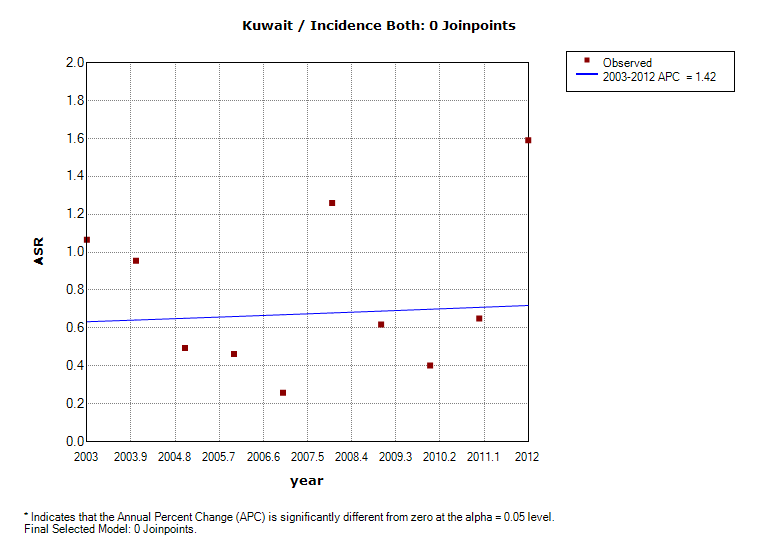 | 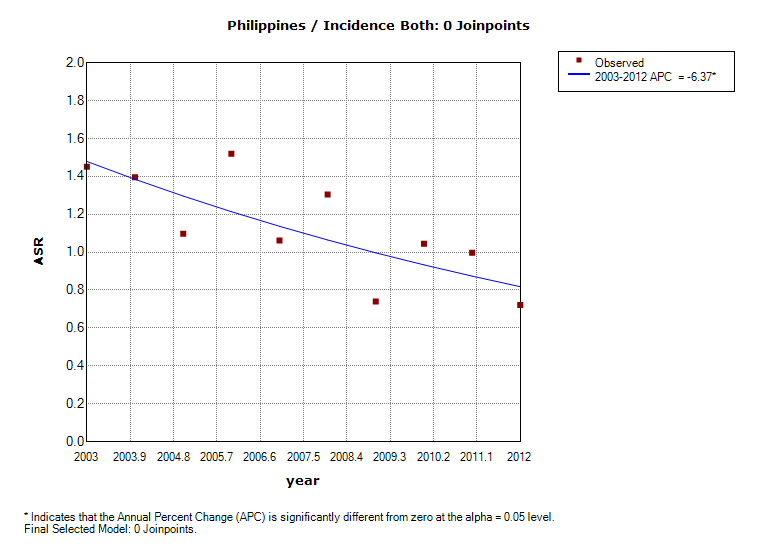 |
| 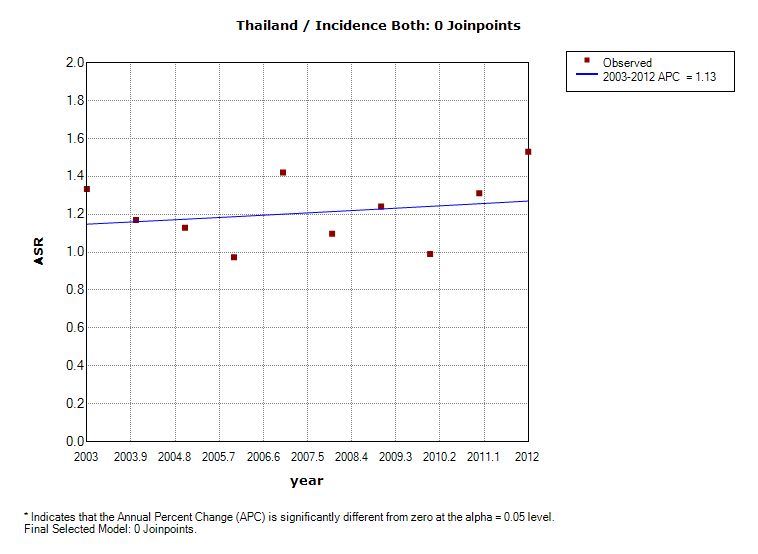 | 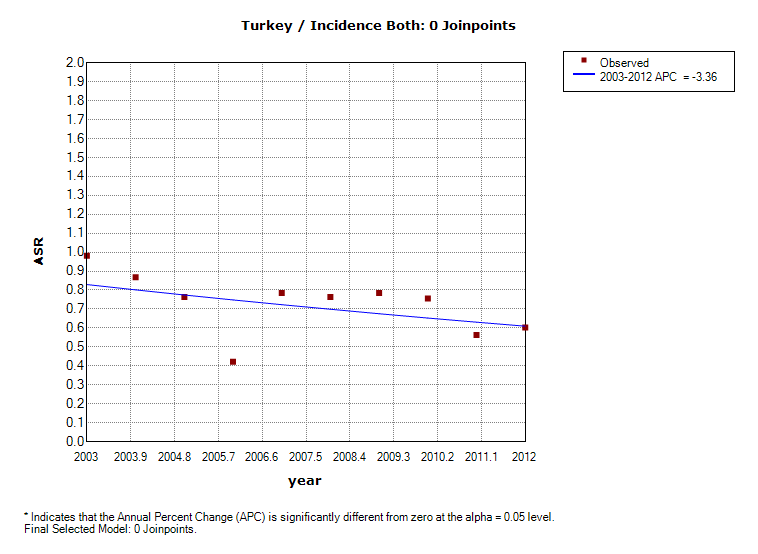 |
| **Oceania** | |
| 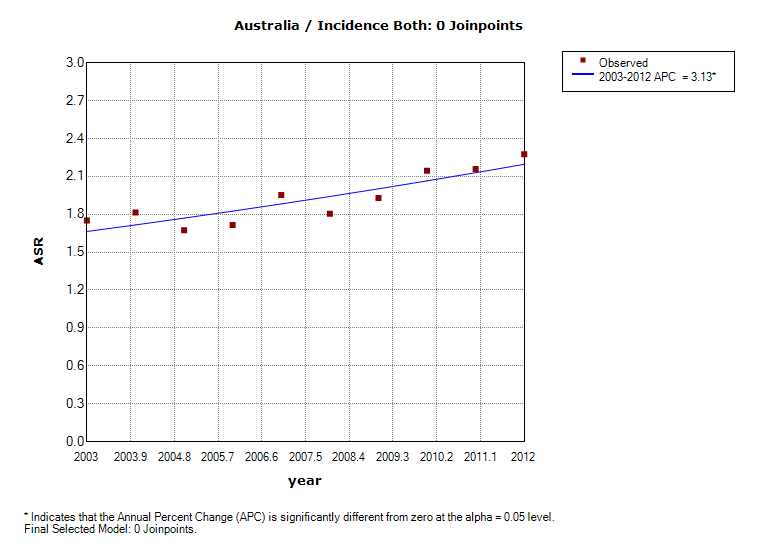 | 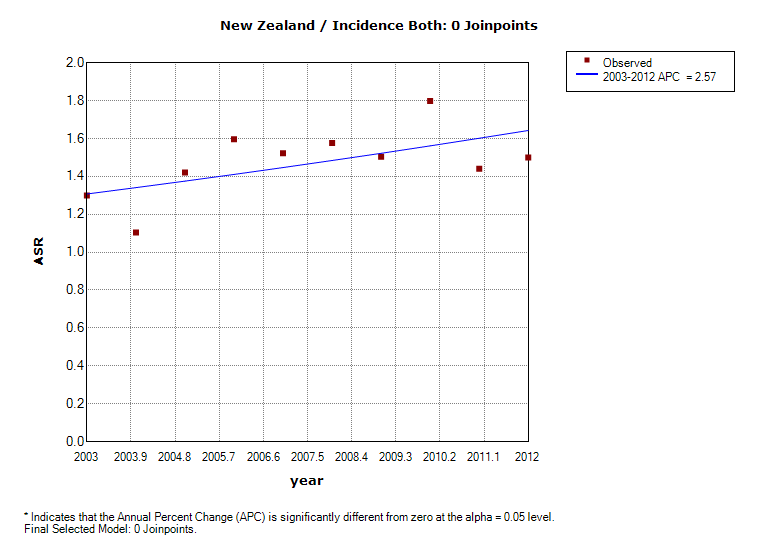 |

| **Northern America** | |
| --- | --- |
| 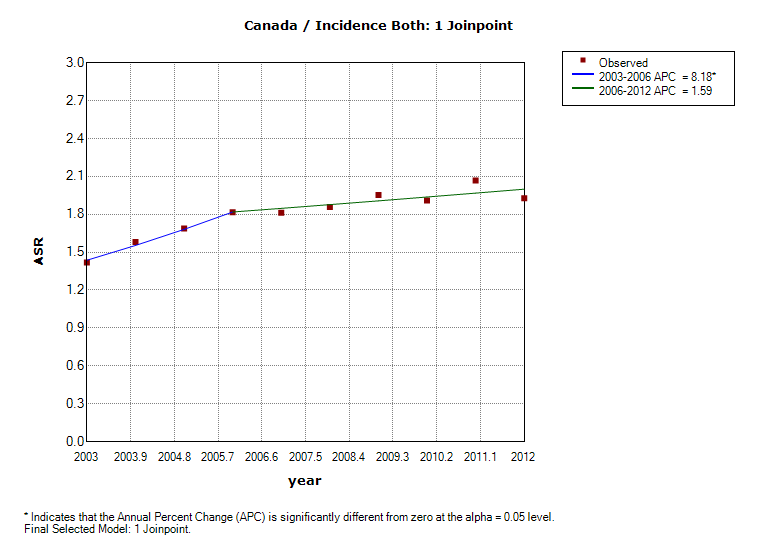 | 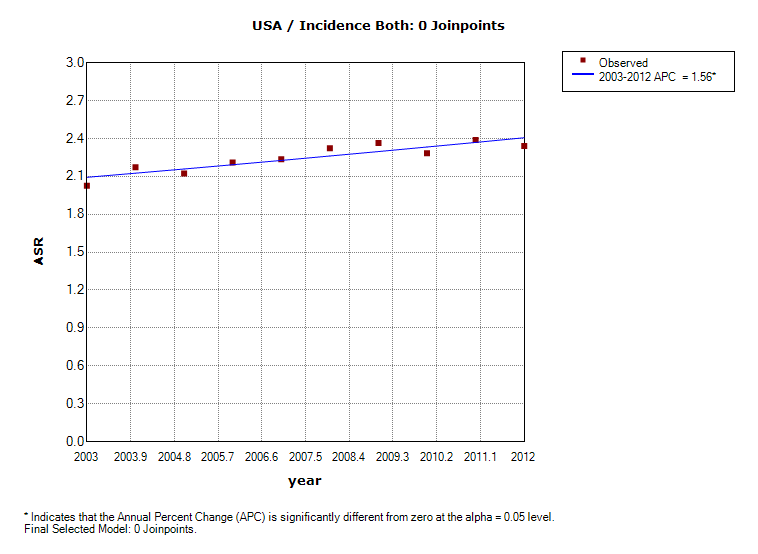 |
| **Southern America** | |
| 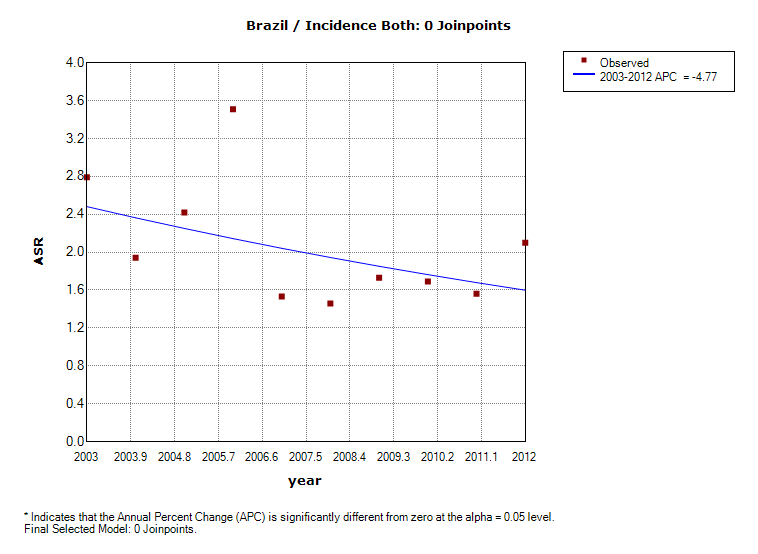 | 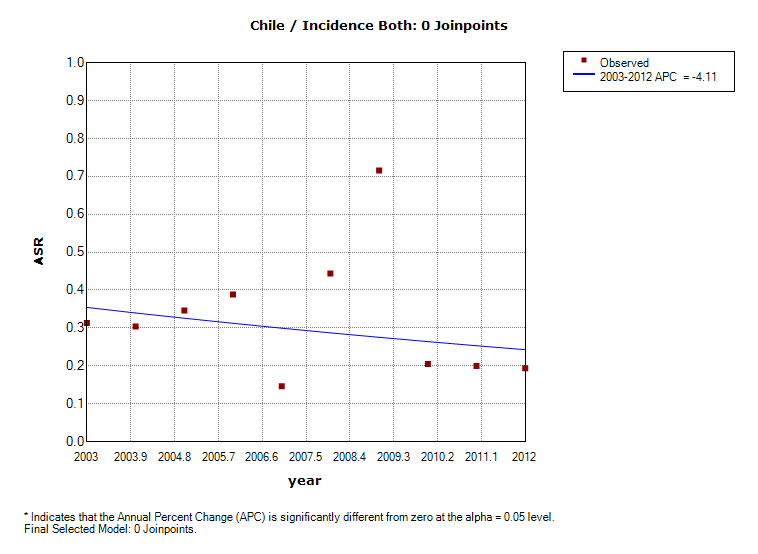 |
| 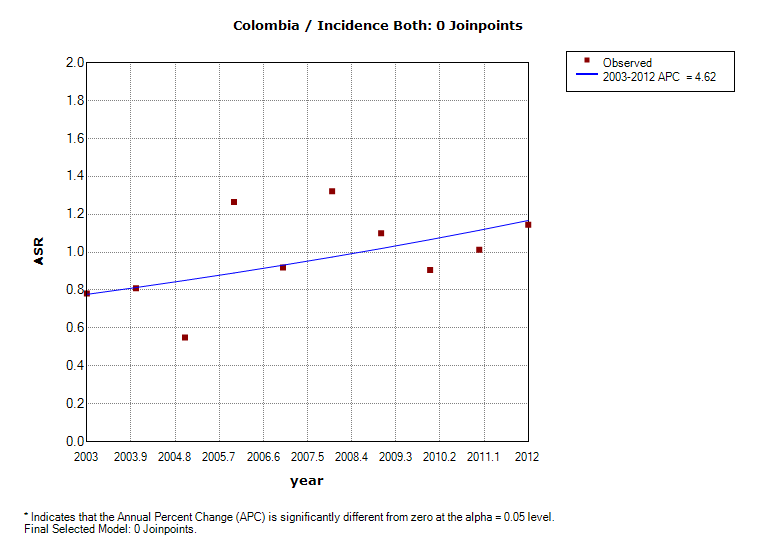 |  |
|  |  |

| **Northern Europe** | |
| --- | --- |
|  |  |
|  |  |
|  |  |
|  |  |

| **Western Europe** | |
| --- | --- |
|  |  |
|  |  |
|  |  |

| **Southern Europe** | |
| --- | --- |
|  |  |
|  |  |
|  |  |

| **Eastern Europe** | |
| --- | --- |
|  |  |
|  |  |
| **Africa** | |
|  |  |

d) Young

| **Asia** | |
| --- | --- |
|  |  |
|  |  |
|  |  |
|  |  |
|  |  |
| **Oceania** | |
|  |  |

| **Northern America** | | | |
| --- | --- | --- | --- |
|  | |  | |
| **Southern America** | | | |
|  | |  | |
|  | |  | |
|  | |  | |
| **Northern Europe** | | | |
|  | |  | |
|  | |  | |
|  | |  | |
|  | |  | |
| **Western Europe** | | |  |
|  |  | |  |
|  |  | |  |
|  |  | |  |

| **Southern Europe** | |
| --- | --- |
|  |  |
|  |  |
|  |  |

| **Eastern Europe** | |
| --- | --- |
|  |  |
|  |  |
| **Africa** | |
|  |  |

e) Old

| **Asia** | |
| --- | --- |
|  |  |
|  |  |
|  |  |
|  |  |
|  |  |
| **Oceania** | |
|  |  |

| **Northern America** | |
| --- | --- |
|  |  |
| **Southern America** | |
|  |  |
|  |  |
|  |  |

| **Northern Europe** | |
| --- | --- |
|  |  |
|  |  |
|  |  |
|  |  |

| **Western Europe** | |
| --- | --- |
|  |  |
|  |  |
|  |  |

| **Southern Europe** | |
| --- | --- |
|  |  |
|  |  |
|  |  |

| **Eastern Europe** | |
| --- | --- |
|  |  |
|  |  |
| **Africa** | |
|  |  |
